# Semantic biomedical resource discovery: a Natural Language Processing framework

**DOI:** 10.1186/s12911-015-0200-4

**Published:** 2015-09-30

**Authors:** Pepi Sfakianaki, Lefteris Koumakis, Stelios Sfakianakis, Galatia Iatraki, Giorgos Zacharioudakis, Norbert Graf, Kostas Marias, Manolis Tsiknakis

**Affiliations:** 1 Foundation for Research and Technology Hellas (FORTH), Institute of Computer Science, N. Plastira 100, Vassilika Vouton, Heraklion, Crete Greece; 2 Department of Informatics Engineering, Technological Educational Institute, Heraklion, Crete Greece; 3 Paediatric Haematology and Oncology, Saarland University Hospital, Homburg, Germany

**Keywords:** Semantic resource annotation, Natural language processing, Resource discovery, Biomedical text annotation, Information extraction, Text mining, Biomedical informatics, Search engine, Natural language interface

## Abstract

**Background:**

A plethora of publicly available biomedical resources do currently exist and are constantly increasing at a fast rate. In parallel, specialized repositories are been developed, indexing numerous clinical and biomedical tools. The main drawback of such repositories is the difficulty in locating appropriate resources for a clinical or biomedical decision task, especially for non-Information Technology expert users. In parallel, although NLP research in the clinical domain has been active since the 1960s, progress in the development of NLP applications has been slow and lags behind progress in the general NLP domain.

The aim of the present study is to investigate the use of semantics for biomedical resources annotation with domain specific ontologies and exploit Natural Language Processing methods in empowering the non-Information Technology expert users to efficiently search for biomedical resources using natural language.

**Methods:**

A Natural Language Processing engine which can “translate” free text into targeted queries, automatically transforming a clinical research question into a request description that contains only terms of ontologies, has been implemented. The implementation is based on information extraction techniques for text in natural language, guided by integrated ontologies. Furthermore, knowledge from robust text mining methods has been incorporated to map descriptions into suitable domain ontologies in order to ensure that the biomedical resources descriptions are domain oriented and enhance the accuracy of services discovery. The framework is freely available as a web application at (http://calchas.ics.forth.gr/).

**Results:**

For our experiments, a range of clinical questions were established based on descriptions of clinical trials from the ClinicalTrials.gov registry as well as recommendations from clinicians. Domain experts manually identified the available tools in a tools repository which are suitable for addressing the clinical questions at hand, either individually or as a set of tools forming a computational pipeline. The results were compared with those obtained from an automated discovery of candidate biomedical tools. For the evaluation of the results, precision and recall measurements were used. Our results indicate that the proposed framework has a high precision and low recall, implying that the system returns essentially more relevant results than irrelevant.

**Conclusions:**

There are adequate biomedical ontologies already available, sufficiency of existing NLP tools and quality of biomedical annotation systems for the implementation of a biomedical resources discovery framework, based on the semantic annotation of resources and the use on NLP techniques. The results of the present study demonstrate the clinical utility of the application of the proposed framework which aims to bridge the gap between clinical question in natural language and efficient dynamic biomedical resources discovery.

**Electronic supplementary material:**

The online version of this article (doi:10.1186/s12911-015-0200-4) contains supplementary material, which is available to authorized users.

## Background

A plethora of publicly available biomedical resources (data, tools, services, models and computational workflows) do currently exist and are constantly increasing at a fast rate. This explosion of biomedical resources generates impediments for the biomedical researchers’ needs, in order to efficiently discover appropriate resources to accomplish their clinical tasks. It is extremely difficult to locate the necessary resources [1], especially for non-Information Technology (IT) expert users, because most of the available tools are commonly described via narrative web pages containing information about their operations in natural language or are annotated with relevant technical details which are not easily interpreted by lay users. These descriptions contain plain text with no machine interpretable structure and therefore cannot be used to automatically process the descriptive information about a resource. An indicative resource and its description is shown on Table 1.

**Table 1:** An example of a resource and its description

Name	Summary	Tags (Principal bioinformatics methods)	
GeneTalk	GeneTalk, a web-based platform, that can filter, reduce and prioritize human sequence variants from NGS data and assist in the time consuming and costly interpretation of personal variants in clinical context. It serves as an expert exchange platform for clinicians and scientists who are searching for information about specific sequence variants and connects them to share and exchange expertise on variants that are potentially disease-relevant.	Genetic variation annotation, Sequence variation analysis, Variant Calling, Structural variation discovery, Filtering, Annotation, Database, Exome analysis, Sequence analysis, Variant Classification, Viewer	
Link	Input (format)	Output (format)	Category
http://seqanswers.com/wiki/GeneTalk	VCF	VCF,XLS,XLSX	Sequence Analysis

Furthermore, clinical users prefer to formulate their queries quickly using natural language which is the most user-friendly and expressive way [2]. As a result, discovery of the appropriate tools and computational models needed to support a given clinical decision making task has been and remains a major problem for non-expert users. Due to the fact that the range of accessible resources has been considerably expanded in recent years and a significant number of new such resource repositories have been developed, it has been more and more difficult for clinicians and researchers to locate the most appropriate resource for the realization of their tasks.

On the other hand, bioinformaticians and tool developers rely to a greater extent on ontologies to annotate their systems and publish them in specialized repositories, such as Taverna [3], myExperiment [4], BioCatalogue [5], SEQanswers [6], EMBRACE [7], Bioconductor [8], and ORBIT [9]. Such repositories make software components easier to locate and use when they are described and searched via rich metadata terms but act as independent silos devoted to specific domains and are unable to provide end to end solutions to daily routine clinical questions. An indicative example is SEQanswers [6], where a user can find an abundance of tools which however are restricted only to sequencing. In such repositories, the main impediments that a clinician faces are: (i) the need to serially search or search with exact keyword-matching in repositories with thousands of tools, (ii) substantial information technology (IT) knowledge is required in order for a clinician to understand a tools purpose and way of use, (iii) time consuming search in various or all the publically available repositories, and (iv) the uncertainty regarding the appropriateness of a retrieved tool for his clinical decision task [10].

In most of the cases clinical users come up with long and complex questions in the context of their hypothetico-deductive model of clinical reasoning [11]. What is equally important is the fact that clinical users are not prepared, on average, to allocate more than 2 minutes for discovering appropriate tools and usually give up if the inquiry is time consuming [12]. Furthermore, the appropriateness of the results obtained often depends on the user’s IT expertise.

The use of queries expressed in natural language can, it is believed, overcome these hurdles [13], yet computers are good at processing structured data but much less effective in handling natural language that is inherently unstructured. The field of Natural Language Processing (NLP) [14] aims to narrow this gap, as it focuses on how machines can understand and manage natural language text to execute useful tasks for end users.

A survey on biomedical text annotation tools was performed taking into account Named Entity Recognition (NER) tools that can identify biomedical categories, like gene and protein names, as well as Ontology-Based Information Extraction (OBIE) tools. Several approaches and tools were evaluated, including ABNER [15], GATE [16], UIMA [17], NCBO BioPortal [18], MetaMap [10], AutoMeta, KIM, ONTEA [19], and finally SOBA and iDocument [20] which do not support annotation with multiple ontologies or clinical text at all.

MetaMap is worthy of note as a state-of-the-art tool and the de-facto standard for biomedical annotation. This tool maps text to the Unified Medical Language System (UMLS) [21] Metathesaurus concepts. MetaMap can identify 98.19 % of the biomedical concepts in the text including 78.79 % of the concepts that manually could not be identified [22]. The tools inefficiencies are mainly due to missing entries in UMLS; furthermore, concepts’ relationships, multi-words concepts entries, words with punctuation and spelling mistakes in text are not recognized and dealt with. Therefore, minor orthographical or syntactic errors in a sentence cannot be detected. In addition, MetaMap can support only concept recognition and for specific ontologies. On the other hand, cTAKES, an Apache open source NLP system, implements rule-based and machine learning methods. The tool exhibits reasonable performance which was nevertheless inferior to the one achieved by MetaMap [23].

## The purpose of the study

Given the complexities mentioned above, the aim of the present study is to i) investigate the use of semantics for the annotation of biomedical resources with domain specific ontologies and ii) exploit NLP methods in empowering the non-IT expert users to efficiently search for biomedical resources using natural language.

Our specific focus is to capitalize on existing research results and extend these with the objective of providing to users, especially physicians, the opportunity to represent their queries in natural language and to dynamically discover and retrieve suitable candidate computational resources, with the aid of information extraction algorithms guided by specific domain ontologies.

**Fig. 1:**
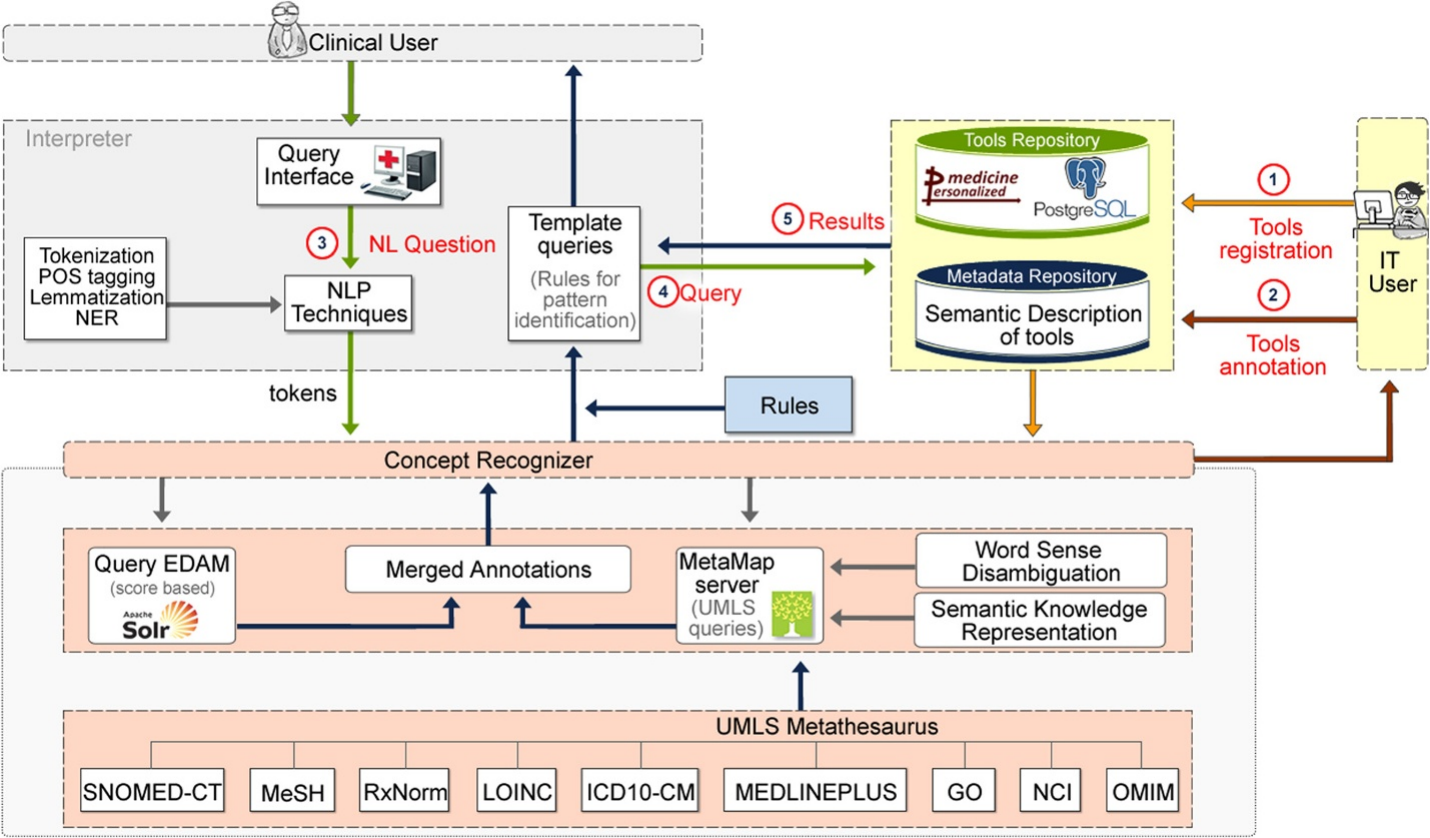
The architecture of the framework. The architecture of the framework: 1) tools registration, 2) tools annotation, 3) user’s question in natural language and NLP processing, 4) form and send the query, and 5) retrieve results (related tools)

In achieving the stated objectives, we introduce a semantic biomedical resource discovery framework based on NLP. A high level architecture of developed framework is shown in Fig. 1. The clinician can import his research question in natural language (English language) through a web interface. Then, the interpreter receives the clinical question as input, and parses the text using NLP techniques guided by the existing domain ontologies [24]. The objective at this step is to infer the question’s meaning by locating ontological terms important in the clinical domain of interest. The results of this step are then matched to a set of predefined “patterns” that produce a low level query to repository of biomedical tools and other resources. When this query is executed, the repository returns the list of tools or custom pipelines that possibly answer the initial question of the user.

In developing and evaluating our semantic biomedical resource discovery framework we have specifically focused on the following questions:

- Do existing biomedical ontologies, as well as current NLP tools, suffice for the creation of a domain-specific annotation system?
- Are existing biomedical annotation systems acceptable and satisfactory for the Semantic Annotation of biomedical concepts guided by ontologies?
- Can an interpreter translate natural language clinical questions into targeted queries using patterns and ontology terms?

In the following sections we describe details of the framework design and implementation, provide evaluation details and results, and conclude with a discussion and future work.

## Methods

The proposed framework was designed and implemented within the European Commission project p-medicine [25] as the project’s workbench which is an end-user application that is effectively a repository of tools for use by the clinicians. It also follows exploratory work that has taken place in the context of the Contra Cancrum EC funded project [26]. The objective of the workbench is to boost the communication and collaboration of researchers in Europe for the machine-assisted sharing of expertise.

In more detail the proposed framework initially performs an NLP processing step. The user’s clinical question is split into tokens which are the words and punctuation that establish the sentence; the tokens are lemmatized, i.e. the words are mapped to their roots (“lemmas”), and at the end each token is matched to a specific “Part of Speech” (POS) of the English grammar. During this pre-processing task, NER, the task that organizes the text elements into predefined categories, is also performed for each token and assigns the token to a specified category (e.g. a gene symbol). For the NER process we used the clinical, biomedical and pharmaceutical semantic types used in MetaMap (in Additional file 1: Table S5). Subsequently the system communicates with the *Concept Recognizer* (lower part in Fig. 1) and extracts ontology terms, concepts and semantic types. Using the ontology terms and its semantic types the system identifies predefined patterns and delivers focused queries to the repository of resources for an efficient discovery [27]. The identified tools/services are then presented to the end user.

The architecture of the framework, as shown in Fig. 1 integrates three main components: (i) the resource and metadata repositories, (ii) the semantic annotator (“Concept Recognizer”) and (iii) the intelligent engine (“Interpreter”) which interacts with the non-IT end user.

The following sections provide more elaborate details on the implementation and functioning of all the sub-components of the framework.

### Concept Recognizer – the semantic annotator

The core of the system is the so called Concept Recognizer that is used by most of the components of the framework. The objective of this component is, given the free text formulation of the user’s query, to extract the “important” parts, using n-grams [28], that refer to or designate known ontology terms, in order to get matched to the patterns that form the physicians’ necessities. By these patterns a query is formed and promoted to the tools repository to finally get the appropriate tools/services for the user’s task. The *Concept Recognizer* integrates two special domain ontologies: the EDAM ontology for the software domain and the UMLS biomedical ontologies for the biomedical domain. For both domains a specific concept recognizer has been implemented:

- EDAM (originally from “EMBRACE Data and Methods”) is an ontology for annotation of bioinformatics tools, resources and data. Its design principles are bioinformatics specific with well-defined scope, relevant and usable for users and annotators, and maintainable. EDAM applies to organizing and finding suitable tools or data and to automate their integration into complex applications or workflows. EDAM has been already successfully used in other systems like BioXSD [29] and Bio-jETI [30]. The EDAM concept recognizer was implemented using the Apache Solr [31] full text search server. For each term in the EDAM ontology a JSON-formatted file was created, with specific fields that were subsequently imported to Solr. The different fields give the ability to use different weights at search time. The weight formula that was used is biased to the id and the name of the term. This implies that if the searched text matches with the id or the name of a term, then this term is assigned a better score than if the text matched to the definition or the comment fields. The formula of the custom made EDAM weight is:
  $$ \left( id* 1 0\right) + \left( name* 1 0\right) + \left( synonym* 6\right) + \left( subset* 3\right) + \left( is\_a* 3\right) + \left(def* 2\right) + \left( comment* 1\right) $$
- The MetaMap concept recognizer identifies and annotates medical terms based on terminological resources included in UMLS Metathesaurus. MetaMap integrates two different NLP servers; the Semantic Knowledge Representation (SKR) server which combines contextual information with lexical information to improve the tagging accuracy and the Word Sense Disambiguation (WSD) server which involves the determination of the meaning and understanding of words. The semantic types of the UMLS and the WSD server are used to classify the terms in certain categories in order to acquire a specific meaning.

The *Concept Recognizer* integrates the MetaMap and the EDAM concept recognizers, and also reports the semantic types of the matched term which provides a consistent categorization of all concepts present in UMLS Metathesaurus or in EDAM ontology. UMLS provides more than 100 semantic categories (http://mmtx.nlm.nih.gov/MMTx/semanticTypes.shtml); 58 of them, clinical, medical, biomedical and pharmaceutical, were selected, combined and categorized in 23 semantic types, more general and comprehensive for non-experts. Four more categories of the Edam ontology were added. The full list of the selected categories is shown in Table 2.

**Table 2:** The 27 semantic categories. The 27 prime categories: 23 from UMLS semantic types and 4 from Edam categories

1. Disease	2. Drug	3. Medical Procedure	4. Tissue
5. Biomedical	6. Cell	7. Organism Function	8. Finding
9. Body Part	10. Gene	11. Clinical Attribute	12. Patient
13. Diagnosis	14. Age	15. Molecular Sequence	16. Device
17. Symptom	18. Virus	19. Injury or Poisoning	20. Vitamin
21. Laboratory	22. Food	23. Temporal Concept	
24. EDAM Data/Format	25. EDAM Topic	26. EDAM Operation	27. EDAM Identifier

When the end user posts a question, the concept recognizer applies NLP algorithms and invokes both the EDAM and the MetaMap concept recognizer. Subsequently, the results of the two concept recognizers are merged. It should be pointed out that some terms do co-exist in both the EDAM ontology and in one or more additional UMLS ontologies. In such cases the concept recognizer merges all the proposed concepts and passes them back to the interpreter as a list of proposed concepts; therefore the interpreter decides which concept would be kept with emphasis in EDAM (software ontology) terms, due to the fact that our objective is to identify tools and other software resources. The decision is based on the following rules: 1) if a concept co-exist in the “format of data” branch of EDAM and in one or more UMLS ontologies’ terms, then the EDAM term would be kept and 2) if a concept co-exist in more than one ontology terms, the term that has the higher score would be kept.

### Resource repository and ontologies

A repository of biomedical tools and services was employed that contains semantically annotated biomedical resource descriptions using the same ontologies as of the *Concept Recognizer*. The tools repository of the p-medicine workbench is based on the PostgreSQL [32] database with full text search capabilities.

The repository currently stores information for 502 tools and services that were either developed by the project itself or extracted from different domain specific repositories or from the web, as follows:

- 195 sequence analysis tools and resources from the SEQanswers [6],
- 35 biomedical tools from the Embrace [7],
- 133 bioinformatics tools and resources from Bioconductor [8],
- 75 bioinformatics tools and workflows from myExperiment [4],
- 50 biomedical tools and 50 biology related tools by searching the web.

The repository also includes a selected set of computational models, exposed as tools, that simulate disease evolution or response to treatment, such as [33] and [34]. The ontological concepts and semantic terms for the description of tools are generated automatically using scripts that take as input the textual description of the tool (as shown in Table 1) and “*feed*” the *Concept Recognizer*, which consequently extracts ontology terms and corresponding semantic categories. Using these annotations, we seek to facilitate more intelligent search results that address what a user is actually looking for, rather than simply returning candidate tools following a keyword matching process.

The tools repository supports three different strategies for resource discovery. Namely, (i) full text, i.e. a tools description is given in plain text, (ii) use of tags, i.e. user provided concepts and semantic types for the tools and their operations and finally (iii) parameters, i.e. inputs and outputs of a tool is specified. Such an approach implies that a clinical question can be annotated with ontological concepts and as a result the repository can be queried using full text, tags or semantic types of the UMLS ontologies.

### Interpreter

The *Interpreter* is the core element of the system and the bridge between the clinical question and the query formed for the resource repository. It employs NLP techniques and utilizes the *Concept Recognizer* module to formulate more focused queries to the repository. The specific NLP techniques employed are based on the Stanford CoreNLP [35] version 3.3.0. The *Interpreter* receives as input the clinical question and executes the analytical steps of tokenization, lemmatization and POS tagging; the resulting tokens are syntactically annotated and therefore can be recognized as entities and match the patterns that are connected to the output’s objective.

The final step of the NLP operations in the interpreter includes a queries’ template based on expression matching in order to extract relationship patterns between clinical entities. With these patterns (Table 2) the system identifies and categorizes parts of the input text as input/available data and parts that compose the clinical hypothesis (clinical question to be answered).

The development of specific patterns aims to identify specific relations within sentences, and support disambiguation of multiply-annotated words. For example, if in the clinical question a *Drug* category term and a *Disease* category term co-exist, as identified by the Concept Recognizer, this matches the combined pattern *Drug for Disease* where a partial meaning could be that the specific *Drug* is suitable for this *Disease*.

A specific pattern, called prime category, was created for every semantic category resulting in 23 categories for the UMLS semantic types and 4 categories for the Edam types. With these 27 prime/simple categories at hand, 24 new patterns, based on recommendations from experts, were created using combinations of the prime categories (Table 3). These combinations have a special meaning for the clinicians; for example, the pattern *“Drug”* for *“Disease”* relates to the concept of treatment for a physician.

**Table 3:** The list of patterns generated by the combination of prime categories

1. Drug for Disease	2. Edam Data/Format and Edam Operation
3. Patient took Drug for Disease	4. Finding with Organism_Function
5. Drug for Disease in Body Part	6. Finding in/with Medical_Procedure
7. Drug for Symptom	8. Edam_Data in Body_Part
9. Patient has Disease in Body Part	10. Patient took Drug for Disease in Body Part
11. Patient took Drug for Body Part	12. Patient has been in Medical Procedure
13. Patient has Disease	14. Patient has Organism Function
15. Disease in Body Part	16. Drug for Edam_Data in Body_Part
17. Patient’s Finding	18. Drug & Drug for Disease
19. Patient took Vitamin	20. Symptom of Medical_Procedure
21. Patient has Symptom	22. Drug for Edam_Data
23. Patient ate Food	24. Edam Data/Format and Edam Operation and Edam Data/Format

The system, analysing the clinical question given as input, formulates two types of focused queries. The first is based on the tagged terms, their combination and their position in the question. The second is based on the semantic type of the tagged terms, for both input and output terms.

Subsequently, the queries are passed into the tools repository, and two lists of candidate tools are exported; a list of tools that could totally address the clinical question at hand and a list of pipelined tools that could address the question sequentially. In order to export a ranked list of candidate tools based on correctness and accuracy metrics, the framework ranks the tools using a scoring mechanism as follows:

- Every tool or service gains one point for each appearance of the identified terms in the description of either the input required or the output produced by the tool.
- Every tool or service gains 0.25 points for each appearance of the term in the functional, textual description of the tool. This means that if the tagged term of the sentence matches a tagged term in the textual description of the relevant tool, a quarter of point is gained.
- Tools with a score equal or less than 1 are ignored.

Furthermore, every tool that has matched terms both from the given data sub-sentence in the description of their input and from the clinical question sub-sentence in the output produced form a list of tools/services that can individually resolve the clinical question. The remaining tools form a secondary list, i.e. a list of tools that are candidates for the formation of a computational pipeline that could provide a solution to the problem.

For comparison purposes, we performed a free text query, similar to the searching mechanism supported by traditional tools repositories, in order to compare the automated results of our system to the matched terms of the full text query. The free text query was implemented by inserting in the query interface of the tools repository the whole clinical question as a query of free text.

## Results

For the evaluation of the framework developed we followed a case study approach. Expert users and knowledge extracted from relevant available resources assisted us in formulating a series of clinically relevant questions of increasing complexity, which were the basis for our evaluation activities. The exact clinical questions and the results obtained when the proposed framework was applied are presented in what follows.

For our experiments, the following clinical questions were used:

1. *“John has lung cancer and has been treated with carboplatin which is known for toxicology adverse effects. I would like to find literature and reference related to such events for the specific drug”*.
2. *“I have the miRNA gene expression profile of Anna which is a nephroblastoma patient. I want to identify KEGG pathways which are mainly disrupted due to gene expression.”*
3. *“Patient FK is a 1.5 year old boy with bilateral nephroblastoma and his tumor is unresponsive to chemotherapy (vincristine, actinomycin-D and Doxorubicin) with no reduction in tumor size, not allowing to perform nephron sparing surgery. I would like to obtain a list of deregulated metabolic pathways in the tumor from gene expression data in combination with miRNA data to find possible targets that can be treated with available drugs.”*
4. *“Patient SM is a 3 year old girl with metastatic nephroblastoma and miRNAs from blood are analyzed at the time of diagnosis. I would like to compare the results of miRNAs with miRNAs of the cohort of patients with metastatic nephroblastoma that are correlated to histology, treatment response and outcome to get an individual risk index of the patient including proposed pathology, treatment response and outcome.”*
5. *“Patient AB is a 5 year old boy just diagnosed with acute lymphoblastic leukemia, while immunophenotype and gene expression data as well as clinical data at the time of diagnosis are known. I would like to compare his gene expression data with the group of all patients having the same immunological phenotype.”*
6. *“Patient AB is a 5 year old boy just diagnosed with acute lymphoblastic leukemia, while immunophenotype and gene expression data as well as clinical data at the time of diagnosis are known. I would like to know the difference in gene expression between those predicting relapse and those predicting poor MRD for the different immunophenotypes. The results should be visualized.”*

We evaluated the system’s performance using precision and recall measurements. To measure precision and recall, expert physicians and bioinformaticians together went through the catalog of all the tools available in the repository, read their descriptions, functionalities and capabilities and manually identified those tools that could answer or partially answer the specific clinical question. We present in detail the results obtained when processing the first two clinical questions as indicative case studies.

The first clinical question is a combination of sentences based on descriptions of clinical trials from the ClinicalTrials.gov registry [36] and the contribution of physicians. It was imported in our system through the web interface (http://calchas.ics.forth.gr/) where it was divided into two specific contexts.

The first sentence represents the available knowledge (given data/statement) of the clinician and mainly correlates to a tool’s inputs, i.e. *“John has lung cancer and has been treated with carboplatin which is known for toxicology adverse effects.”*, while the second sentence is the clinical hypothesis, the research question, and is mainly connected to a tool’s outputs, i.e. “*I would like to find literature and reference related to such events for the specific drug”.*

**Fig. 2:**
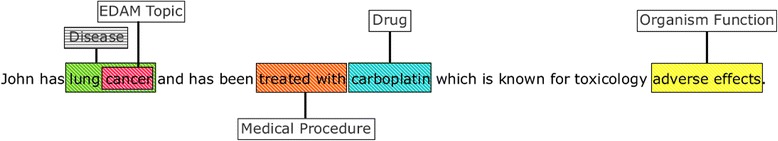
Annotation example from Concept Recognizer. The annotation from the Concept Recognizer of the given data sentence “John has lung cancer and has been treated with carboplatin which is known for toxicology adverse effects”

A visual representation for the annotation of the Concept Recognizer for the given data sentence is shown in Fig. 2, and similar annotation exists for the sentence that includes the clinical question. As explained earlier, in the case of co-existence of two annotations, the system selects the assignments that have the higher score.

The domain experts manually searched the tools repository, using the available tools descriptions, and have identified the “*EUADR - Literature analysis*” tool as a resource able to answer the specific clinical question. Table 4 shows the results of the framework for the first clinical question while in Additional file 1: Figure S1 we can see the results as shown in the web site of the NLP framework.

**Table 4:** Results of the first clinical question. The results given by the framework to the first clinical question. The list of individual tools that could solve the entire clinical question are listed at the top which are then followed by a list of the tools that could be combined, i.e. pipelined, for providing an answer to the given clinical question

Unique Tools List		
SCORE	TOOL NAME	Identified (query)
4.75 = 3 (in) + 1 (out) + 0.75 (tag)	National Cancer Institute SEER API	carboplatin & cancer (in)
		cancer (in)
		lung cancer (in)
		drug (out)
4 = 3 (in) + 1 (out)	cBio Cancer Genomics Data Server	carboplatin & cancer (in)
	(CGDS) API	cancer (in)
		lung cancer (in)
		find (out)
4 = 1 (in) + 3 (out)	EUADR - Literature analysis	adverse effects (in)
		drug-references (out)
		drug (out)
		literature (out)
Pipeline Tools List		
FIRST TOOL	SECOND TOOL	
National Cancer Institue caDSR API	AIDSinfo API	
China Cancer Database API	AIDSinfo API	
Single Tools List		
SCORE	TOOL NAME	
3.75 = 3 (in) + 3*0.25 (tag)	The Cancer Genome Atlas API	
3.75 = 3 (in) + 3*0.25 (tag)	China Cancer Database API	
3 (in)	National Cancer Institue caDSR API	
3 (in)	MuTect	
2.25 = 1 (out) + 5*0.25 (tag)	Lexicomp API	
2 (out)	Arabidopsis thaliana Microarray Analysis	
2 (out)	Pathways and Gene annotations for QTL region	
2 (out)	SciBite API	
2 (out)	DGIdb API	
2 = 4*0.25 (tag)	DailyMed API	
2 = 4*0.25 (tag)	Aetna CarePass API	
2 = 4*0.25 (tag)	National Institute on Drug Abuse Drug Screening Tool API	

As can be seen, the framework identified 23 relevant tools. Additionally, we performed a free text query, using the whole sentence as input into the tools repository, in order to compare the automated results of our system to those obtained with a full text query (a complete list of the full text results can be found in Additional file 1: Table S2).

The framework was able to identify tools that could individually address the clinical question. Such tools are listed in Table 4, and include the cBio Cancer Genomics Data Server (CGDS) API [37], the National Cancer Institute SEER API [38] and EUADR - Literature analysis [39]. EUADR is the only tool selected by the domain experts as an appropriate tool for answering the clinical question. Detail description of these three tools can be found in the Additional file 1: Table S1. There were additional tools identified that partially matched either the input or the output description; the framework performs a check of the output data types of the candidate tools for answering the input sentence and the input data types of the candidate tools that could solve the output sentence. For every data type match, a proposed pipeline is created; this implies that the user could use the first tool, and then provide its output as an input to the second tool, and so on in order to obtain an answer to the entire clinical question.

In addition, we analysed the results of the queries and measured the precision and recall of the results, as shown in Table 5: Precision and recall for the first clinical question.

**Table 5:** Precision and recall for the first clinical question. Precision and recall of the automated resource discovery in attempting to find solutions to the first clinical question as compared to results manually identified by domain experts based on the description of the tools

	Tools identified	Precision (%)	Recall (%)	#Best rank of tools that can solve the question at once (no pipelines)
Free Text	164	40	73	3 out of 164
NLP Framework	11	100	14	1^st^

Precision is the fraction of retrieved tools that are indeed relevant, while recall is the fraction of relevant tools that are indeed retrieved [40]. Both precision and recall are therefore based on an understanding and measure of relevance in our results. In order to measure the precision and recall of the automated results, domain experts manually identified 76 tools that could answer, individually or as part of a computational pipeline, the specific clinical question. Among them, the “*EUADR - Literature analysis*” tool was able to answer the specific clinical question by itself. The rest of the tools could only provide partial solutions, meaning that two or more should be pipelined for obtaining an answer.

In our first case study the true positive elements, i.e. elements that were correctly selected by the system are 11, while the false positive elements, i.e. the elements that were wrongly selected are 0, and the false negative elements, i.e. the elements that were correct but not selected – are 65 (76–11). This results in 100 % precision and 14 % recall.

As seen, the framework retrieved tools from the repository with a precision of 100 %, meaning that the system might not have exported all the suitable tools – tools that could solve partially and at once the question - for the clinical question (i.e. has low “recall”). On the other hand, what we feel is important, is the fact that all identified tools are appropriate as candidates for answering the clinical question. On the contrary, the free text query had high recall, meaning that many irrelevant tools were exported. We further discuss these findings in the discussion section.

We have subsequently employed our framework with the clinical question “*I have the miRNA gene expression profile of Anna which is a nephroblastoma patient. I want to identify KEGG pathways which are mainly disrupted due to gene expression.”* Domain experts again searched the tools repository and manually discovered that the specific sentence could be answered by the “*mirPath*” [41] or “*miRNApath*” [42] tools; it could also be answered with a combination of tools which had to contain the “*mirtarbase*” [43] tool and the “*MinePath*” [44] tool. Specifically a clinician should first use the “*mirtarbase*” tool and provide its output as an input the “*MinePath*” tool in order to resolve the full clinical question at hand.

We set the clinical question to the framework and a list of proposed tools suitable for the solution exported. The free text query was also invoked, in order to compare the framework’s results to the matched terms of the full text query. The results of the framework for the specific sentence are shown in Table 6.

**Table 6:** Results for the second clinical question. The results given by the framework to the second clinical question. The list of individual tools that could solve the entire clinical question are listed at the top which are then followed by a list of the tools that could be combined, i.e. pipelined, for providing an answer to the given clinical question

Unique Tools List		
SCORE	TOOL NAME	Identified (query)
3 = 1 (in) + 2 (out)	miRNApath	mirna (in)
		gene expression (out)
		kegg pathways (out)
3 = 1 (in) + 2 (out)	mirPath	mirna (in)
		gene expression (out)
		kegg pathways (out)
3 = 1 (in) + 2 (out)	mirtarbase	mirna (in)
		gene expression (out)
		kegg pathways (out)
No results found on this category ‘Pipeline Tools List’ for the given question		
Single Tools List		
SCORE	TOOL NAME	
4 (out)	Get Pathway-Genes and gene description by Entrez gene id	
4 (out)	Arabidopsis thaliana Microarray Analysis	
4 (out)	MinePath	
4 (out)	EnrichNet API	
4 (out)	NCBI Gi to Kegg Pathway Descriptions	
4 (out)	MitoMiner API	
4 (out)	BiologicalNetworks API	
4 (out)	From cDNA Microarray Raw Data to Pathways and Published Abstracts	
4 (out)	HUMAN Microarray CEL file to candidate pathways	
4 (out)	ERGO Genome Analysis and Discovery System	
4 (out)	BioCyc API	
4 (out)	Mouse Microarray Analysis	

The “*mirtatbase*”, “*mirPath*” and “*miRNApath*” tools were identified by the framework as top ranked tools appropriate for individually answering the clinical question. From these tools, “*mirPath”* and “*miRNApath*” were also selected by the domain experts. Details about these three tools can be found in the Additional file 1: Table S3. The “*mirtatbase*” tool was identified incorrectly as a candidate while the “*MinePath*” tool was also incorrectly identified as one of the tools that could partially answered the clinical question. Additional tools were also identified as candidates for a partial answer to the question, i.e. appropriate for solving the input or the output sentence; these tools could again form a pipeline in order to answer the whole clinical question. From these tools, the domain experts identified only one potential pipeline using the “*mirtatbase*” and “*MinePath”* tools. The results of applying our NLP framework with the second clinical question are shown in Table 6. The framework identified 17 relevant tools. We also compare the results with a full text search (complete list of the full text search results can be found in Additional file 1: Table S4).

The results of the queries were analysed and measured as shown in Table 7. Domain experts manually identified 99 tools that could solve partially or at once the specific clinical question. The NLP framework demonstrates good precision for this question too. The true positive elements are 17, while the false positive elements are 0, and the false negative elements are 82 (99–17). This gives us a 100 % precision and 17 % recall.

**Table 7:** Precision and recall for the second clinical question. Precision and recall of the automated resource discovery in attempting to find solutions to the second clinical as compared to results manually identified by domain experts based on the description of the tools

	Tools identified	Precision (%)	Recall (%)	#Best rank of tools that can solve the question at once (no pipelines)
Free Text	231	25	59	2 out of 231
NLP Framework	17	100	17	1^st^ & 2^rd^

## Discussion

This study focused on the development of a Semantic Biomedical Resource Discovery Framework by making use of natural language processing techniques. As originally stated, the envisioned framework should allow searching through a set of semantically annotated resources in order to find a match with a user query expressed as a natural language statement.

In parallel to seeking an answer to our ultimate research question, a range of additional, more specific research questions were also established. In the current section we critically discuss our experiences and the experimental evidence obtained in the context of those specific research questions initially established. We would like to stress that evaluation of the proposed approach used a limited number of queries. As a result, the present work should be seen as a case study, providing initial evidence on the validity of the approach. It is obvious that subsequent formal evaluation should be designed to test the broader effectiveness of the system.

Having said this, the experience obtained through the annotation of a large number of resources (Additional file 2), that were brought into our platform for experimentation, shows that the range of existing open biomedical ontologies and other open, generic ontologies do suffice for the creation of a domain-specific annotation framework that would be useful for the semantic resource annotation. We were able to notice the efficiency of the current software related ontology, i.e. EDAM and other biomedical ontologies that we used. Hence, we believe that there is no need for the development of a core domain ontology to enable the creation of an annotation framework that would offer capabilities of capturing the context of complex biomedical resources. Rather the challenge lies on the articulate use and integration of various existing biomedical and other related ontologies. This, nevertheless, remains a scientifically and often technically demanding task.

Our work performing NLP processing on complex biomedical text reaffirmed the various challenges identified in prior research, namely i) Clinical text has uncommon structure and content that are not always guided by grammar, syntactic or spelling rules [45], ii) Biomedical terms are prone to ambiguity; words that may have multiple meanings or many words may have the same meaning [46]; temporal ambiguity also exists, confusing past or future diagnosis or medical history, iii) Clinical content is full of abbreviations and titles that confuse the detection of a sentence’s boundary [45], iv) Negations are very common in clinical text, such as *no*, *without*, *not* and *denies* [47].

Although these challenges were evidently present in our experimentation, the range of existing NLP tools is also large. Numerous NLP packages have been also developed, such as Python NLTK, OpenNLP, Stanford NLP, LingPipe. In our work we selected the probabilistic Stanford NLP tools, where the corpus data is gathered and manually annotated and then a model is trained to try to predict annotations depended on words and their contexts through weights. The selected NLP tools for our work, with minor extensions and customization done, have proven adequate for supporting the NLP tasks of our work.

In the context of our research a limited number of clinical questions were examined. In the first research question, presented in detail in this manuscript, the framework identified the pattern “<*Drug >* for < *Disease>”,* which has a specific meaning of *Treatment* for the clinicians. According to the given input sentence, we managed to identify patterns with the combination of the annotated tagged terms of the sentence. Many more patterns can be formed and enrich the framework in the future, depending on different kind of domain searches and distinct meanings for the physicians.

Domain experts explored the tools repository and manually identified 76 tools and services (out of 502) that could provide an answer to the clinical question; some of those could give a solution individually, while others could partially solve the question.

The second clinical question presented in this manuscript led us to the matching pattern *Patient* has *Disease* and *EDAM Topic* for *EDAM Data*.

The user seeks to find the disrupted KEGG pathways according to the profile of a patient that has nephroblastoma. A tool or service or a pipeline of tools is needed to resolve this question. The domain experts manually selected 99 tools and services that could be part of the solution space. The framework’s results showed a 100 % precision and were less than the tools selected from the domain experts. In addition, the free text query exported 231 tools and identifies only 2 of the tools that according to domain experts can solve the entire clinical question.

Furthermore, in relation to execution performance, the framework proved to be able to respond fast enough and could, therefore, be used as an online search engine for biomedical tools. The response times for different clinical questions vary from 1.5 to 7.5 s which is an acceptable time for a web application. Being more specific the response time for the first clinical question is 3993 milliseconds and for the second clinical question is 7038 milliseconds. In our current implementation we use ten ontologies but the framework can be extended to use more ontologies either from UMLS or as new systems (concept recognizers) using the Solr implementation of EDAM. Initial evidence indicates that the proposed framework is scalable and can be expected to be responsive in real time even for tens of thousands of tools in the repository and with much more ontologies, due to the fact that the concept recognizer and the queries to the repository are based on elastic search which is suitable in even more demanding domains such as big data applications [48].

### Future work

We plan to extend the framework and provide end users with options to create and import new patterns through the web interface, that may be needed and do not already exist. We also explore methodologies for personalized preferences with classification based on user profile [49] and “voting” mechanism on the retrieved results in order to improve our accuracy in similar user groups.

Another direction under investigation is to enrich the framework with graph theory capabilities and provide the end user possible workflows [50], for the solution of the research question. Such methodologies proved to be valuable in services discovery [51] and scientific workflows composition [13, 52–54]. Taking advantage of the modular implementation and the rich metadata schema of the NLP framework we expect to provide meaningful pipelines as guidelines to complex clinical questions. Again a high quality annotation of the tools in the repository is mandatory for accurate results.

In addition, the implementation of this framework will be expanded with even more patterns focused on all the possible combinations of the semantic categories of the EDAM software ontology and the clinical ontologies of the UMLS Metathesaurus, in order to create more accurate patterns with clinical meanings, taking into account the ontology-based relationships of concepts and how they map to similar structures in natural language expressions, as we expect that soon the tools repository will host thousands of software resources. We also plan to add negation detection patterns to identify diagnosis and symptoms that are negated. In that direction, we will evaluate and possibly use opinion mining methodologies able to categorize the polarity of a text, meaning if the sentence or word is positive, negative or neutral. Solutions like the “Crowd Validation” [55] which examine and determine opinions, perceptions and approaches along with NLP methodologies for ontology management and query processing [56–58] will be possibly used.

In addition, the key functions of clinical decision support systems require understanding of the context from which an event or a named entity is extracted. For example, supporting clinical diagnosis and treatment processes with best evidence will require not only recognizing a clinical condition, but determining whether the condition is present or absent. Chapman et al. [59] developed a dedicated algorithm, ConText, for identifying three contextual features: Negation (for example, no pneumonia); Historicity (the condition is recent, occurred in the past, or might occur in the future); and Experience (the condition occurs in the patient or in someone else, such as parents abuse alcohol). In many cases also it is desirable to detect the degree of certainty in the context (for example, suspected pneumonia). Although significant results in relation to the topic of context exist, e.g. Solt and colleagues [60] described an algorithm for determining whether a condition is absent, present, or uncertain it is our view that the related issues will continue to present researchers with challenges.

Another challenge for the future relates to multilingualism; taggers, parsers and lexicons for additional languages, apart from English, could be added into the system and provide a service discovery framework for a multilingual setting.

Additionally, the system could, eventually, be extended to a question-answer system. A question driven system could upgrade the system to the next step of an intelligent service discovery system, by asking the user a question that was retrieved from the input sentence. In this way the system could provide the user services or tools in pipeline that could be used in a row for the desired process to be implemented.

Furthermore, in the context of the p-medicine EC project, a thorough usability evaluation of the system from end users has been scheduled in order to assess the usability and the acceptance of the framework.

## Conclusions

The ultimate objective of this work has been to investigate the use of semantics for biomedical resources annotation with domain specific ontologies and exploit Natural Language Processing methods in empowering the non-Information Technology expert users to efficiently search for biomedical resources using natural language.

As part of this case study, we have successfully implemented a web based framework able to interact with the end user through natural language for biomedical resources discovery in real time. The user describes in natural language the facts (data) and the research question which are analysed with NLP techniques, annotated with clinical and software ontologies in order to form specific queries for the tools repository and finally retrieve tools/services that solve the clinical question.

The results obtained showed that the system has high precision and low recall, which means that the system returns essentially more relevant results than irrelevant. There were cases of input queries that have 100 % precision in our results, meaning that the exported resources were all correct; the system may not have retrieved all the tools that could solve the clinical question, but all the retrieved tools were suitable in addressing the query. Research in web search engines indicates that 91 % of searchers do not go past page one (ten top ranked) of the search results and over 50 % do not go past the first 3 results on page 1 [61]. We expect that the same behaviour holds in tools repositories too, since web search has impact on the way we search and retrieve information [62]. Having a system which is able to narrow down the retrieved results with 100 % precision and provide a good ranking would be valuable for the end users especially the ones who stick to the top ranked results and neglect the rest.

Comparing MetaMap with clinical annotators like, GATE (General Architecture for Text Engineering) [16], Apache Stanbol – IKS (Interactive Knowledge Stack) [63], NCBO Annotator (BioPortal) [64] and ConceptMapper [65], we concluded that MetaMap is the best biomedical concept recognizer for our needs, because it has a RESTful API which can use many clinical ontologies that are connected to it, while the rest of the clinical annotators were not able to be loaded with a large number of ontologies that were needed in our case.

We must accept that searching with ontology terms provided better results than with the semantic types of the terms; this is firstly an effect of the tag queries’ dependence on the tagged terms identified in the sentence which are also terms of the tools descriptions, and secondly, due to the fact that the semantic types of the terms may not be found in the descriptions, but even if they do, they have a minor score priority.

The proposed NLP framework has the potential to aid physicians practice advanced ICT medicine and improve the quality of patient care. To our knowledge a plethora of tools can handle clinically relevant information for specific questions but are limited to provide summaries of literature such as AskHermes [11]. It is also known that a plethora of databases and specialized repositories exist and numerous clinical and biomedical tools have been indexed in these repositories, but the searching mechanisms and the technical terminology used, discourage clinicians to use them.

We implemented a web based framework taking advantage of domain specific ontologies and NLP in order to empower the non-IT users to search for biomedical resource using natural language. The proposed framework links the gap between clinical question and efficient dynamic biomedical resources discovery. Given the experience we gained during the design, implementation and set up of such a framework we can safely come into the conclusion that there is adequacy of existing biomedical ontologies, sufficiency of NLP tools and biomedical annotation systems for the implementation of such a framework.

## Additional files

Additional file 1: **contains extensive results of the experiments.** (PDF 803 kb)

Additional file 2: **contains the list of tools and resources that were in our repository.** (PDF 964 kb)

## Abbreviations

IT: Information Technology
NLP: Natural Language Processing
POS: Part Of Speech
NER: Named Entity Recognition
OBIE: Ontology-Based Information Extraction
UMLS: Unified Medical Language System
SKR: Semantic Knowledge Representation
WSD: Word Sense Disambiguation
