# Supplementary material for: Semantic biomedical resource discovery: a Natural Language Processing framework
Source: BMC Med Inform Decis Mak. 2015 Sep 30;15:77. doi: 10.1186/s12911-015-0200-4 (PMC4591066; doi:10.1186/s12911-015-0200-4)
Supplement: Supplementary file 1 — contains extensive results of the experiments. (PDF 803 kb) [file 12911_2015_200_MOESM1_ESM.pdf]

Table 1: The descriptions of the tools that could solve entirely the first clinical question.

SCORE	Tool Name	Tool URL	Tool Description	Input	Output
4	cBio Cancer Genomics Data Server (CGDS) API	http://www.programmableweb.com/api/cbio-cancer-genomics-data-server-cgds	The service provides direct access by applications to genetic and genomic data stored by the Memorial Sloan-Kettering Cancer Center. Applications can retrieve information about cancer studies and their genetic findings, genetic profiles and indicators for specific cancer types and gene structures, and similar highly detailed data. Datasets document more than 5000 tumor samples from 20 research studies. API methods support retrieval of cancer research studies, with detailed filtering by specific topics and findings. Methods also allow retrieval of genetic profiles and case lists reported in cancer research, extended mutation data, protein and antibody information, and clinical data.	genetic data, genomic data, cancer, tumor	cancer, genetic finding, genetic profile, gene structure,mutation data, protein, antibody information, clinical data,Tab-delimited text

4	National C ancer Insti tute SEER API	http://www.programmableweb.com/api/national-cancer-institute-seer	The Surveillance, Epidemiology and End Results (SEER) Program, a service of the National Cancer Institute, is a cancer statistics resource. Collected data includes information on the incidence, prevalence and survival from specific geographic areas within the U.S., as well as cancer mortality for the entire country. The SEER API is a RESTful service supporting various program datasets and algorithms, and is available to developers who wish to incorporate SEER resources into their own systems. Exposed resources include Collaborative Staging, the Hematopoietic and Lymphoid Neoplasm Database, NAACCR documentation, the Antineoplastic Drugs Database, and incidence site recode variables. The API returns JSON formatted responses, communicates over HTTPS, and requires a free account and API key.	cancer, cancer mortality	antineoplastic drugs, hematopoietic, lymphoid neoplasm, JSON

4	**EUADR - Literature analysis**	http://www.myexperiment.org/workflows/2280.html	The aim of the "Literature Analysis" workflow is to automate the search of publications related to ADRs corresponding to a given drug/adverse event association. To do so, we defined an approach based on the MeSH thesaurus, using the subheadings «chemically induced» and «adverse effects» with the "Pharmacological Action" knowledge.	Toxicology Adverse effects (topic_2840), Drug identifier (data_0993)	Literature and reference (topic_3068)

Table 2: The free text tools that were exported automatically by the framework for the first research question.

Free Text Tools	
158	1. Correlation service<br>2. Drug Re-Purposing Workflow<br>3. EUADR – Literature analysis<br>4. Epidemic Marketplace API

	5. ADR-S<br>6. MiMI/Metabolomics API<br>7. Sierra Stanford HIV API<br>8. GSNAP<br>9. cBio Cancer Genomics Data Server (CGDS) API<br>10. tspair<br>11. iASeq<br>12. FastA protein similarity search API<br>13. DGIdb API<br>14. Predictionet<br>15. SciBite API<br>16. From cDNA Microarray Raw Data to Pathways and Published Abstracts<br>17. VirusSeq<br>18. National Cancer Institute SEER API<br>19. SLAP API<br>20. geneRecommender<br>21. bioNMF API<br>22. China Cancer Database API<br>23. Download pathways for external references list<br>24. FusionMap<br>25. Tracknburn FoodData API<br>26. BSMAP<br>27. ViralFusionSeq<br>28. ObTiMA<br>29. Oncosimulator<br>30. ChEMBL API<br>31. National Library of Medicine ChemSpell API<br>32. SomatiCA<br>33. Download pathways for external references list (Taverna 2)<br>34. INOH Pathway Database API

	35. Mirtarbase<br>36. BIOBASE API<br>37. National Drug File-Reference Terminology API<br>38. Drugle API<br>39. National Cancer Institue caDSR API<br>40. Get pathways by external reference<br>41. GBSeq test<br>42. MethylCoder<br>43. SSearch protein similarity search API<br>44. Snm<br>45. Copa<br>46. PcProf predict physico-chemical profiles of proteins API<br>47. DAVID Bioinformatics API<br>48. HIPAASpace API<br>49. DIAL<br>50. Vsn<br>51. Sizepower<br>52. Retrieve Pathways and Compound information from KEGG<br>53. MetMap<br>54. Perform a search through NCBI eUtils eSearch<br>55. Kegg pathway diagrams<br>56. BFAST<br>57. National Institute on Drug Abuse Drug Screening Tool API<br>58. Gviz<br>59. Protein search fetch align tree<br>60. BioCyc API<br>61. MuTect<br>62. Novocraft<br>63. MiRProf<br>64. CLCbio Genomics Workbench

	65. GSVA<br>66. GeneCoDis2 API<br>67. ACME<br>68. EBI IntAct<br>69. JasparDB API<br>70. SOCS<br>71. VegaMC<br>72. VIOLIN API<br>73. SIMPA96 API<br>74. BioAID Discover proteins from text plus synonyms<br>75. H-InvDB API<br>76. Lexicomp API<br>77. Charm<br>78. maSigPro<br>79. FusionCatcher<br>80. Workflow for Protein Sequence Analysis<br>81. EpiGRAPH<br>82. RAWverna Image Evaluator - Evaluate relative<br>83. Bcbio-nextgen<br>84. Spiral Genetics<br>85. RxNorm Prescribable API<br>86. CIPF RENATO API<br>87. metaArray<br>88. get drugs by pathways<br>89. Biosemantics ACCCA API<br>90. coGPS<br>91. bgafun<br>92. MassArray<br>93. Genometa<br>94. arrayQualityMetrics

	95. biomvRCNS<br>96. FragGeneScan<br>97. Cufflinks<br>98. Aetna CarePass API<br>99. Translate Nucleotide sequence into Peptide sequence<br>100. new drug test<br>101. GeneTalk<br>102. Genefu<br>103. ZINBA<br>104. DeFuse<br>105. GOR protein secondary structure prediction API<br>106. Lumi<br>107. Bis-SNP<br>108. MiRDeep<br>109. GenomicTools<br>110. Get Pathway-Genes and gene description by Entrez gene id<br>111. DailyMed API<br>112. Rdiff<br>113. Dexus<br>114. ERNE<br>115. ShortFuse<br>116. STEPStools API<br>117. CleaveLand<br>118. Olego<br>119. Sherman<br>120. Kegg DrugID<br>121. Methpipe<br>122. RMAP<br>123. Protein alignment transmembrane<br>124. COILS predict protein coiled-coil regions API

	125. GeneCruiser API<br>126. SIM<br>127. OpenTox API<br>128. How are you API<br>129. HIV Drug Research Center API<br>130. FindPeaks 4.0 (Vancouver Short Read Package)<br>131. Image Mining with RapidMiner<br>132. SignalP/SignalP4 API<br>133. The Cancer Genome Atlas API<br>134. GenomeGraphs<br>135. NCBI Conserved Domain Database (CDD) API<br>136. myHealthAccount API<br>137. BioAID ProteinDiscovery filterOnHumanUniprot perDoc html<br>138. Pseudoviewer API<br>139. RxNorm API<br>140. Cigna Health API<br>141. biocGraph<br>142. Kissnp<br>143. ncRNA fRNAdb API<br>144. Biopieces<br>145. Bismark<br>146. miRNApath<br>147. Find pathways in which two genes co-occur (in WikiPathways)<br>148. Allen Brain Atlas API<br>149. AIDSinfo API<br>150. CNANorm<br>151. maDB<br>152. CancerMutationAnalysis<br>153. FastQ Screen<br>154. BRAT

	155. MetNet API<br>156. BS Seeker<br>157. Pillbox API<br>158. HUMAN Microarray CEL file to candidate pathways

Table 3: The descriptions of the tools that could solve entirely the second clinical question.

SCORE	Tool Name	Tool URL	Tool Description	Input	Output
3	miRNApath	http://www.bioconductor.org/packages/release/bioc/html/miR	miRNApath: Pathway Enrichment for miRNA Expression Data. This package provides pathway enrichment techniques for miRNA expression data. Specifically, the set of methods handles the many-to-many relationship between miRNAs and the multiple genes they are predicted to target (and thus affect.) It also handles the gene-to-pathway relationships separately. Both steps are designed to preserve the additive effects of miRNAs on genes, many miRNAs affecting one gene, one miRNA affecting multiple genes, or many miRNAs affecting many genes.		

		NApath.html			
2	**mirPath**	http://diana.imis.athena-innovation.gr/DianaTools/index.php?r=mirpath/index	DIANA-miRPath is a miRNA pathway analysis web-server, providing accurate statistics, while being able to accommodate advanced pipelines. miRPath can utilize predicted miRNA targets (in CDS or 3'-UTR regions) provided by the DIANA-microT-CDS algorithm or even experimentally validated miRNA interactions derived from DIANA-TarBase v6.0. These interactions (predicted and/or validated) can be subsequently combined with sophisticated merging and meta-analysis algorithms.miRPath v2.0 can perform advanced analysis pipelines, such as hierarchical clustering of miRNAs and pathways based on the levels of their interactions. Furthermore, users can easily create heat maps of miRNAs vs pathways interactions. Other supported features include the identification of pathological single nucleotide polymorphisms (SNPs) in miRNA binding sites, as well as the "Reverse Search	miRNA IDs (data_2642)	Pathway or network identifier (data_1082)

			module", where the user can identify all the predicted or experimentally validated miRNAs significantly targeting a selected pathway.		
3	mirtarbase	http://mirtarbase.mbc.nctu.edu.tw/	miRTarBase has accumulated more than fifty thousand miRNA-target interactions (MTIs), which are collected by manually surveying pertinent literature after data mining of the text systematically to filter research articles related to functional studies of miRNAs. Generally, the collected MTIs are validated experimentally by reporter assay, western blot, microarray and next-generation sequencing experiments. While containing the largest amount of validated MTIs, the miRTarBase provides the most updated collection by comparing with other similar, previously developed databases.	miRNA IDs (data_2642)	Gene ID (data_2295)

Table 4: The free text tools that were exported automatically by the framework for the second clinical sentence.

Free Text Tools	
231	1. Arabidopsis thaliana Microarray Analysis<br>2. KEGG pathways common to both QTL and microarray based investigations<br>3. MinePath<br>4. HUMAN Microarray CEL file to candidate pathways<br>5. From cDNA Microarray Raw Data to Pathways and Published Abstracts<br>6. miRNApath

	7. MitoMiner API<br>8. PathNet<br>9. ExpressionView<br>10. GSVA<br>11. Pathways and Gene annotations for QTL region<br>12. Macat<br>13. Betr<br>14. GPSeq<br>15. UnigeneID to KEGG Pathways<br>16. Dexus<br>17. adSplit<br>18. DEGseq<br>19. antiProfiles<br>20. NCBI Gi to Kegg Pathway Descriptions<br>21. Xps<br>22. BaySeq<br>23. Gene To Pathways (Vistrails)<br>24. GlobalAncova<br>25. maSigPro<br>26. Drug Re-Purposing Workflow<br>27. GeneProf API<br>28. microRNA to KEGG Pathways and Abstracts<br>29. Abarray<br>30. Identification of differential genes using the LIMMA Bioconductor package within R<br>31. Pipeline Pilot<br>32. Gage<br>33. Identification of differential genes using t-tests by R<br>34. Goseq<br>35. GenomeGraphs<br>36. MeV

	37. DEDS<br>38. phenoTest<br>39. ADR-S<br>40. Rmagpie<br>41. Bgmix<br>42. Allen Brain Atlas API<br>43. maDB<br>44. Calculating frequencies of gene expression levels using microarray data in MaxD<br>45. GENE-Counter<br>46. ArrayStar<br>47. Kegg pathway diagrams<br>48. clusterProfiler<br>49. ERGO Genome Analysis and Discovery System<br>50. Ocplus<br>51. diffGeneAnalysis<br>52. SeqGSEA<br>53. Ssize<br>54. Lymphoma type prediction based on microarray data<br>55. Mfuzz<br>56. Myrna<br>57. metaArray<br>58. Entrez Gene to KEGG Pathway<br>59. SNAGEE<br>60. BBSeq<br>61. LMGene<br>62. caArray data retrieving<br>63. WebScipio API<br>64. Genefu<br>65. Lumi<br>66. Kegg DrugID

	67. MiMI/Metabolomics API<br>68. BiologicalNetworks API<br>69. Import and convert gene list<br>70. Mouse Microarray Analysis<br>71. Ffpe<br>72. CancerMutationAnalysis<br>73. ReactomePA<br>74. PADOG<br>75. GeneCoDis2 API<br>76. Pathview<br>77. coGPS<br>78. AffyRNADegradation<br>79. BioDB Hyperlink Management System API<br>80. MiRProf<br>81. geneRecommender<br>82. Import and convert gene list<br>83. Find pathways in which two genes co-occur (in WikiPathways)<br>84. EnrichNet API<br>85. Retrieve Pathways and Compound information from KEGG<br>86. BioCyc API<br>87. Get Pathway-Genes and gene description by Entrez gene id<br>88. FusionHunter<br>89. Copa<br>90. mirPath<br>91. CIPF RENATO API<br>92. MmPalateMiRNA<br>93. DeconRNASeq<br>94. H-InvDB API<br>95. DSGseq<br>96. cBio Cancer Genomics Data Server (CGDS) API

	97. GeneCruiser API<br>98. Biosemantics ACCCA API<br>99. DESeq<br>100. metabolic pathway.xml<br>101. SplicingGraphs<br>102. MMSEQ<br>103. EdgeR<br>104. Annmap<br>105. ALTER Reflective API<br>106. Match concept profiles with predefined set<br>107. ABBA<br>108. Genefilter<br>109. Mgsa<br>110. NOISeq<br>111. SeqSite<br>112. Puma<br>113. DGIdb API<br>114. Limma<br>115. GMOD API<br>116. goTools<br>117. piano<br>118. EDASeq<br>119. FragGeneScan<br>120. DAVID Bioinformatics API<br>121. Perform a search through NCBI eUtils eSearch<br>122. National Institute on Drug Abuse Drug Screening Tool API<br>123. Correlation service<br>124. G-Mo.R-Seq<br>125. test workbench demo '14<br>126. Get Gene Ids for Human

	127. Gowinda<br>128. VegaMC<br>129. Associated Region to Gene List<br>130. SIMPA96 API<br>131. Liliopsida Protein Alignment<br>132. FusionCatcher<br>133. tRNAscan<br>134. SomatiCA<br>135. gene ontology diagram.xml<br>136. oneChannelGUI<br>137. AgiMicroRna<br>138. DeFuse<br>139. ALEXA-Seq<br>140. topGO<br>141. ASC<br>142. SiLoCo<br>143. MiRanalyzer<br>144. gene subset extract<br>145. Mapping OligoNucleotides to an assembly<br>146. Gene to Pubmed<br>147. Epigenome<br>148. bioNMF API<br>149. tspair<br>150. TopHat-Fusion<br>151. Kegg API<br>152. JasparDB API<br>153. MiRCat<br>154. Oncosimulator<br>155. Pathway Commons API<br>156. pathRender

	157. How are you API<br>158. sigPathway<br>159. FastQ Screen<br>160. Promedas API<br>161. SLAP API<br>162. Ingenuity Variant Analysis<br>163. Retrieve a protein from the GPCRDB<br>164. Download pathways for external references list<br>165. get elements by pathway<br>166. Extract unique proteins from blast results<br>167. BSMAP<br>168. MapDamage<br>169. BIOBASE API<br>170. Drugle API<br>171. RmiR<br>172. VisANT API<br>173. Mapping microarray data onto metabolic pathways<br>174. Workflow for Protein Sequence Analysis<br>175. MiRDeep<br>176. GraphPAC<br>177. SSearch protein similarity search API<br>178. Breakpointer<br>179. E-miR<br>180. RxNorm Prescribable API<br>181. PALMA<br>182. FusionSeq<br>183. ObTiMA<br>184. PcProf predict physico-chemical profiles of proteins API<br>185. myHealthAccount API<br>186. ZINBA

	187. AIDSinfo API<br>188. MirTools<br>189. STEPStools API<br>190. get drugs by pathways<br>191. ZORRO<br>192. SeqBuster<br>193. DIAL<br>194. Predictionet<br>195. maCorrPlot<br>196. ChimeraScan<br>197. Write pathway to disk<br>198. Chipster<br>199. Triplex<br>200. Batman<br>201. Methpipe<br>202. Bgafun<br>203. Get pathways by external reference<br>204. INOH Pathway Database API<br>205. Retrieve Protein Sequence<br>206. Bililite API<br>207. GenomicTools<br>208. Les<br>209. segmentSeq<br>210. cPath API<br>211. iPAC<br>212. HIPAASpace API<br>213. Mirtarbase<br>214. NPS<br>215. Simplify a BLAST text file<br>216. LVSmiRNA

	217. Pillbox API<br>218. lifeIMAGE API<br>219. MANOR<br>220. NucleR<br>221. Download pathways for external references list (Taverna 2)<br>222. Qseq<br>223. Fetch PDB flatfile from RCSB server<br>224. SequenceVariantAnalyzer<br>225. mBPCR<br>226. test30<br>227. MetNet API<br>228. EBI CENSOR<br>229. methyAnalysis<br>230. ChEMBL API<br>231. ExiMiR

Table 5: The specific semantic categories of named entities

**Abbreviation**	**URI**	**Semantic Category**
aggp	http://linkedlifedata.com/resource/umls-semnetwork/T100	AGE
amas	http://linkedlifedata.com/resource/calbc-group/GENE	MOLECULAR_SEQUENCE
bacs	http://linkedlifedata.com/resource/calbc-group/GENE	MOLECULAR_SEQUENCE

bact	http://linkedlifedata.com/resource/calbc-group/GENE	MOLECULAR_SEQUENCE
biof	http://linkedlifedata.com/resource/calbc-group/GENE	MOLECULAR_SEQUENCE
enzy	http://linkedlifedata.com/resource/calbc-group/GENE	MOLECULAR_SEQUENCE
genf	http://linkedlifedata.com/resource/calbc-group/GENE	MOLECULAR_SEQUENCE
mbrt	http://linkedlifedata.com/resource/calbc-group/GENE	MOLECULAR_SEQUENCE
moft	http://linkedlifedata.com/resource/calbc-group/GENE	MOLECULAR_SEQUENCE
mosq	http://linkedlifedata.com/resource/calbc-group/GENE	MOLECULAR_SEQUENCE
bmod	http://linkedlifedata.com/resource/umls-semnetwork/T091	BIOMEDICAL
bodm	http://linkedlifedata.com/resource/umls-semnetwork/T091	BIOMEDICAL
anab	http://linkedlifedata.com/resource/calbc-group/ANAT	BODY_PART
anst	http://linkedlifedata.com/resource/calbc-group/ANAT	BODY_PART
bdsu	http://linkedlifedata.com/resource/calbc-group/ANAT	BODY_PART
bdsy	http://linkedlifedata.com/resource/calbc-group/ANAT	BODY_PART
blor	http://linkedlifedata.com/resource/calbc-group/ANAT	BODY_PART
bpoc	http://linkedlifedata.com/resource/calbc-group/ANAT	BODY_PART

bsoj	http://linkedlifedata.com/resource/calbc-group/ANAT	BODY_PART
ortf	http://linkedlifedata.com/resource/calbc-group/ANAT	BODY_PART
celc	http://linkedlifedata.com/resource/umls-semnetwork/T025	CELL
celf	http://linkedlifedata.com/resource/umls-semnetwork/T025	CELL
cell	http://linkedlifedata.com/resource/umls-semnetwork/T025	CELL
comd	http://linkedlifedata.com/resource/umls-semnetwork/T025	CELL
clna	http://linkedlifedata.com/resource/umls-semnetwork/T201	CLINICAL_ATTRIBUTE
medd	http://linkedlifedata.com/resource/umls-semnetwork/T074	DEVICE
diap	http://linkedlifedata.com/resource/umls-semnetwork/T060	DIAGNOSIS
dsyn	http://linkedlifedata.com/resource/umls-semnetwork/T047	DISEASE
clnd	http://linkedlifedata.com/resource/umls-semnetwork/T200	DRUG
antb	http://linkedlifedata.com/resource/umls-semnetwork/T200	DRUG
food	http://linkedlifedata.com/resource/umls-semnetwork/T168	FOOD
gngm	http://linkedlifedata.com/resource/umls-semnetwork/T028	GENE
inpo	http://linkedlifedata.com/resource/umls-semnetwork/T037	INJURY_or_POISONING

lbpr	http://linkedlifedata.com/resource/umls-semnetwork/T034	LABORATORY
lbtr	http://linkedlifedata.com/resource/umls-semnetwork/T034	LABORATORY
podg	http://linkedlifedata.com/resource/umls-semnetwork/T101	PATIENT
nnon	http://linkedlifedata.com/resource/calbc-group/CHEM	DRUG
nusq	http://linkedlifedata.com/resource/calbc-group/GENE	MOLECULAR_SEQUENCE
aapp	http://linkedlifedata.com/resource/calbc-group/CHEM	DRUG
carb	http://linkedlifedata.com/resource/calbc-group/CHEM	DRUG
crbs	http://linkedlifedata.com/resource/calbc-group/GENE	MOLECULAR_SEQUENCE
sosy	http://linkedlifedata.com/resource/umls-semnetwork/T184	SYMPTOM
virs	http://linkedlifedata.com/resource/umls-semnetwork/T005	VIRUS
vita	http://linkedlifedata.com/resource/umls-semnetwork/T127	VITAMIN
neop	http://linkedlifedata.com/resource/umls-semnetwork/T047	DISEASE
nsba	http://linkedlifedata.com/resource/calbc-group/CHEM	DRUG
orch	http://linkedlifedata.com/resource/calbc-group/CHEM	DRUG
orga	http://linkedlifedata.com/resource/calbc-group/CHEM	DRUG

orgf	http://linkedlifedata.com/resource/calbc-group/PHYS	ORGANISM_FUNCTION
patf	http://linkedlifedata.com/resource/calbc-group/DISO	ORGANISM_FUNCTION
phsf	http://linkedlifedata.com/resource/calbc-group/PHYS	ORGANISM_FUNCTION
phsu	http://linkedlifedata.com/resource/umls-semnetwork/T200	DRUG
tisu	http://linkedlifedata.com/resource/umls-semnetwork/T024	DRUG
tmco	http://linkedlifedata.com/resource/umls-semnetwork/T079	TEMPORAL_CONCEPT
topp	http://linkedlifedata.com/resource/umls-semnetwork/T061	MEDICAL_PROCEDURE
antb	http://linkedlifedata.com/resource/umls-semnetwork/T200	DRUG
diap	http://linkedlifedata.com/resource/umls-semnetwork/T061	MEDICAL_PROCEDURE
fndg	http://linkedlifedata.com/resource/umls-semnetwork/T033	FINDING
EDAM_data	http://edamontology.org/data	EDAM_DATA
EDAM_format	http://edamontology.org/format	EDAM_DATA
EDAM_operation	http://edamontology.org/operation	EDAM_OPERATION
EDAM_topic	http://edamontology.org/topic	EDAM_TOPIC
EDAM_identifier	http://edamontology.org/identifier	EDAM_ID

Figure 1: The results of the NLP framework for the first clinical question as shown in the web application

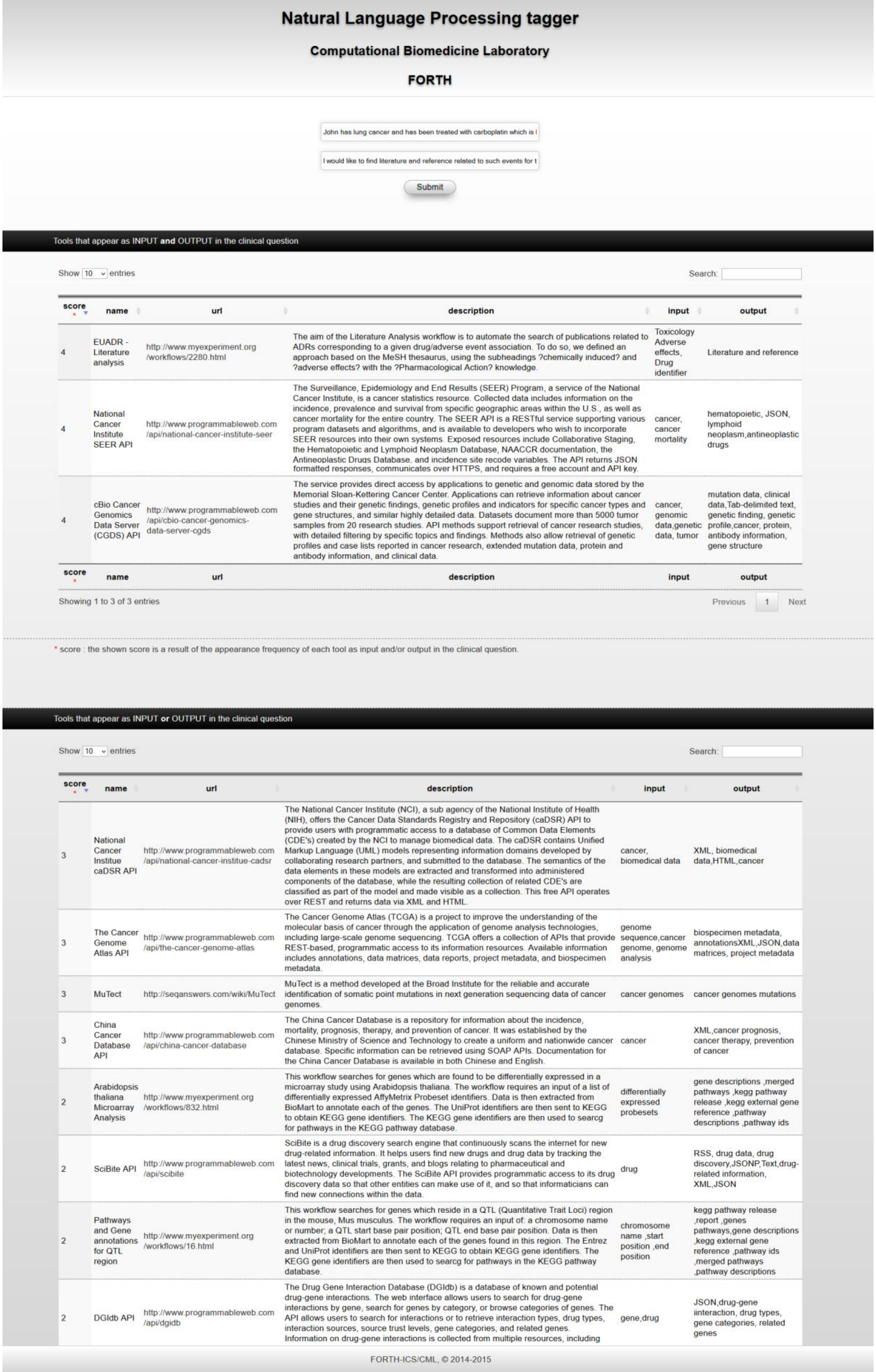

Natural Language Processing tagger

Computational Biomedicine Laboratory

FORTH

John has lung cancer and has been treated with carboplatin which is l

I would like to find literature and reference related to such events for t

Submit

Tools that appear as INPUT **and** OUTPUT in the clinical question

Show 10 entries

Search:

score *	name	url	description	input	output
4	EUADR - Literature analysis	http://www.myexperiment.org/workflows/2280.html	The aim of the Literature Analysis workflow is to automate the search of publications related to ADRs corresponding to a given drug/adverse event association. To do so, we defined an approach based on the MeSH thesaurus, using the subheadings ?chemically induced? and ?adverse effects? with the ?Pharmacological Action? knowledge.	Toxicology Adverse effects, Drug identifier	Literature and reference
4	National Cancer Institute SEER API	http://www.programmableweb.com/api/national-cancer-institute-seer	The Surveillance, Epidemiology and End Results (SEER) Program, a service of the National Cancer Institute, is a cancer statistics resource. Collected data includes information on the incidence, prevalence and survival from specific geographic areas within the U.S., as well as cancer mortality for the entire country. The SEER API is a RESTful service supporting various program datasets and algorithms, and is available to developers who wish to incorporate SEER resources into their own systems. Exposed resources include Collaborative Staging, the Hematopoietic and Lymphoid Neoplasm Database, NAACCR documentation, the Antineoplastic Drugs Database, and incidence site recode variables. The API returns JSON formatted responses, communicates over HTTPS, and requires a free account and API key.	cancer, cancer mortality	hematopoietic, JSON, lymphoid neoplasm,antineoplastic drugs
4	cBio Cancer Genomics Data Server (CGDS) API	http://www.programmableweb.com/api/cbio-cancer-genomics-data-server-cgds	The service provides direct access by applications to genetic and genomic data stored by the Memorial Sloan-Kettering Cancer Center. Applications can retrieve information about cancer studies and their genetic findings, genetic profiles and indicators for specific cancer types and gene structures, and similar highly detailed data. Datasets document more than 5000 tumor samples from 20 research studies. API methods support retrieval of cancer research studies, with detailed filtering by specific topics and findings. Methods also allow retrieval of genetic profiles and case lists reported in cancer research, extended mutation data, protein and antibody information, and clinical data.	cancer, genomic data,genetic data, tumor	mutation data, clinical data,Tab-delimited text, genetic finding, genetic profile,cancer, protein, antibody information, gene structure
score *	name	url	description	input	output

Showing 1 to 3 of 3 entries

Previous 1 Next

* score : the shown score is a result of the appearance frequency of each tool as input and/or output in the clinical question.

Tools that appear as INPUT **or** OUTPUT in the clinical question

Show 10 entries

Search:

score *	name	url	description	input	output
3	National Cancer Institue caDSR API	http://www.programmableweb.com/api/national-cancer-institue-cadsr	The National Cancer Institute (NCI), a sub agency of the National Institute of Health (NIH), offers the Cancer Data Standards Registry and Repository (caDSR) API to provide users with programmatic access to a database of Common Data Elements (CDE's) created by the NCI to manage biomedical data. The caDSR contains Unified Markup Language (UML) models representing information domains developed by collaborating research partners, and submitted to the database. The semantics of the data elements in these models are extracted and transformed into administered components of the database, while the resulting collection of related CDE's are classified as part of the model and made visible as a collection. This free API operates over REST and returns data via XML and HTML.	cancer, biomedical data	XML, biomedical data,HTML,cancer
3	The Cancer Genome Atlas API	http://www.programmableweb.com/api/the-cancer-genome-atlas	The Cancer Genome Atlas (TCGA) is a project to improve the understanding of the molecular basis of cancer through the application of genome analysis technologies, including large-scale genome sequencing. TCGA offers a collection of APIs that provide REST-based, programmatic access to its information resources. Available information includes annotations, data matrices, data reports, project metadata, and biospecimen metadata.	genome sequence,cancer genome, genome analysis	biospecimen metadata, annotationsXML,JSON,data matrices, project metadata
3	MuTect	http://seqanswers.com/wiki/MuTect	MuTect is a method developed at the Broad Institute for the reliable and accurate identification of somatic point mutations in next generation sequencing data of cancer genomes.	cancer genomes	cancer genomes mutations
3	China Cancer Database API	http://www.programmableweb.com/api/china-cancer-database	The China Cancer Database is a repository for information about the incidence, mortality, prognosis, therapy, and prevention of cancer. It was established by the Chinese Ministry of Science and Technology to create a uniform and nationwide cancer database. Specific information can be retrieved using SOAP APIs. Documentation for the China Cancer Database is available in both Chinese and English.	cancer	XML,cancer prognosis, cancer therapy, prevention of cancer
2	Arabidopsis thaliana Microarray Analysis	http://www.myexperiment.org/workflows/832.html	This workflow searches for genes which are found to be differentially expressed in a microarray study using Arabidopsis thaliana. The workflow requires an input of a list of differentially expressed AffyMetrix Probeset identifiers. Data is then extracted from BioMart to annotate each of the genes. The UniProt identifiers are then sent to KEGG to obtain KEGG gene identifiers. The KEGG gene identifiers are then used to searcg for pathways in the KEGG pathway database.	differentially expressed probesets	gene descriptions ,merged pathways ,kegg pathway release ,kegg external gene reference ,pathway descriptions ,pathway ids
2	SciBite API	http://www.programmableweb.com/api/scibite	SciBite is a drug discovery search engine that continuously scans the internet for new drug-related information. It helps users find new drugs and drug data by tracking the latest news, clinical trials, grants, and blogs relating to pharmaceutical and biotechnology developments. The SciBite API provides programmatic access to its drug discovery data so that other entities can make use of it, and so that informaticians can find new connections within the data.	drug	RSS, drug data, drug discovery,JSONP,Text,drug-related information, XML,JSON
2	Pathways and Gene annotations for QTL region	http://www.myexperiment.org/workflows/16.html	This workflow searches for genes which reside in a QTL (Quantitative Trait Loci) region in the mouse, Mus musculus. The workflow requires an input of: a chromosome name or number; a QTL start base pair position; QTL end base pair position. Data is then extracted from BioMart to annotate each of the genes found in this region. The Entrez and UniProt identifiers are then sent to KEGG to obtain KEGG gene identifiers. The KEGG gene identifiers are then used to searcg for pathways in the KEGG pathway database.	chromosome name ,start position ,end position	kegg pathway release ,report ,genes pathways,gene descriptions ,kegg external gene reference ,pathway ids ,merged pathways ,pathway descriptions
2	DGIdb API	http://www.programmableweb.com/api/dgidb	The Drug Gene Interaction Database (DGIdb) is a database of known and potential drug-gene interactions. The web interface allows users to search for drug-gene interactions by gene, search for genes by category, or browse categories of genes. The API allows users to search for interactions or to retrieve interaction types, drug types, interaction sources, source trust levels, gene categories, and related genes. Information on drug-gene interactions is collected from multiple resources, including	gene,drug	JSON,drug-gene iinteraction, drug types, gene categories, related genes

FORTH-ICS/CML, © 2014-2015
