# Supplementary material for: Semantic biomedical resource discovery: a Natural Language Processing framework
Source: BMC Med Inform Decis Mak. 2015 Sep 30;15:77. doi: 10.1186/s12911-015-0200-4 (PMC4591066; doi:10.1186/s12911-015-0200-4)
Supplement: Supplementary file 2 — contains the list of tools and resources that were in our repository. (PDF 964 kb) [file 12911_2015_200_MOESM2_ESM.pdf]

index	Name	Summary	Bio Tags (Biological application domains)	Meth Tags (Principal bioinformatics methods)	Link	Input (format)	Output (format)	Category
1	Abarray	Microarray QA and statistical data analysis for Applied Biosystems Genome Survey Microrarray (AB1700) gene expression data. Automated pipline to perform gene expression analysis for Applied Biosystems Genome Survey Microarray (AB1700) data format. Functions include data preprocessing, filtering, control probe analysis, statistical analysis in one single function. A GUI interface is also provided. The raw data, processed data, graphics output and statistical results are organized into folders according to the analysis settings used.		Microarray,OneChannel,Preprocessing,Software	http://www.bioconductor.org/packages/2.12/bioc/html/ABarray.html	Biobase,graphics,grDevices,methods,multtest,stats,tcltk,utils		Microarrays
2	ACME	Algorithms for Calculating Microarray Enrichment (ACME). ACME (Algorithms for Calculating Microarray Enrichment) is a set of tools for analysing tiling array ChIP/chip, DNAse hypersensitivity, or other experiments that result in regions of the genome showing enrichment. It does not rely on a specific array technology (although the array should be a tiling array), is very general (can be applied in experiments resulting in regions of enrichment), and is very insensitive to array noise or normalization methods. It is also very fast and can be applied on whole-genome tiling array experiments quite easily with enough memory.		Bioinformatics,Software	http://www.bioconductor.org/packages/2.12/bioc/html/ACME.html	graphics,stats		Microarrays
3	adSplit	Annotation-Driven Clustering.This package implements clustering of microarray gene expression profiles according to functional annotations. For each term genes are annotated to, splits into two subclasses are computed and a significance of the supporting gene set is determined.		Bioinformatics,Clustering,Microarray,Software	http://www.bioconductor.org/packages/2.12/bioc/html/adSplit.html	AnnotationDbi,Biobase(>= 1.5.12),cluster(>= 1.9.1),GO.db(>= 1.8.1),graphics,grDevices,KEGG.db(>= 1.8.1),methods,multtest(>= 1.6.0),stats(>= 2.1.0)		Microarrays

4	AffyRNADegradation	Analyze and correct probe positional bias in microarray data due to RNA degradation.The package helps with the assessment and correction of RNA degradation effects in Affymetrix 3' expression arrays. The parameter d gives a robust and accurate measure of RNA integrity. The correction removes the probe positional bias, and thus improves comparability of samples that are affected by RNA degradation.			http://www.bioconductor.org/packages/2.12/bioc/html/AffyRNADegradation.html			Microarrays
5	AgiMicroRna	Processing and Differential Expression Analysis of Agilent microRNA chips		AgilentChip,Bioinformatics,DifferentialExpression,Microarray,OneChannel,Preprocessing,Software	http://www.bioconductor.org/packages/release/bioc/html/AgiMicroRna.html	Biobase		Microarrays
6	annmap	Genome annotation and visualisation package pertaining to Affymetrix arrays and NGS analysis. annmap provides annotation mappings for Affymetrix exon arrays and coordinate based queries to support deep sequencing data analysis. Database access is hidden behind the API which provides a set of functions such as genesInRange(), geneToExon(), exonDetails(), etc. Functions to plot gene architecture and BAM file data are also provided. Underlying data are from Ensembl.		Annotation,Bioinformatics,Microarray,OneChannel,ReportWriting,Software,Transcription,Visualization	http://www.bioconductor.org/packages/release/bioc/html/annmap.html	DBI,RMySQL (>= 0.6-0),digest,Biobase,grid,lattice,Rsamtools,genefilter,IRanges,BiocGenerics		Microarrays
7	antiProfiles	Implementation of gene expression anti-profiles		Classification,GeneExpression,Software	http://www.bioconductor.org/packages/release/bioc/html/antiProfiles.html			Microarrays
8	apComplex	Estimate protein complex membership using AP-MS protein data. Functions to estimate a bipartite graph of protein complex membership using AP-MS data.		GraphsAndNetworks,MassSpectrometry,NetworkInference,Software	http://www.bioconductor.org/packages/release/bioc/html/apComplex.html	Rgraphviz,stats,org.Sc.sgd.db		Microarrays

9	aroma.light	Light-weight methods for normalization and visualization of microarray data using only basic R data types. Methods for microarray analysis that take basic data types such as matrices and lists of vectors. These methods can be used standalone, be utilized in other packages, or be wrapped up in higher-level classes.		Infrastructure,Microarray,MultiChannel,OneChannel,Preprocessing,Software,TwoChannel,Visualization	http://www.bioconductor.org/packages/release/bioc/html/aroma.light.html	R.methodsS3 (>= 1.4.2)		Microarrays
10	arrayQualityMetrics	Quality metrics on microarray data sets. This package generates microarray quality metrics reports for data in Bioconductor microarray data containers (ExpressionSet, NChannelSet, AffyBatch). Report contain both general and platform-specific sections. Both one and two color array platforms are supported.		Microarray,OneChannel,QualityControl,ReportWriting,Software,TwoChannel	http://www.bioconductor.org/packages/release/bioc/html/arrayQualityMetrics.html	affy,affyPLM(>= 1.27.3),beadarray,Biobase,Cairo (>= 1.4-6),genefilter,graphics,grDevices,grid,Hmisc,hwriter,lattice,latticeExtra,limma,methods,RColorBrewer,setRNG,simpleaffy,stats,SVGAnnotation (>= 0.9-0),utils,vsn(>= 3.23.3),XML		Microarrays
11	BCRANK	Predicting binding site consensus from ranked DNA sequences. Functions and classes for de novo prediction of transcription factor binding consensus by heuristic search		GeneRegulation,MotifDiscovery,Software	http://www.bioconductor.org/packages/release/bioc/html/BCRANK.html	Biostrings		Microarrays
12	betr	Identify differentially expressed genes in microarray time-course data. The betr package implements the BETR (Bayesian Estimation of Temporal Regulation) algorithm to identify differentially expressed genes in microarray time-course data.		Bioinformatics,DifferentialExpression,Microarray,Software,TimeCourse	http://www.bioconductor.org/packages/release/bioc/html/betr.html	Biobase(>= 2.5.5),limma,mvtnorm,methods,stats		Microarrays
13	bgafun	BGAfun A method to identify specifity determining residues in protein families		Bioinformatics,Classification,Software	http://www.bioconductor.org/packages/release/bioc/html/bgafun.html			Microarrays

14	Bgmix	Bayesian models for differential gene expression		DifferentialExpression,Microarray,MultipleComparisons,Software	http://www.bioconductor.org/packages/release/bioc/html/BGmix.html			Microarrays
15	biocGraph	Graph examples and use cases in Bioinformatics Bioconductor version: Release (2.12) This package provides examples and code that make use of the different graph related packages produced by Bioconductor.		GraphsAndNetworks,NetworkVisualization,Software	http://www.bioconductor.org/packages/release/bioc/html/biocGraph.html	Rgraphviz,geneplotter,graph,BiocGenerics,methods		Microarrays
16	charm	Analysis of DNA methylation data from CHARM microarrays. This package implements analysis tools for DNA methylation data generated using Nimblegen microarrays and the McrBC protocol. It finds differentially methylated regions between samples, calculates percentage methylation estimates and includes array quality assessment tools.		Bioinformatics,DNAMethylation,Microarray,Software	http://www.bioconductor.org/packages/release/bioc/html/charm.html	BSgenome,Biobase,oligo(>= 1.11.31),oligoClasses(>= 1.17.39),ff,preprocessCore,methods,stats,Biostrings,IRanges,siggenes,nor1mix,gtools,grDevices,graphics,utils,limma,parallel,sva(>= 3.1.2)		Microarrays
17	clusterProfiler	statistical analysis and visulization of functional profiles for genes and gene clusters. The package implements methods to analyze and visualize functional profiles (GO and KEGG) of gene and gene clusters.		Clustering,GO,GeneSetEnrichment,MultipleComparisons,Pathways,Software,Visualization	http://www.bioconductor.org/packages/release/bioc/html/clusterProfiler.html	methods,stats4,DBI,plyr,AnnotationDbi,GO.db,KEGG.db,org.Hs.eg.db,DOSE		Microarrays
18	clusterStab	Compute cluster stability scores for microarray data. This package can be used to estimate the number of clusters in a set of microarray data, as well as test the stability of these clusters.		Clustering,Software	http://www.bioconductor.org/packages/release/bioc/html/clusterStab.html			Microarrays

19	CMA	Synthesis of microarray-based classification. This package provides a comprehensive collection of various microarray-based classification algorithms both from Machine Learning and Statistics. Variable Selection, Hyperparameter tuning, Evaluation and Comparison can be performed combined or stepwise in a user-friendly environment.		Classification,Software	http://www.bioconductor.org/packages/release/bioc/html/CMA.html			Microarrays
20	CNAnorm	A normalization method for Copy Number Aberration in cancer samples. Performs ratio, GC content correction and normalization of data obtained using low coverage (one read every 100-10,000 bp) high troughput sequencing. It performs a discrete normalization looking for the ploidy of the genome. It will also provide tumour content if at least two ploidy states can be found.		Bioinformatics,Cancer,CopyNumberVariants,HighThroughputSequencing,Lung,Sequencing,Software	http://www.bioconductor.org/packages/release/bioc/html/CNAnorm.html	methods		Microarrays
21	codelink	Manipulation of Codelink Bioarrays data. This packages allow reading into R of Codelink bioarray data exported as text from the Codelink software. Also includes some functions to ease the manipulation and pre-processing of data, such in background correction and normalization.		DataImport,Microarray,OneChannel,Preprocessing,Software	www.bioconductor.org/packages/release/bioc/html/codelink.html	DataImport,Microarray,OneChannel,Preprocessing,Software		Microarrays
22	coGPS	cancer outlier Gene Profile Sets. Gene Set Enrichment Analysis of P-value based statistics for outlier gene detection in dataset merged from multiple studies		Bioinformatics,DifferentialExpression,Microarray,Software	http://www.bioconductor.org/packages/release/bioc/html/coGPS.html	graphics,grDevices		Microarrays
23	copa	Functions to perform cancer outlier profile analysis. COPA is a method to find genes that undergo recurrent fusion in a given cancer type by finding pairs of genes that have mutually exclusive outlier profiles.		DifferentialExpression,OneChannel,Software,TwoChannel,Visualization	http://www.bioconductor.org/packages/release/bioc/html/copa.html			Microarrays
24	daMA	Efficient design and analysis of factorial two-colour microarray data. This package contains functions for the efficient design of factorial two-colour microarray experiments and for the statistical analysis of factorial microarray data.		Bioinformatics,DifferentialExpression,Microarray,Software,TwoChannel	http://www.bioconductor.org/packages/release/bioc/html/daMA.html	MASS,stats		Microarrays

25	DeconRNASeq	Deconvolution of Heterogeneous Tissue Samples for mRNA-Seq data. DeconSeq is an R package for deconvolution of heterogeneous tissues based on mRNA-Seq data. It modeled expression levels from heterogeneous cell populations in mRNA-Seq as the weighted average of expression from different constituting cell types and predicted cell type proportions of single expression profiles.		Bioinformatics,ExperimentData,RNAExpressionData,Software	http://www.bioconductor.org/packages/release/bioc/html/DeconRNASeq.html			Microarrays
26	DEDS	Differential Expression via Distance Summary for Microarray Data. This library contains functions that calculate various statistics of differential expression for microarray data, including t statistics, fold change, F statistics, SAM, moderated t and F statistics and B statistics. It also implements a new methodology called DEDS (Differential Expression via Distance Summary), which selects differentially expressed genes by integrating and summarizing a set of statistics using a weighted distance approach.		Bioinformatics,DifferentialExpression,Microarray,Software	http://www.bioconductor.org/packages/release/bioc/html/DEDS.html			Microarrays
27	DEGseq	Identify Differentially Expressed Genes from RNA-seq data		DifferentialExpression,GeneExpression,Preprocessing,RNAseq,Software	http://www.bioconductor.org/packages/release/bioc/html/DEGseq.html	graphics,grDevices,methods,stats,utils		Microarrays

28	dexus	DEXUS - Identifying Differential Expression in RNA-Seq Studies with Unknown Conditions or without Replicates. DEXUS identifies differentially expressed genes in RNA-Seq data under all possible study designs such as studies without replicates, without sample groups, and with unknown conditions. DEXUS works also for known conditions, for example for RNA-Seq data with two or multiple conditions. RNA-Seq read count data can be provided both by the S4 class Count Data Set and by read count matrices. Differentially expressed transcripts can be visualized by heatmaps, in which unknown conditions, replicates, and samples groups are also indicated. This software is fast since the core algorithm is written in C. For very large data sets, a parallel version of DEXUS is provided in this package. DEXUS is a statistical model that is selected in a Bayesian framework by an EM algorithm. DEXUS does not need replicates to detect differentially expressed transcripts, since the replicates (or conditions) are estimated by the EM method for each transcript. The method provides an informative/non-informative value to extract differentially expressed transcripts at a desired significance level or power.		Bioinformatics,CellBiology,Classification,DifferentialExpression,GeneExpression,HapMap,HighThroughputSequencing,HighTroughputSequencingData,Homo_sapiens,Macaca_mulatta,Mus_musculus,Pan_troglodytes,QualityControl,RNAExpressionData,RNASeq,RNAseqData,Sequencing,Software,Zea_Mays	http://www.bioconductor.org/packages/release/bioc/html/dexus.html			Microarrays
29	DFP	Gene Selection. This package provides a supervised technique able to identify differentially expressed genes, based on the construction of \emph{Fuzzy Patterns} (FPs). The Fuzzy Patterns are built by means of applying 3 Membership Functions to discretized gene expression values.		Bioinformatics,DifferentialExpression,Microarray,Software	http://www.bioconductor.org/packages/release/bioc/html/DFP.html			Microarrays
30	diffGene Analysis	Performs differential gene expression Analysis. Analyze microarray data		Bioinformatics,DifferentialExpression,Microarray,Software	http://www.bioconductor.org/packages/release/bioc/html/diffGeneAnalysis.html	graphics,grDevices,minpack.lm (>= 1.0-4),stats,utils		Microarrays
31	DOSE	Disease Ontology Semantic and Enrichment analysis. Implemented five methods proposed by Resnik, Schlicker, Jiang, Lin and Wang respectively for measuring DO semantic similarities, and hypergeometric test for enrichment analysis.		Annotation,Bioinformatics,Software	http://www.bioconductor.org/packages/release/bioc/html/DOSE.html	methods,plyr,qvalue,stats4,AnnotationDbi,DO.db,org.Hs.eg.db,igraph,scales,reshape2,graphics,GOSemSim		Microarrays

32	ecolitk	Meta-data and tools for E. coli. Meta-data and tools to work with E. coli. The tools are mostly plotting functions to work with circular genomes. They can used with other genomes/plasmids.		Annotation,Software,Visualization	http://www.bioconductor.org/packages/release/bioc/html/ecolitk.html	Biobase,graphics,methods		Microarrays
33	EDASeq	Exploratory Data Analysis and Normalization for RNA-Seq. Numerical and graphical summaries of RNA-Seq read data. Within-lane normalization procedures to adjust for GC-content effect (or other gene-level effects) on read counts: loess robust local regression, global-scaling, and full-quantile normalization (Risso et al., 2011). Between-lane normalization procedures to adjust for distributional differences between lanes (e.g., sequencing depth): global-scaling and full-quantile normalization (Bullard et al., 2010).		DifferentialExpression,HighThroughputSequencing,Preprocessing,QualityControl,RNAseq,Software	http://www.bioconductor.org/packages/release/bioc/html/EDASeq.html	methods,graphics,BiocGenerics,IRanges(>= 1.13.9),DESeq		Microarrays
34	edgeR	Empirical analysis of digital gene expression data in R. Differential expression analysis of RNA-seq and digital gene expression profiles with biological replication. Uses empirical Bayes estimation and exact tests based on the negative binomial distribution. Also useful for differential signal analysis with other types of genome-scale count data.		Bioinformatics,ChIPseq,DifferentialExpression,HighThroughputSequencing,RNAseq,SAGE,Software	http://www.bioconductor.org/packages/release/bioc/html/edgeR.html			Microarrays
35	ExiMiR	R functions for the normalization of Exiqon miRNA array data. This package contains functions for reading raw data in ImaGene TXT format obtained from Exiqon miRCURY LNA arrays, annotating them with appropriate GAL files, and normalizing them using a spike-in probe-based method. Other platforms and data formats are also supported.		DualChannel,GeneExpression,Microarray,OneChannel,Preprocessing,Software,Transcription	http://www.bioconductor.org/packages/release/bioc/html/ExiMiR.html	affyio(>= 1.13.3),Biobase(>= 2.5.5),preprocessCore(>= 1.10.0)		Microarrays
36	ExpressionView	Visualize biclusters identified in gene expression data. ExpressionView visualizes possibly overlapping biclusters in a gene expression matrix. It can use the result of the ISA method (eisa package) or the algorithms in the biclust package or others. The viewer itself was developed using Adobe Flex and runs in a flash-enabled web browser.		Classification,GeneExpression,Microarray,Software,Visualization	http://www.bioconductor.org/packages/release/bioc/html/ExpressionView.html	methods,isa2,eisa,GO.db,KEGG.db,AnnotationDbi		Microarrays
37	factDesign	Factorial designed microarray experiment analysis. This package provides a set of tools for analyzing data from a factorial designed microarray experiment, or any microarray experiment for which a linear model is appropriate. The functions can be used to evaluate tests of contrast of biological interest and perform single outlier detection.		Bioinformatics,DifferentialExpression,Microarray,Software	http://www.bioconductor.org/packages/release/bioc/html/factDesign.html	stats		Microarrays

38	ffpe	Quality assessment and control for FFPE microarray expression data. Identify low-quality data using metrics developed for expression data derived from Formalin-Fixed, Paraffin-Embedded (FFPE) data. Also a function for making Concordance at the Top plots (CAT-plots).		Bioinformatics,GeneExpression,Microarray,QualityControl,Software	http://www.bioconductor.org/packages/release/bioc/html/ffpe.html	Biobase,BiocGenerics,affy,lumi,methylumi,sfsmisc		Microarrays
39	gage	Generally Applicable Gene-set Enrichment for Pathway Analysis. GAGE is a published method for gene set or pathway analysis. GAGE is generally applicable independent of microarray or RNA-Seq data attributes including sample sizes, experimental designs, assay platforms, and other types of heterogeneity, and consistently achieves superior performance over other frequently used methods. In gage package, we provide functions for basic GAGE analysis, result processing and presentation. We have also built pipeline routines for of multiple GAGE analyses in a batch, comparison between parallel analyses, and combined analysis of heterogeneous data from different sources/studies. In addition, we provide demo microarray data and commonly used gene set data based on KEGG pathways and GO terms. These funtions and data are also useful for gene set analysis using other methods.		DifferentialExpression,GO,GeneSetEnrichment,Genetics,Microarray,MultipleComparisons,OneChannel,Pathways,RNAseq,Software,TwoChannel	http://www.bioconductor.org/packages/release/bioc/html/gage.html	graph		Microarrays
40	genArise	Microarray Analysis tool. genArise is an easy to use tool for dual color microarray data. Its GUI-Tk based environment let any non-experienced user performs a basic, but not simple, data analysis just following a wizard. In addition it provides some tools for the developer.		Microarray,Preprocessing,Software,TwoChannel	http://www.bioconductor.org/packages/release/bioc/html/genArise.html	graphics,grDevices,methods,stats,tcltk,utils,xtable		Microarrays
41	genefilter	genefilter: methods for filtering genes from microarray experiments		Bioinformatics,Microarray,Software	http://www.bioconductor.org/packages/release/bioc/html/genefilter.html	AnnotationDbi,annotate(>= 1.13.7),Biobase(>= 1.99.10),graphics,methods,stats,survival		Microarrays
42	genefu	Relevant Functions for Gene Expression Analysis, Especially in Breast Cancer. Description: This package contains functions implementing various tasks usually required by gene expression analysis, especially in breast cancer studies: gene mapping between different microarray platforms, identification of molecular subtypes, implementation of published gene signatures, gene selection, survival analysis, ...		Classification,Clustering,DifferentialExpression,GeneExpression,Software,Visualization	http://www.bioconductor.org/packages/release/bioc/html/genefu.html	amap		Microarrays

43	geneRecommender	A gene recommender algorithm to identify genes coexpressed with a query set of genes. This package contains a targeted clustering algorithm for the analysis of microarray data. The algorithm can aid in the discovery of new genes with similar functions to a given list of genes already known to have closely related functions.		Clustering,Microarray,Software	http://www.bioconductor.org/packages/release/bioc/html/geneRecommender.html	Biobase,methods,stats		Microarrays
44	GenomeGraphs	Plotting genomic information from Ensembl. Genomic data analyses requires integrated visualization of known genomic information and new experimental data. GenomeGraphs uses the biomaRt package to perform live annotation queries to Ensembl and translates this to e.g. gene/transcript structures in viewports of the grid graphics package. This results in genomic information plotted together with your data. Another strength of GenomeGraphs is to plot different data types such as array CGH, gene expression, sequencing and other data, together in one plot using the same genome coordinate system.		Microarray,Software,Visualization	http://www.bioconductor.org/packages/release/bioc/html/GenomeGraphs.html			Microarrays
45	ggbio	Visualization tools for genomic data. The ggbio package extends and specializes the grammar of graphics for biological data. The graphics are designed to answer common scientific questions, in particular those often asked of high throughput genomics data. All core Bioconductor data structures are supported, where appropriate. The package supports detailed views of particular genomic regions, as well as genome-wide overviews. Supported overviews include ideograms and grand linear views. High-level plots include sequence fragment length, edge-linked interval to data view, mismatch pileup, and several splicing summaries.		Bioinformatics,Infrastructure,Software,Visualization	http://www.bioconductor.org/packages/release/bioc/html/ggbio.html	methods,biovizBase(>= 1.7.8),reshape2,gtable,ggplot2(>= 0.9.2),BiocGenerics, Biobase,IRanges,GenomicRanges,GenomicFeatures,Rsamtools,BSgenome,gridExtra,scales,plyr,VariantAnnotation,Hmisc,rtracklayer		Microarrays

46	GlobalAncova	Calculates a global test for differential gene expression between groups. We give the following arguments in support of the GlobalAncova approach: After appropriate normalisation, gene-expression-data appear rather symmetrical and outliers are no real problem, so least squares should be rather robust. ANCOVA with interaction yields saturated data modelling e.g. different means per group and gene. Covariate adjustment can help to correct for possible selection bias. Variance homogeneity and uncorrelated residuals cannot be expected. Application of ordinary least squares gives unbiased, but no longer optimal estimates (Gauss-Markov-Aitken). Therefore, using the classical F-test is inappropriate, due to correlation. The test statistic however mirrors deviations from the null hypothesis. In combination with a permutation approach, empirical significance levels can be approximated. Alternatively, an approximation yields asymptotic p-values. This work was supported by the NGFN grant 01 GR 0459, BMBF, Germany.		Bioinformatics,DifferentialExpression,Microarray,OneChannel,Pathways,Software	http://www.bioconductor.org/packages/release/bioc/html/GlobalAncova.html	annotate,AnnotationDbi		Microarrays
47	GOSemSim	GO-terms Semantic Similarity Measures. Implemented five methods proposed by Resnik, Schlicker, Jiang, Lin and Wang respectively for estimating GO semantic similarities. Support many species, including Anopheles, Arabidopsis, Bovine, Canine, Chicken, Chimp, Coelicolor, E coli strain K12 and Sakai, Fly, Human, Malaria, Mouse, Pig, Rhesus, Rat, Worm, Xenopus, Yeast, and Zebrafish.		Clustering,GO,NetworkAnalysis,Pathways,Software	www.bioconductor.org/packages/release/bioc/html/GOSemSim.html	methods,AnnotationDbi,GO.db,org.Hs.eg.db		Microarrays
48	Gostats	Tools for manipulating GO and microarrays. A set of tools for interacting with GO and microarray data. A variety of basic manipulation tools for graphs, hypothesis testing and other simple calculations.		biocViews Annotation,Bioinformatics,GO,MultipleComparisons, Software	http://www.bioconductor.org/packages/release/bioc/html/GOstats.html	AnnotationDbi(>= 0.0.89),Biobase(>= 1.15.29),Category(>= 2.3.26),GO.db(>= 1.13.0),RBGL,annotate(>= 1.13.2),graph(>= 1.15.15),methods,stats,AnnotationForge		Microarrays

49	goTools	Functions for Gene Ontology databaseBioconductor version: Release (2.12) Wraper functions for description/comparison of oligo ID list using Gene Ontology database		GO,Microarray,Software,Visualization	http://www.bioconductor.org/packages/release/bioc/html/goTools.html	AnnotationDbi,GO.db,graphics,grDevices		Microarrays
50	biomvRCNS	Copy Number study and Segmentation for multivariate biological data. In this package, a Hidden Semi Markov Model (HSMM) and one homogeneous segmentation model are designed and implemented for segmentation genomic data, with the aim of assisting in transcripts detection using high throughput technology like RNA-seq or tiling array, and copy number analysis using aCGH or sequencing.		CopyNumberVariants,Genetics,HighThroughputSequencing,Microarray,Sequencing,Software,Visualization,aCGH	http://www.bioconductor.org/packages/release/bioc/html/biomvRCNS.html	methods,mvtnorm		Microarrays
51	BioSeqClass	Classification for Biological Sequences. Extracting Features from Biological Sequences and Building Classification Model		Classification,Software	http://www.bioconductor.org/packages/release/bioc/html/BioSeqClass.html	Biostrings,ipred,e1071,klaR,randomForest,class,tree,nnet,rpart,party,foreign,Biobase,utils,stats,grDevices		Microarrays
52	biovizBase	Basic graphic utilities for visualization of genomic data. The biovizBase package is designed to provide a set of utilities, color schemes and conventions for genomic data. It serves as the base for various high-level packages for biological data visualization. This saves development effort and encourages consistency.		Bioinformatics,Infrastructure,Preprocessing,Software,Visualization	http://www.bioconductor.org/packages/release/bioc/html/biovizBase.html	methods,grDevices,stats,scales,Hmisc,RColorBrewer,dichromat,BiocGenerics,IRanges,GenomicRanges,Biostrings,Rsamtools,GenomicFeatures		Microarrays

53	CancerMutationAnalysis	Cancer mutation analysis. This package implements gene and gene-set level analysis methods for somatic mutation studies of cancer. The gene-level methods distinguish between driver genes (which play an active role in tumorigenesis) and passenger genes (which are mutated in tumor samples, but have no role in tumorigenesis) and incorporate a two-stage study design. The gene-set methods implement a patient-oriented approach, which calculates gene-set scores for each sample, then combines them across samples; a gene-oriented approach which uses the Wilcoxon test is also provided for comparison.		Bioinformatics,Genetics,Software	www.bioconductor.org/packages/release/bioc/html/CancerMutationAnalysis.html	AnnotationDbi,limma,methods,stats		Microarrays
54	GraphPAC	Identification of Mutational Clusters in Proteins via a Graph Theoretical Approach. Identifies mutational clusters of amino acids in a protein while utilizing the proteins tertiary structure via a graph theoretical model.		Bioinformatics,BiologicalDomains,Clustering,Proteomics,Software	http://www.bioconductor.org/packages/release/bioc/html/GraphPAC.html			Microarrays
55	GSVA	Gene Set Variation Analysis for microarray and RNA-seq data. Gene Set Variation Analysis (GSVA) is a non-parametric, unsupervised method for estimating variation of gene set enrichment through the samples of a expression data set. GSVA performs a change in coordinate systems, transforming the data from a gene by sample matrix to a gene-set by sample matrix, thereby allowing the evaluation of pathway enrichment for each sample. This new matrix of GSVA enrichment scores facilitates applying standard analytical methods like functional enrichment, survival analysis, clustering, CNV-pathway analysis or cross-tissue pathway analysis, in a pathway-centric manner.		GeneSetEnrichment,Microarray,Pathways,Software	http://www.bioconductor.org/packages/release/bioc/html/GSVA.html	methods,BiocGenerics,Biobase,GSEABase		Microarrays

56	Gviz	Plotting data and annotation information along genomic coordinates. Genomic data analyses requires integrated visualization of known genomic information and new experimental data. Gviz uses the biomaRt and the rtracklayer packages to perform live annotation queries to Ensembl and UCSC and translates this to e.g. gene/transcript structures in viewports of the grid graphics package. This results in genomic information plotted together with your data.		Microarray,Software,Visualization	http://www.bioconductor.org/packages/release/bioc/html/Gviz.html	IRanges(>= 1.13.19),rtracklayer(>= 1.15.5),lattice,RColorBrewer,biomaRt(>= 2.11.0),GenomicRanges(>= 1.7.14),AnnotationDbi(>= 1.17.11),Biobase(>= 2.15.3),BiocGenerics(>= 0.1.4),GenomicFeatures(>= 1.9.7),BSgenome(>= 1.25.1),Biostrings(>= 2.25.1),biovizBase(>= 1.5.7),Rsamtools(>= 1.11.1)		Microarrays
57	GWASTools	Tools for Genome Wide Association Studies. Classes for storing very large GWAS data sets and annotation, and functions for GWAS data cleaning and analysis.		GeneticVariability,Microarray,QualityControl,SNP,Software	http://www.bioconductor.org/packages/release/bioc/html/GWASTools.html	methods,DBI,RSQLite,GWASExactHW,DNAcopy,survival,lmtest,quantsmooth		Microarrays
58	Harshlight	A corrective make-up program for microarray chips. The package is used to detect extended, diffuse and compact blemishes on microarray chips. Harshlight automatically marks the areas in a collection of chips (affybatch objects) and a corrected AffyBatch object is returned, in which the defected areas are substituted with NAs or the median of the values of the same probe in the other chips in the collection. The new version handle the substitute value as whole matrix to solve the memory problem.		AffymetrixChip,Microarray,Preprocessing,QualityControl,ReportWriting,Software	http://www.bioconductor.org/packages/release/bioc/html/Harshlight.html	affy,altcdfenvs,Biobase,stats,utils		Microarrays

59	iASeq	iASeq: integrating multiple sequencing datasets for detecting allele-specific events. It fits correlation motif model to multiple RNAseq or ChIPseq studies to improve detection of allele-specific events and describe correlation patterns across studies.		Bioinformatics,ChIPseq,RNAseq,SNP,Software	http://www.bioconductor.org/packages/release/bioc/html/iASeq.html	graphics,grDevices		Microarrays
60	imageHTS	Analysis of high-throughput microscopy-based screens. imageHTS is an R package dedicated to the analysis of high-throughput microscopy-based screens. The package provides a modular and extensible framework to segment cells, extract quantitative cell features, predict cell types and browse screen data through web interfaces. Designed to operate in distributed environments, imageHTS provides a standardized access to remote data and facilitates the dissemination of high-throughput microscopy-based datasets.		CellBasedAssays,Preprocessing,Software,Visualization	http://www.bioconductor.org/packages/release/bioc/html/imageHTS.html	tools,Biobase,hwriter,methods,vsn,stats,utils,e1071		Microarrays
61	maSigPro	Significant Gene Expression Profile Differences in Time Course Microarray Data. maSigPro is a regression based approach to find genes for which there are significant gene expression profile differences between experimental groups in time course microarray experiments.		DifferentialExpression,Microarray,Software,TimeCourse	http://www.bioconductor.org/packages/release/bioc/html/maSigPro.html	Biobase,graphics,grDevices,limma,Mfuzz,stats,utils,MASS		Microarrays
62	MassArray	Analytical Tools for MassArray Data. This package is designed for the import, quality control, analysis, and visualization of methylation data generated using Sequenom's MassArray platform. The tools herein contain a highly detailed amplicon prediction for optimal assay design. Also included are quality control measures of data, such as primer dimer and bisulfite conversion efficiency estimation. Methylation data are calculated using the same algorithms contained in the EpiTyper software package. Additionally, automatic SNP-detection can be used to flag potentially confounded data from specific CG sites. Visualization includes barplots of methylation data as well as UCSC Genome Browser-compatible BED tracks. Multiple assays can be positionally combined for integrated analysis.		DNAMethylation,DataImport,Genetics,MassSpectrometry,SNP,Software,Visualization	http://www.bioconductor.org/packages/release/bioc/html/MassArray.html	graphics,grDevices,methods,stats,utils		Microarrays
63	mBPCR	Bayesian Piecewise Constant Regression for DNA copy number estimation. Estimates the DNA copy number profile using mBPCR to detect regions with copy number changes		Bioinformatics,CopyNumberVariants,Microarray,SNP,Software,aCGH	http://www.bioconductor.org/packages/release/bioc/html/mBPCR.html	Biobase		Microarrays

64	mcaGUI	Microbial Community Analysis GUI. Microbial community analysis GUI for R using gWidgets.		Bioinformatics,Clustering,GUI,Sequencing,Software,Visualization	http://www.bioconductor.org/packages/release/bioc/html/mcaGUI.html				Microarrays
65	metaArray	Integration of Microarray Data for Meta-analysis. 1) Data transformation for meta-analysis of microarray Data: Transformation of gene expression data to signed probability scale (MCMC/EM methods) 2) Combined differential expression on raw scale: Weighted Z-score after stabilizing mean-variance relation within platform.		Bioinformatics,DifferentialExpression,Microarray,Software	http://www.bioconductor.org/packages/release/bioc/html/metaArray.html	Biobase,MergeMaid,graphics,stats			Microarrays
66	methVisual	Methods for visualization and statistics on DNA methylation data. The package 'methVisual' allows the visualization of DNA methylation data after bisulfite sequencing.		Bioinformatics,Classification,Clustering,DNAMethylation,Software	http://www.bioconductor.org/packages/release/bioc/html/methVisual.html	Biostrings,ca,graphics,grDevices,grid,gridBase,IRanges,stats,utils			Microarrays
67	methyAnalysis	DNA methylation data analysis and visualization. The methyAnalysis package aims for the DNA methylation data analysis and visualization. A new class is defined to keep the chromosome location information together with the data. The current version of the package mainly focus on analyzing the Illumina Infinium methylation array data, but most methods can be generalized to other methylation array or sequencing data.		DNAMethylation,Microarray,Software,Visualization	http://www.bioconductor.org/packages/release/bioc/html/methyAnalysis.html	lumi,methylumi,Gviz,genoset,GenomicRanges,IRanges,rtracklayer,GenomicFeatures,annotate,Biobase(>= 2.5.5),AnnotationDbi,genefilter,biomaRt,methods			Microarrays
68	Mfuzz	Soft clustering of time series gene expression data. Package for noise-robust soft clustering of gene expression time-series data (including a graphical user interface)		Clustering,Microarray,Preprocessing,Software,TimeCourse,Visualization	http://www.bioconductor.org/packages/release/bioc/html/Mfuzz.html	tcltk,tkWidgets			Microarrays
69	mgsa	Model-based gene set analysis. Model-based Gene Set Analysis (MGSA) is a Bayesian modeling approach for gene set enrichment. The package mgsa implements MGSA and tools to use MGSA together with the Gene Ontology.		GO,GeneSetEnrichment,Pathways,Software	http://www.bioconductor.org/packages/release/bioc/html/mgsa.html	graphics,stats,utils			Microarrays

70	microRNA	Data and functions for dealing with microRNAs. Different data resources for microRNAs and some functions for manipulating them.		Infrastructure,SequenceAnnotation,SequenceMatching,Software	http://www.bioconductor.org/packages/release/bioc/html/microRNA.html	Biostrings(>= 2.11.32)		Microarrays
71	miRNApath	miRNApath: Pathway Enrichment for miRNA Expression Data. This package provides pathway enrichment techniques for miRNA expression data. Specifically, the set of methods handles the many-to-many relationship between miRNAs and the multiple genes they are predicted to target (and thus affect.) It also handles the gene-to-pathway relationships separately. Both steps are designed to preserve the additive effects of miRNAs on genes, many miRNAs affecting one gene, one miRNA affecting multiple genes, or many miRNAs affecting many genes.		Annotation,DifferentialExpression,NetworkEnrichment,Pathways,Software,miRNA	http://www.bioconductor.org/packages/release/bioc/html/miRNApath.html			Microarrays
72	MmPalateMiRNA	Murine Palate miRNA Expression Analysis. R package compendium for the analysis of murine palate miRNA two-color expression data.		Bioinformatics,Clustering,DifferentialExpression,GO,Microarray,MultipleComparisons,Pathways,Preprocessing,QualityControl,ReportWriting,SequenceMatching,Software,TwoChannel	http://www.bioconductor.org/packages/release/bioc/html/MmPalateMiRNA.html	limma,lattice,Biobase		Microarrays
73	motifStack	Plot stacked logos for single or multiple DNA, RNA and amino acid sequence. The motifStack package is designed for graphic representation of multiple motifs with different similarity scores. It works with both DNA/RNA sequence motif and amino acid sequence motif. In addition, it provides the flexibility for users to customize the graphic parameters such as the font type and symbol colors.		GenomicsSequence,SequenceMatching,Software,Visualization	http://www.bioconductor.org/packages/release/bioc/html/motifStack.html	grImport,grid,XML,ade4		Microarrays

74	nnNorm	Spatial and intensity based normalization of cDNA microarray data based on robust neural nets. This package allows to detect and correct for spatial and intensity biases with two-channel microarray data. The normalization method implemented in this package is based on robust neural networks fitting.		Microarray,Preprocessing,Software,TwoChannel	http://www.bioconductor.org/packages/release/bioc/html/nnNorm.html	graphics,grDevices,marray,methods,nnet,stats		Microarrays
75	NOISeq	Exploratory analysis and differential expression for RNA-seq data. Analysis of RNA-seq expression data or other similar kind of data. Exploratory plots to evualuate saturation, count distribution, expression per chromosome, type of detected features, features length, etc. Differential expression between two experimental conditions with no parametric assumptions.		Bioinformatics,DifferentialExpression,HighThroughputSequencing,RNAseq,Software,Visualization	http://www.bioconductor.org/packages/release/bioc/html/NOISeq.html			Microarrays
76	NuPoP	An R package for nucleosome positioning prediction. NuPoP is an R package for Nucleosome Positioning Prediction.		Classification,Genetics,Software,Visualization	http://www.bioconductor.org/packages/release/bioc/html/NuPoP.html			Microarrays
77	Ocplus	Operating characteristics plus sample size and local fdr for microarray experiments. This package allows to characterize the operating characteristics of a microarray experiment, i.e. the trade-off between false discovery rate and the power to detect truly regulated genes. The package includes tools both for planned experiments (for sample size assessment) and for already collected data (identification of differentially expressed genes).		Bioinformatics,DifferentialExpression,Microarray,MultipleComparisons,Software	http://www.bioconductor.org/packages/release/bioc/html/Ocplus.html	multtest(>= 1.7.3),graphics,grDevices,stats		Microarrays

78	oligo	Preprocessing tools for oligonucleotide arrays. A package to analyze oligonucleotide arrays (expression/SNP/tiling/exon) at probe-level. It currently supports Affymetrix (CEL files) and NimbleGen arrays (XYS files).		Bioinformatics,DataImport,DifferentialExpression,ExonArray,GeneExpression,Microarray,OneChannel,Preprocessing,SNP,Software,TwoChannel	http://www.bioconductor.org/packages/release/bioc/html/oligo.html	affyio(>= 1.25.0),affxparser(>= 1.29.11),Biostrings(>= 2.25.12),BiocGenerics(>= 0.3.2),DBI (>= 0.2-5),ff,graphics,methods,preprocessCore(>= 1.19.0),splines,stats,stats4,utils,zlibbioc		Microarrays
79	OLIN	Optimized local intensity-dependent normalisation of two-color microarrays. Functions for normalisation of two-color microarrays by optimised local regression and for detection of artefacts in microarray data		Microarray,Preprocessing,QualityControl,Software,TwoChannel,Visualization	http://www.bioconductor.org/packages/release/bioc/html/OLIN.html	graphics,grDevices,limma,marray,methods,stats		Microarrays
80	oneChannelGUI	A graphical interface designed to facilitate analysis of microarrays and miRNA/RNA-seq data on laptops. This package was developed to simplify the use of Bioconductor tools for beginners having limited or no experience in writing R code. This library provides a graphical interface for microarray gene and exon level analysis as well as miRNA/mRNA-seq data analysis.		DataImport,DifferentialExpression,GUI,HighThroughputSequencing,Microarray,MultipleComparisons,OneChannel,Preprocessing,QualityControl,RNAseq,Software,Statistics	http://www.bioconductor.org/packages/release/bioc/html/oneChannelGUI.html			Microarrays

81	PADOG	Pathway Analysis with Down-weighting of Overlapping Genes (PADOG). This package implements a general purpose gene set analysis method called PADOG that downplays the importance of genes that apear often accross the sets of genes to be analyzed. The package provides also a benchmark for gene set analysis methods in terms of sensitivity and ranking using 24 public datasets from KEGGdzPathwaysGEO package.		Bioinformatics,Microarray,OneChannel,Software,TwoChannel	http://www.bioconductor.org/packages/release/bioc/html/PADOG.html	graphics,limma,hgu133plus2.db,hgu133a.db,KEGG.db,AnnotationDbi,Biobase,methods,nlme		Microarrays
82	PathNet	An R package for pathway analysis using topological information. PathNet uses topological information present in pathways and differential expression levels of genes (obtained from microarray experiment) to identify pathways that are 1) significantly enriched and 2) associated with each other in the context of differential expression. The algorithm is described in: PathNet: A tool for pathway analysis using topological information.		DifferentialExpression,MultipleComparisons,Pathways,Software	http://www.bioconductor.org/packages/release/bioc/html/PathNet.html			Microarrays
83	pathRender	Render molecular pathways. build graphs from pathway databases, render them by Rgraphviz		GraphsAndNetworks,NetworkVisualization,Pathways,Software	http://www.bioconductor.org/packages/release/bioc/html/pathRender.html			Microarrays
84	pathview	a tool set for pathway based data integration and visualization. Pathview is a tool set for pathway based data integration and visualization. It maps and renders a wide variety of biological data on relevant pathway graphs. All users need is to supply their data and specify the target pathway. Pathview automatically downloads the pathway graph data, parses the data file, maps user data to the pathway, and render pathway graph with the mapped data. In addition, Pathview also seamlessly integrates with pathway and gene set analysis tools for large-scale and fully automated analysis.		Bioinformatics,DifferentialExpression,GeneExpression,GeneSetEnrichment,Genetics,GraphsAndNetworks,Metabolomics,Microarray,NetworkVisualization,Pathways,Proteomics,RNAseq,Software	http://www.bioconductor.org/packages/release/bioc/html/pathview.html	Rgraphviz,graph,png,AnnotationDbi,methods,utils		Microarrays

85	pcaGoPromoter	pcaGoPromoter is used to analyze DNA micro array data. This package contains functions to ease the analyses of DNA micro arrays. It utilizes principal component analysis as the initial multivariate analysis, followed by functional interpretation of the principal component dimensions with overrepresentation analysis for GO terms and regulatory interpretations using overrepresentation analysis of predicted transcription factor binding sites with the primo algorithm.		GO,GeneExpression,Microarray,Software,Visualization	http://www.bioconductor.org/packages/release/bioc/html/pcaGoPromoter.html	Biobase(>= 2.10.0),AnnotationDbi			Microarrays
86	phenoTest	Tools to test association between gene expression and phenotype in a way that is efficient, structured, fast and scalable. We also provide tools to do GSEA (Gene set enrichment analysis) and copy number variation. Tools to test correlation between gene expression and phenotype in a way that is efficient, structured, fast and scalable. GSEA is also provided.		Bioinformatics,Classification,Clustering,DifferentialExpression,Microarray,MultipleComparisons,Software	http://www.bioconductor.org/packages/release/bioc/html/phenoTest.html	survival,limma,Hmisc,gplots,Category,AnnotationDbi,hopach,biomaRt,GSEABase,genefilter,xtable,annotate,mgcv,SNPchip,hgu133a.db,HTSanalyzeR			Microarrays
87	piano	Platform for integrative analysis of omics data. Piano performs gene set analysis using various statistical methods, from different gene level statistics and a wide range of gene-set collections. Furthermore, the Piano package contains functions for combining the results of multiple runs of gene set analyses.		DataImport,DataRepresentation,DifferentialExpression,Microarray,Preprocessing,QualityControl,Software,Visualization	http://www.bioconductor.org/packages/release/bioc/html/piano.html	Biobase,gplots,igraph,relations,marray			Microarrays
88	predictionet	Inference for predictive networks designed for (but not limited to) genomic data. This package contains a set of functions related to network inference combining genomic data and prior information extracted from biomedical literature and structured biological databases. The main function is able to generate networks using Bayesian or regression-based inference methods; while the former is limited to < 100 of variables, the latter may infer networks with hundreds of variables. Several statistics at the edge and node levels have been implemented (edge stability, predictive ability of each node, ...) in order to help the user to focus on high quality subnetworks. Ultimately, this package is used in the 'Predictive Networks' web application developed by the Dana-Farber Cancer Institute in collaboration with Entagen.		GraphsAndNetworks,NetworkInference,Software	http://www.bioconductor.org/packages/release/bioc/html/predictionet.html	penalized,RBGL,MASS			Microarrays

89	puma	Propagating Uncertainty in Microarray Analysis. Most analyses of Affymetrix GeneChip data are based on point estimates of expression levels and ignore the uncertainty of such estimates. By propagating uncertainty to downstream analyses we can improve results from microarray analyses. For the first time, the puma package makes a suite of uncertainty propagation methods available to a general audience. puma also offers improvements in terms of scope and speed of execution over previously available uncertainty propagation methods. Included are summarisation, differential expression detection, clustering and PCA methods, together with useful plotting and data manipulation functions.		Bioinformatics,Clustering,DifferentialExpression,Microarray,OneChannel,Preprocessing,Software	http://www.bioconductor.org/packages/release/bioc/html/puma.html	Biobase(>= 2.5.5),affy(>= 1.23.4),affyio			Microarrays
90	rama	Robust Analysis of MicroArrays. Robust estimation of cDNA microarray intensities with replicates. The package uses a Bayesian hierarchical model for the robust estimation. Outliers are modeled explicitly using a t-distribution, and the model also addresses classical issues such as design effects, normalization, transformation, and nonconstant variance.		Microarray,Preprocessing,QualityControl,Software,TwoChannel	http://www.bioconductor.org/packages/release/bioc/html/rama.html				Microarrays
91	rbsurv	Robust likelihood-based survival modeling with microarray data		Bioinformatics,Microarray,Software	http://www.bioconductor.org/packages/release/bioc/html/rbsurv.html				Microarrays
92	ReactomePA	Reactome Pathway Analysis. This package provides functions for pathway analysis based on REACTOME pathway database. It will implement enrichment analysis, gene set enrichment analysis and functional modules detection.		Bioinformatics,Pathways,Software,Visualization	http://www.bioconductor.org/packages/release/bioc/html/ReactomePA.html	methods,AnnotationDbi,reactome.db,org.Hs.eg.db,stats4,plyr,igraph,qvalue,graphics			Microarrays
93	Rmagpie	MicroArray Gene-expression-based Program In Error rate estimation. Microarray Classification is designed for both biologists and statisticians. It offers the ability to train a classifier on a labelled microarray dataset and to then use that classifier to predict the class of new observations. A range of modern classifiers are available, including support vector machines (SVMs), nearest shrunken centroids (NSCs)... Advanced methods are provided to estimate the predictive error rate and to report the subset of genes which appear essential in discriminating between classes.		Classification,Microarray,Software	http://www.bioconductor.org/packages/release/bioc/html/Rmagpie.html	Biobase(>= 2.5.5),e1071,graphics,grDevices,kernlab,methods,pamr,stats,utils			Microarrays

94	RmiR	Package to work with miRNAs and miRNA targets with R. Useful functions to merge microRNA and respective targets using differents databases		GeneExpression, Microarray,Software,TimeCourse,Visualization	http://www.bioconductor.org/packages/release/bioc/html/RmiR.html	DBI,methods,stats		Microarrays
95	rtracklayer	R interface to genome browsers and their annotation tracks. Extensible framework for interacting with multiple genome browsers (currently UCSC built-in) and manipulating annotation tracks in various formats (currently GFF, BED, bedGraph, BED15, WIG, BigWig and 2bit built-in). The user may export/import tracks to/from the supported browsers, as well as query and modify the browser state, such as the current viewport.		Annotation,DataImport,Software, Visualization	http://www.bioconductor.org/packages/release/bioc/html/rtracklayer.html	XML (>= 1.98-0),BiocGenerics(>= 0.1.0),IRanges(>= 1.15.37),GenomicRanges,Biostrings(>= 2.25.6),BSgenome(>= 1.23.1),zlibbioc,RCurl (>= 1.4-2),Rsamtools(>= 1.7.3)		Microarrays
96	segmentSeq	Methods for identifying small RNA loci from high-throughput sequencing data. High-throughput sequencing technologies allow the production of large volumes of short sequences, which can be aligned to the genome to create a set of matches to the genome. By looking for regions of the genome which to which there are high densities of matches, we can infer a segmentation of the genome into regions of biological significance. The methods in this package allow the simultaneous segmentation of data from multiple samples, taking into account replicate data, in order to create a consensus segmentation. This has obvious applications in a number of classes of sequencing experiments, particularly in the discovery of small RNA loci and novel mRNA transcriptome discovery.		Bioinformatics,HighThroughputSequencing,MultipleComparisons,Software	http://www.bioconductor.org/packages/release/bioc/html/segmentSeq.html	baySeq,graphics,grDevices,IRanges,methods,utils,GenomicRanges		Microarrays
97	SeqArray	Big Data Management of Genome-wide Sequencing Variants. Big data management of genome-wide variants using the CoreArray library, where genotypic data and annotations are stored in an array-oriented manner, offering efficient access of genetic variants using the R language.		Bioinformatics,Infrastructure,Software	http://www.bioconductor.org/packages/release/bioc/html/SeqArray.html			Microarrays

98	sigPathway	Pathway Analysis. Conducts pathway analysis by calculating the NT_k and NE_k statistics		Bioinformatics,DifferentialExpression,MultipleComparisons,Software	http://www.bioconductor.org/packages/release/bioc/html/sigPathway.html			Microarrays
99	SIM	Integrated Analysis on two human genomic datasets. Finds associations between two human genomic datasets.		Bioinformatics,Microarray,Software,Visualization	http://www.bioconductor.org/packages/release/bioc/html/SIM.html	graphics,stats,globaltest,quantsmooth		Microarrays
100	sizepower	Sample Size and Power Calculation in Micorarray Studies. This package has been prepared to assist users in computing either a sample size or power value for a microarray experimental study. The user is referred to the cited references for technical background on the methodology underpinning these calculations. This package provides support for five types of sample size and power calculations. These five types can be adapted in various ways to encompass many of the standard designs encountered in practice.		Bioinformatics,Microarray,Software	http://www.bioconductor.org/packages/release/bioc/html/sizepower.html			Microarrays
101	SLGI	Synthetic Lethal Genetic Interaction. A variety of data files and functions for the analysis of genetic interactions		Genetics,GraphsAndNetworks,NetworkAnalysis,Proteomics,Software	http://www.bioconductor.org/packages/release/bioc/html/SLGI.html	AnnotationDbi,Biobase,GO.db,ScISI,graphics,lattice,methods,stats,BiocGenerics		Microarrays
102	SNAGEE	Signal-to-Noise applied to Gene Expression Experiments. Signal-to-Noise applied to Gene Expression Experiments. Signal-to-noise ratios can be used as a proxy for quality of gene expression studies and samples. The SNRs can be calculated on any gene expression data set as long as gene IDs are available, no access to the raw data files is necessary. This allows to flag problematic studies and samples in any public data set.		Microarray,OneChannel,QualityControl,Software,TwoChannel	http://www.bioconductor.org/packages/release/bioc/html/SNAGEE.html			Microarrays

103	snm	Supervised Normalization of Microarrays. SNM is a modeling strategy especially designed for normalizing high-throughput genomic data. The underlying premise of our approach is that your data is a function of what we refer to as study-specific variables. These variables are either biological variables that represent the target of the statistical analysis, or adjustment variables that represent factors arising from the experimental or biological setting the data is drawn from. The SNM approach aims to simultaneously model all study-specific variables in order to more accurately characterize the biological or clinical variables of interest.		DifferentialExpression,ExonArray,GeneExpression,Microarray,MultiChannel,MultipleComparisons,OneChannel,Preprocessing,QualityControl,Software,Transcription,TwoChannel	http://www.bioconductor.org/packages/release/bioc/html/snm.html			Microarrays
104	SomatiCA	SomatiCA: identifying, characterizing, and quantifying somatic copy number aberrations from cancer genome sequencing. SomatiCA is a software suite that is capable of identifying, characterizing, and quantifying somatic CNAs from cancer genome sequencing. First, it uses read depths and lesser allele frequencies (LAF) from mapped short sequence reads to segment the genome and identify candidate CNAs. Second, SomatiCA estimates the admixture rate from the relative copy-number profile of tumor-normal pair by a Bayesian finite mixture model. Third, SomatiCA quantifies absolute somatic copy-number and subclonality for each genomic segment to guide its characterization. Results from SomatiCA can be further integrated with single nucleotide variations (SNVs) to get a better understanding of the tumor evolution.		Bioinformatics,CopyNumberVariants,Sequencing,Software	http://www.bioconductor.org/packages/release/bioc/html/SomatiCA.html	foreach,lars,sn,DNAcopy,methods,rebmix,GenomicRanges,Iranges		Microarrays
105	SplicingGraphs	Create, manipulate, visualize splicing graphs, and assign RNA-seq reads to them. This package allows the user to create, manipulate, and visualize splicing graphs and their bubbles based on a gene model for a given organism. Additionally it allows the user to assign RNA-seq reads to the edges of a set of splicing graphs, and to summarize them in different ways.		Annotation,DataRepresentation,GeneExpression,Genetics,RNAseq,Sequencing,Software,Visualization	http://www.bioconductor.org/packages/release/bioc/html/SplicingGraphs.html	methods,utils,igraph,BiocGenerics,IRanges,GenomicRanges,GenomicFeatures,graph,Rgraphviz		Microarrays
106	ssize	Estimate Microarray Sample Size. Functions for computing and displaying sample size information for gene expression arrays.		Bioinformatics,DifferentialExpression,Microarray,Software	http://www.bioconductor.org/packages/release/bioc/html/ssize.html			Microarrays

107	stepNorm	Stepwise normalization functions for cDNA microarrays		Microarray,Preprocessing,Software,TwoChannel	http://www.bioconductor.org/packages/release/bioc/html/stepNorm.html	marray,MASS,methods,stats		Microarrays
108	topGO	topGO: Enrichment analysis for Gene Ontology. topGO package provides tools for testing GO terms while accounting for the topology of the GO graph. Different test statistics and different methods for eliminating local similarities and dependencies between GO terms can be implemented and applied.		Bioinformatics,Microarray,Software,Visualization	http://www.bioconductor.org/packages/release/bioc/html/topGO.html	methods,graph,Biobase,SparseM,AnnotationDbi,lattice		Microarrays
109	triplex	Search and visualize intramolecular triplex-forming sequences in DNA. This package provides functions for identification and visualization of potential intramolecular triplex patterns in DNA sequence. The main functionality is to detect the positions of subsequences capable of folding into an intramolecular triplex (H-DNA) in a much larger sequence. The potential H-DNA (triplexes) should be made of as many cannonical nucleotide triplets as possible. The package includes visualization showing the exact base-pairing in 1D, 2D or 3D.		GeneRegulation,SequenceMatching,Software	http://www.bioconductor.org/packages/release/bioc/html/triplex.html	methods,grid,Biostrings,GenomicRanges		Microarrays
110	tspair	Top Scoring Pairs for Microarray Classification. These functions calculate the pair of genes that show the maximum difference in ranking between two user specified groups. This top scoring pair maximizes the average of sensitivity and specificity over all rank based classifiers using a pair of genes in the data set. The advantage of classifying samples based on only the relative rank of a pair of genes is (a) the classifiers are much simpler and often more interpretable than more complicated classification schemes and (b) if arrays can be classified using only a pair of genes, PCR based tests could be used for classification of samples. See the references for the tspcalc() function for references regarding TSP classifiers.		Bioinformatics,Microarray,Software	http://www.bioconductor.org/packages/release/bioc/html/tspair.html			Microarrays
111	TurboNorm	A fast scatterplot smoother suitable for microarray normalization. A fast scatterplot smoother based on B-splines with second-order difference penalty. Functions for microarray normalization of single-colour data i.e. Affymetrix/Illumina and two-colour data supplied as marray MarrayRaw-objects or limma RGList-objects are available.		CpGIsland,DNAMethylation,Microarray,OneChannel,Preprocessing,Software,TwoChannel	http://www.bioconductor.org/packages/release/bioc/html/TurboNorm.html	stats,grDevices,affy,lattice		Microarrays

112	virtualArray	Build virtual array from different microarray platforms. This package permits the user to combine raw data of different microarray platforms into one virtual array. It consists of several functions that act subsequently in a semi-automatic way. Doing as much of the data combination and letting the user concentrate on analysing the resulting virtual array.		Bioinformatics,DataImport,Microarray,MultipleComparisons,OneChannel,Preprocessing,Software	http://www.bioconductor.org/packages/release/bioc/html/virtualArray.html	affy,affyPLM,AnnotationDbi,Biobase,gcrma,GEOquery,graphics,methods,reshape2,stats,utils,tseries,outliers		Microarrays
113	vsn	Variance stabilization and calibration for microarray data. The package implements a method for normalising microarray intensities, both between colours within array, and between arrays. The method uses a robust variant of the maximum-likelihood estimator for the stochastic model of microarray data described in the references (see vignette). The model incorporates data calibration (a.k.a. normalization), a model for the dependence of the variance on the mean intensity, and a variance stabilizing data transformation. Differences between transformed intensities are analogous to normalized log-ratios. However, in contrast to the latter, their variance is independent of the mean, and they are usually more sensitive and specific in detecting differential transcription.		Microarray,OneChannel,Preprocessing,Software,TwoChannel	http://www.bioconductor.org/packages/release/bioc/html/vsn.html	methods,affy(>= 1.23.4),limma,lattice		Microarrays
114	wateRmelon	Illumina 450 methylation array normalization and metrics. 15 flavours of betas and three performance metrics, with methods for objects produced by methylumi, minfi and IMA packages.		DNAMethylation,Microarray,Preprocessing,QualityControl,Software,TwoChannel	http://www.bioconductor.org/packages/release/bioc/html/wateRmelon.html			Microarrays

115	VegaMC	VegaMC: A Package Implementing a Variational Piecewise Smooth Model for Identification of Driver Chromosomal Imbalances in Cancer. This package enables the detection of driver chromosomal imbalances including loss of heterozygosity (LOH) from array comparative genomic hybridization (aCGH) data. VegaMC performs a joint segmentation of a dataset and uses a statistical framework to distinguish between driver and passenger mutation. VegaMC has been implemented so that it can be immediately integrated with the output produced by PennCNV tool. In addition, VegaMC produces in output two web pages that allows a rapid navigation between both the detected regions and the altered genes. In the web page that summarizes the altered genes, the link to the respective Ensembl gene web page is reported.		Bioinformatics,CopyNumberVariants,Software,aCGH	http://www.bioconductor.org/packages/release/bioc/html/VegaMC.html	methods		Microarrays
116	xps	Processing and Analysis of Affymetrix Oligonucleotide Arrays including Exon Arrays, Whole Genome Arrays and Plate Arrays. The package handles pre-processing, normalization, filtering and analysis of Affymetrix GeneChip expression arrays, including exon arrays (Exon 1.0 ST: core, extended, full probesets), gene arrays (Gene 1.0 ST) and plate arrays on computers with 1 GB RAM only. It imports Affymetrix .CDF, .CLF, .PGF and .CEL as well as annotation files, and computes e.g. RMA, MAS5, FARMS, DFW, FIRMA, tRMA, MAS5-calls, DABG-calls, I/NI-calls. It is an R wrapper to XPS (eXpression Profiling System), which is based on ROOT, an object-oriented framework developed at CERN. Thus, the prior installation of ROOT is a prerequisite for the usage of this package, however, no knowledge of ROOT is required.		DataImport,DifferentialExpression,ExonArray,GeneExpression,Microarray,OneChannel,Preprocessing,Software,Transcription	http://www.bioconductor.org/packages/release/bioc/html/xps.html			Microarrays
117	maigesPack	Functions to handle cDNA microarray data, including several methods of data analysis. This package uses functions of various other packages together with other functions in a coordinated way to handle and analyse cDNA microarray data		Classification,Clustering,ConnectTools,DifferentialExpression,GraphsAndNetworks,Microarray,Preprocessing,Software,TwoChannel	http://www.bioconductor.org/packages/release/bioc/html/maigesPack.html			Microarrays

118	MANOR	CGH Micro-Array NORmalization. Importation, normalization, visualization, and quality control functions to correct identified sources of variability in array-CGH experiments.		CopyNumberVariants,DataImport,Microarray,Preprocessing,QualityControl,Software,TwoChannel	http://www.bioconductor.org/packages/release/bioc/html/MANOR.html	GLAD,graphics,grDevices,stats,utils		Microarrays
119	marray	Exploratory analysis for two-color spotted microarray data. Class definitions for two-color spotted microarray data. Fuctions for data input, diagnostic plots, normalization and quality checking.		Microarray,Preprocessing,Software,TwoChannel	http://www.bioconductor.org/packages/release/bioc/html/marray.html			Microarrays
120	impute	impute: Imputation for microarray data		Bioinformatics,Microarray,Software	http://www.bioconductor.org/packages/release/bioc/html/impute.html			Microarrays
121	iPAC	Identification of Protein Amino acid Clustering. iPAC is a novel tool to identify somatic amino acid mutation clustering within proteins while taking into account protein structure.		Bioinformatics,BiologicalDomains,Clustering,Proteomics,Software	http://www.bioconductor.org/packages/release/bioc/html/iPAC.html			Microarrays
122	isobar	Analysis and quantitation of isobarically tagged MSMS proteomics data. isobar provides methods for preprocessing, normalization, and report generation for the analysis of quantitative mass spectrometry proteomics data labeled with isobaric tags, such as iTRAQ and TMT.		Bioinformatics,MassSpectrometry,MultipleComparisons,Proteomics,QualityControl,Software	http://www.bioconductor.org/packages/release/bioc/html/isobar.html	distr		Microarrays
123	les	Identifying Differential Effects in Tiling Microarray Data. The 'les' package estimates Loci of Enhanced Significance (LES) in tiling microarray data. These are regions of regulation such as found in differential transcription, CHiP-chip, or DNA modification analysis. The package provides a universal framework suitable for identifying differential effects in tiling microarray data sets, and is independent of the underlying statistics at the level of single probes.		Bioinformatics,ChIPchip,DNAMethylation,DifferentialExpression,Microarray,Software,Transcription	http://www.bioconductor.org/packages/release/bioc/html/les.html	boot,gplots,RColorBrewer		Microarrays

124	limma	Linear Models for Microarray Data. Data analysis, linear models and differential expression for microarray data.		Bioinformatics,DataImport,DifferentialExpression,Microarray,MultipleComparisons,OneChannel,Preprocessing,QualityControl,Software,TimeCourse,TwoChannel	http://www.bioconductor.org/packages/release/bioc/html/limma.html			Microarrays
125	LMGene	LMGene Software for Data Transformation and Identification of Differentially Expressed Genes in Gene Expression Arrays		Bioinformatics,DifferentialExpression,Microarray,Preprocessing,Software	http://www.bioconductor.org/packages/release/bioc/html/LMGene.html			Microarrays
126	LPE	Methods for analyzing microarray data using Local Pooled Error (LPE) method. This LPE library is used to do significance analysis of microarray data with small number of replicates. It uses resampling based FDR adjustment, and gives less conservative results than traditional 'BH' or 'BY' procedures. Data accepted is raw data in txt format from MAS4, MAS5 or dChip. Data can also be supplied after normalization. LPE library is primarily used for analyzing data between two conditions. To use it for paired data, see LPEP library. For using LPE in multiple conditions, use HEM library.		Bioinformatics,DifferentialExpression,Microarray,Software	http://www.bioconductor.org/packages/release/bioc/html/LPE.html	stats		Microarrays

127	lumi	BeadArray Specific Methods for Illumina Methylation and Expression Microarrays. The lumi package provides an integrated solution for the Illumina microarray data analysis. It includes functions of Illumina BeadStudio (GenomeStudio) data input, quality control, BeadArray-specific variance stabilization, normalization and gene annotation at the probe level. It also includes the functions of processing Illumina methylation microarrays, especially Illumina Infinium methylation microarrays.		DNAMethylation, Microarray,OneChannel,Preprocessing,QualityControl,Software,TwoChannel	http://www.bioconductor.org/packages/release/bioc/html/lumi.html	affy(>= 1.23.4),methylumi(>= 2.3.2),annotate,Biobase(>= 2.5.5),lattice,mgcv (>= 1.4-0),hdrcde,nleqslv,KernSmooth,preprocessCore,RSQLite,DBI,AnnotationDbi,MASS,graphics,stats,stats4,methods		Microarrays
128	LVSmiRNA	LVS normalization for Agilent miRNA data		AgilentChip,Microarray,OneChannel,Preprocessing,Software	http://www.bioconductor.org/packages/release/bioc/html/LVSmiRNA.html	BiocGenerics,stats4		Microarrays
129	maanova	Tools for analyzing Micro Array experiments. Analysis of N-dye Micro Array experiment using mixed model effect. Containing analysis of variance, permutation and bootstrap, cluster and consensus tree.		Clustering,DifferentialExpression,Microarray,Software	http://www.bioconductor.org/packages/release/bioc/html/maanova.html	Biobase,graphics,grDevices,methods,stats,utils		Microarrays
130	macat	MicroArray Chromosome Analysis Tool. This library contains functions to investigate links between differential gene expression and the chromosomal localization of the genes. MACAT is motivated by the common observation of phenomena involving large chromosomal regions in tumor cells. MACAT is the implementation of a statistical approach for identifying significantly differentially expressed chromosome regions. The functions have been tested on a publicly available data set about acute lymphoblastic leukemia (Yeoh et al.Cancer Cell 2002), which is provided in the library 'stjudem'.		DifferentialExpression,Microarray,Software,Visualization	http://www.bioconductor.org/packages/release/bioc/html/macat.html			Microarrays
131	maCorrPlot	Visualize artificial correlation in microarray data. Graphically displays correlation in microarray data that is due to insufficient normalization		Microarray,Preprocessing,Software,Visualization	http://www.bioconductor.org/packages/release/bioc/html/maCorrPlot.html	graphics,grDevices,lattice,stats		Microarrays

132	maDB	Microarray database and utility functions for microarray data analysis. maDB allows to create a simple microarray database to store microarray experiments and annotation data into it. Affymetrix GeneChip expression values as well as values from two color microarrays can be stored into the database. Whole experiments or subsets from a experiment (or also values for a subset of genes in a subset of microarrays) can be fetched back to R. Additionally maDB provides different utility functions for the microarray data analysis like functions to draw MA plots or volcano plots with the data points color coded according to the local point density or functions that allow a replicate handling of miroarrays.		Microarray,OneChannel,Software,TwoChannel,Visualization	http://www.bioconductor.org/packages/release/bioc/html/maDB.html			Microarrays
133	made4	Multivariate analysis of microarray data using ADE4. Multivariate data analysis and graphical display of microarray data. Functions include between group analysis and coinertia analysis. It contains functions that require ADE4.		Bioinformatics,Classification,Clustering,MultipleComparisons,Software	http://www.bioconductor.org/packages/release/bioc/html/made4.html			Microarrays
134	ALTER Reflective API	The service provides conversion of DNA and protein alignment sequences from biological research to express the same information in alternative notation and as input for a different analytical software package. Input and output formats supported include ALN, FASTA, GDE, MSF, NEXUS, PHYLIP, and PIR. Analytical packages supported include Clustal, Mafft, Tcoffee, Muscle, and Probcons. The service can also de-dupe results to remove multiple identical repetitions from results. API methods support description of input data files, including operating system, analytical software, and data formatting, along with the same parameters for desired output. Methods allow selection of conversion options such as treatment of duplicate and error results. The API returns data converted from the input data expressed for the desired output format.	science,biology,genetics		http://www.programmableweb.com/api/alter-reflective	DNA, protein alignment sequence, ALN, FASTA, GDE, MSF, NEXUS, PHYLIP, PIR	ALN, FASTA, GDE, MSF, NEXUS, PHYLIP, PIR	Microarrays

135	BIOBASE API	BIOBASE is the leading provider of expert-curated biological databases, software and services for the life sciences. BIOBASE products are used to identify relations that aid in drug and biomarker discovery. BIOBASE helps researchers to identify connections by offering well-structured data, assembled by highly qualified subject-matter experts, organized in an accessible and easily searchable manner. This data is accessible via an API. The API uses RESTful calls and responses are formatted in XML.	biology,reference,science, research		http://www.programmableweb.com/api/biobase	drug, biomarker discovery	XML	Systems Biology
136	BioCyc API	The service provides aggregated access to almost 2000 databases of organism genome data (formally, Pathway/Genome Databases or PGDBs). Each database describes the genetic makeup and metabolic pathways of one biological organism. The service helps to navigate data, with tools for visualization of data relationships and analysis, both of individual organism data and comparisons among organisms. API methods support description of individual genes, including proteins, pathways, enzymes, reactions, and products generated. In addition to metabolic pathways and other characteristics of individual genes, methods enable the service's genome browser and metabolic maps relating separate genes.	science,biology,research		http://www.programmableweb.com/api/biocyc	genetic makeup, metabolic pathways, biological organism, genes, proteins, pathways, enzymes, reactions, products generated	navigate data, visualization of data relationship, analysis, comparison, metabolic pathways, genes, genome, metabolic maps, XML	Systems Biology
137	BioDB Hyperlink Management System API	The service maintains and automatically updates a database of hyperlinks across major biology and life sciences databases. It defines stable identifiers linking to data IDs in major databases for gene and protein information. Its update function returns up-to-date URLs for accessing corresponding data across multiple databases. API methods support submission of an identifier in a particular input format and the desired output format. Methods return the correct identifier in the requested output system. Methods also allow retrieval of a listing of identifiers logged in the system.	science,research,biology,genetics		http://www.programmableweb.com/api/biodb-hyperlink-management-system	gene, protein	JSON,plain text	Systems Biology

138	BiologicalNetworks API	BiologicalNetworks is a software platform for analyzing and querying biological pathways. It can also be used for the visualization of gene regulation and protein interaction networks, as well as metabolic and signaling pathways. The BiologicalNetworks API allows the functions of this platform to be incorporated into other software applications.	visualization, biology,science		http://www.programmableweb.com/api/biologicalnetworks	biological pathways	analyze biological pathways, gene visualization, protein interaction, metabolic pathways, signlaing pathways, XML	Systems Biology
139	Brain Maps API	Brain Maps is an online, interactive brain atlas, including both primate and non-primate brains. Brain Maps provides scanned images of sub-micron resolution brain sections. These images are annotated and a database allows for querying and retrieval of data about brain structure and function. Complete brain atlases are available for certain species. The Brain Maps API is a multiresolution image viewer with customizable label overlays. It is a free service, available for any web site that is free to consumers.	biology,reference,science		http://www.programmableweb.com/api/brain-maps	brain	brain scanned images, brain structure, brain functions	Imaging
140	cBio Cancer Genomics Data Server (CGDS) API	The service provides direct access by applications to genetic and genomic data stored by the Memorial Sloan-Kettering Cancer Center. Applications can retrieve information about cancer studies and their genetic findings, genetic profiles and indicators for specific cancer types and gene structures, and similar highly detailed data. Datasets document more than 5000 tumor samples from 20 research studies. API methods support retrieval of cancer research studies, with detailed filtering by specific topics and findings. Methods also allow retrieval of genetic profiles and case lists reported in cancer research, extended mutation data, protein and antibody information, and clinical data.	science,biology,search,genetics		http://www.programmableweb.com/api/cbio-cancer-genomics-data-server-cgds	genetic data, genomic data, cancer, tumor	cancer, genetic finding, genetic profile, gene structure,mutation data, protein, antibody information, clinical data,Tab-delimited text	Microarrays

141	ChEMBL API	The service from the European Bioinformatics Institute (EBI) provides programmatic access to a database of biologically active small molecules with drug-like effects. Data available include 2-D structure diagrams, calculated properties, and indicators of biological activities. API methods support retrieval of lists of chemical compounds based on inputs in various formats, from the local database ID to standard chemistry designations. The API can also retrieve components or substructures and similar compounds for a submitted identifier. Methods can return bioactivity measures as part of descriptive data.	science,medical,biology,research,search		http://www.programmableweb.com/api/chembl	molecules	chemical compounds, bioactivity measures, XML,JSON	Systems Biology
142	CIPF RENATO API	The service provides a RESTful interface to RENATO (REgulatory Network Analysis TOol) which provides network-based analysis to identify regulatory elements common to a list of genes. It is intended for interpretation and visualization of information about gene regulation by mapping genes submitted to the regulatory network and extracting connections. It also highlights over-representation of specific regulatory connections among genes in the list. API methods support submission of a list of genes with parameters to specify regulatory connections of interest. Methods generate data showing transcriptional and post-transcriptional regulatory connections and highlighting regulatory links common among genes within the specified list.	biology,science,genetics		http://www.programmableweb.com/api/cipf-renato	genes	gene interpretation, gene visualization, transcriptional, post-transcriptional, Text	Systems Biology
143	COILS predict protein coiled-coil regions API	The service provides predictions of coiled-coil regions within a protein structure through application of an algorithm from Lupas et al. It assesses the probability that some part of a protein structure represents a coiled-coil formation by comparing flanking sequences with those of proteins established as exhibiting such formations. API methods support submission of a protein sequence to generate a processing job and begin processing. The API returns any elements within the sequence that match known exemplars of coiled-coil structures.	science,biology,genetics		http://www.programmableweb.com/api/coils-predict-protein-coiled-coil-regions	protein, protein structure, coiled-coil formation	comparison flanking sequences, protein sequence, coiled-coil structure, XML	Systems Biology
144	Conservation Scorer API	The Conservation Scorer API calculates how much Eukaryotic Linear Motif (ELM) matches vary in a set of homologous protein sequences. A console is given for data input, but the SOAP calls are also made available for those who wish to integrate the Conservation Scorer API into other applications.	biology,science		http://www.programmableweb.com/api/conservation-scorer	homologous protein sequences	eykaryotic linear motif, homologous protein sequence, XML	Systems Biology

145	DAVID Bioinformatics API	The service provides tools for functional annotation to discover the implications for biological research of specific genes and genetic markers tracked by its database. Formally named Database for Annotation, Visualization and Integrated Discovery (DAVID), it logs descriptions of genetic combinations found by researchers to enhance understanding of likely biological effects and target research. API methods support retrieval of annotations listing biological functions for specified individual genes and gene clusters in both tabular and chart form. Methods also support review of full gene reports for wider-scale informatics applications.	science,medical,biology,research		http://www.programmableweb.com/api/david-bioinformatics	genes, genetic markers	visualization, annotaion listing biollogical functions, individual genes, gene cluster, review full gene report, HTML,Text	Systems Biology
146	DIALIGN API	DIALIGN is a program for aligning multiple protein or nucleic acid sequences. It constructs alignments by comparing entire segments of the sequences. This method can be used for both global and local alignment, but it is most successful when sequences share only local homologies. DIALIGN is available as a download, a web console, and a SOAP API.	biology,science		http://www.programmableweb.com/api/dialign	protein, sequences with local homologies	protein alignment, nucleid acid sequences, XML	Systems Biology
147	Divide-and-Conquer Multiple Sequence Alignment API	Divide-and-Conquer Multiple Sequence Alignment (DCA) is a program for producing fast, high quality, simultaneous multiple alignments of amino acid, RNA, or DNA sequences. The program is based on an algorithm that provides a heuristic approach to sum-of-pairs optimal alignment.DCA is available as a download, a web console, and a SOAP API.	genetics,science,biology		http://www.programmableweb.com/api/divide-and-conquer-multiple-sequence-alignment	RNA, DNA, amino acid	amino acid alignment, RNA sequences, DNA sequences, XML	Systems Biology

148	EnrichNet API	The service provides analysis of expected functional associations between genes and proteins submitted as a list. It also generates cellular processes, pathways, and complexes associated with the listed genes and proteins. Analysis results provide statistical rating of the importance of overlapping functional characteristics between lists of genes or proteins. API methods support submission of one or more genes or proteins specified by standard protocols (ensembl, HGNC symbol, etc.) along with the database to provide functional characteristics for the analysis. For larger batch analysis, methods allow uploads of files with tab-delimited values specifying the genes/proteins and interactions to be analyzed.	science,research,biology		http://www.programmableweb.com/api/enrichnet	genes, proteins, gene interaction, protein interaction	gene and protein association, cellular processes, pathways complexes of genes and proteins, analysis,Text	Systems Biology
149	Epidemic Marketplace API	The service promotes collaboration among researchers in epidemiology by compiling data sets and related resources and making them available for reuse. The site was developed as part of a multidisciplinary European research effort to build tools for forecasting epidemic and public health events. Researchers can upload their own results and download data contributed by others. They can also browse and review background reports and related resources. API methods support search for specific data objects or for collections of data objects related to a topic. Methods provide retrieval of data objects discovered or construction of tree structures depicting the relationships of documented research. Methods also allow upload, updating, and deleting of a researcher's own data sets.	science,medical,biology,research,deadpool		http://www.programmableweb.com/api/epidemic-marketplace	data sets	forecast epidemic events, relationship of documented research, XML	Systems Biology
150	FastA protein similarity search API	The service compares a protein sequence submitted with a request against a database of protein structures. It reports matches with known protein sequences, indicating genetic relationships between the sample under analysis and sequences already documented and supporting conclusions about the sample's biological heritage. API methods support submission of a protein sequence to trigger analysis based on the FastA algorithm along with the specific reference database for the comparison. The API returns matching sequences found in the selected database to allow further analysis of genetic relationships with known proteins structures.	science,biology,genetics		http://www.programmableweb.com/api/fasta-protein-similarity-search	protein sequence, protein structure	protein sequences, genetic relationship, comparison protein sequences, protein structures, XML	Microarrays

151	GeneCo Dis2 API	The service provides analysis of genetic sequence data to identify those associated with biological features, known as annotations, that frequently appear together. It then ranks these sets of genes based on the statistical significance of the frequency of co-occurrence. The results indicate the strength of association between biological annotations and a research project's identified set of genes. API methods support analysis of statistical relationships between genetic sequences based on multiple repositories of data documenting statistical relationships. A request can specify the organism genome to be the focus of analysis along with the statistical algorithm and method to apply plus the gene sequence and set of biological annotations to consider. Results suggest statistical associations between those annotations within the specified sequences for the specified organism.	genetics,biology,science		http://www.programmableweb.com/api/genecodis2	genetic sequence, biologcal features, genes	identify set of genes, analysis of genetic sequences relationships, organism genomeXML	Microarrays
152	GeneProf API	The service provides data to support genetic research. Its data analysis functions help with processing of genetic sequences in RNA-seq and ChIP-seq formats, and a database of previously processed experimental results provides points of comparison for newly collected datasets. These resources help researchers to evaluate expression and regulation of genes developed in their experimental work and in similar results from other studies. API methods support retrieval of a catalog of datasets already logged by the service along with search-based access to data for individual genes, experiments, datasets, or public samples. Methods allow selection of transcription factors and TFAS scores for a target gene or an experimental sample. The API gives access to genomic data files in BED or WIG format.	genetics,biology,science		http://www.programmableweb.com/api/geneprof	genetic sequences, RNA-seq, ChIP-seq formats	evaluate expression of genes, evaluate regulation of genes, individual genes, genomic data, XML,JSON,plain text,RDATA	Microarrays
153	GlobPlotter API	GlobPlotter is a SOAP-based API that allows users to plot the tendency within a protein for order/globularity and disorder. The plots generated by the API can be useful for designing constructs corresponding to globular proteins as is needed for many biochemical studies, particularly structural biology.	biology,science		http://www.programmableweb.com/api/globplotter	protein	protein order, protein disorder, design constructs of proteins, XML	Microarrays

154	GMOD API	GMOD, short for Generic Model Organism Database, is a project based on creating and managing genome-scale biological databases. GMOD provides a set of open-source tools to accomplish this. One of the tools provided is the GMOD RESTful API for querying genes and terms in different biological databases, such as FlyBase. The API is currently in private beta.	reference,science,biology		http://www.programmableweb.com/api/gmod	genome, biological data, genes	create genome database, manage genome database, XML,JSON	Microarrays
155	GOR protein secondary structure prediction API	This collection of three services -- GORI, GORIII, and GORIV -- provide predictions of secondary protein sequences using methods based on the information theory of Garnier, Osguthorpe, and Robson. It evaluates checks for 17 residues to determine scores for four possible conformation states, with correction for known decision constants. The highest score is taken to indicate the protein conformation. API methods support submission of a protein sequence from experimental data. The API returns predictions of secondary sequences associated with the input data based on estimates of conformational states.	science,biology,genetics		http://www.programmableweb.com/api/gor-protein-secondary-structure-prediction	protein sequence	predictions of secondary protein sequences, XML	Microarrays
156	H-InvDB API	The service provides access to the H-Invitational Database (H-InvDB) of human genes and genetic structure. The database documents research analyzine all human gene transcripts with annotations from the service provider describing genetic structures, observed variants, and highly detailed interactions of genetic sequences and processes. API methods support search against the database by individual gene identifier, keywords in description text, location on a particular chromosome or in relation to other genes, and more.	science,research,biology,medical		http://www.programmableweb.com/api/h-invdb	genes, genetic structure, gene transcripts	genes interactions, interactions of genetic sequences, interactions of genetic processes, gene identifier, location on chromosome, XML	Microarrays

157	INOH Pathway Database API	The service, Integrating Network Objects with Hierarchies (INOH), provides access to a database of pathways among bio-molecules in several organisms, including humans, mice, and rats. The pathways recorded by the service define molecular connections that allow signal transduction in an organism. This information is documented largely in published articles, so the database is built by extracting knowledge from text to develop a machine-processable repository. API methods support retrieval of graphs defining nodes and arcs (or edges) connecting them, based on selected criteria. Results delivered include the nodes involved in a particular pathway and the connections between them, distinguished by event type, including controlled events, controlling events, and others.	science,medical,biology,research		http://www.programmableweb.com/api/inoh-pathway-database	bio-molecules pathways	molecular connections, signal transduction, pathway, connections, XML	Systems Biology
158	JasparDB API	The service provides access to a database of factor-binding profiles depicting genetic makeups and structures typical of species. It provides biological researchers with lookup access to matrices defining genetic relationships documented through research. API methods generate profiles of transcription factor binding sites in varying formats, including Position Frequency Matrices (PFM), Position Weight Matrices (PWM), and Information Content Matrices (ICM). Methods support retrieval of all matrices or of a specific matrix specified by system ID or name. The API also supports search among matrices described in the system by topic-specific tags applied.	science,biology,genetics		http://www.programmableweb.com/api/jaspardb	genetic makeup, genetic structures	genetic relationships, position frequency matrices, position weight matrices, information content matrices, XML	Microarrays

159	Madeline API	The service provides analysis and formatting of data reflecting human genetic background for use in studies of genetic linkage and pedigree. It converts data about genetic markers for an individual to formats compatible with common software for linkage analysis. It also supports queries against genetic pedigree datasets to aid discovery of relationships and patterns. Visualization tools generate Postscript images for rapid review of large, complex datasets. The service is also available as installed software. API methods support submission of genetic marker data for analysis. Methods analyze the pedigree links represented in the data and format it for submission to common link analyzer packages. Methods also generate SVG images representing data and defining genetic links to recorded human pedigrees.	science,biology,genetics		http://www.programmableweb.com/api/madeline	genetic data, genetic markers	genetic analysis, genetic formatting, genetic pedigree, discover relationships, patterns, visualization, generate images, XML	Microarrays
160	MetNet API	MetNet is software to visualize, explore, statistically analyze and model transcriptomics, proteomics and metabolomics data in the context of a growing metabolic and regulatory network map of Arabidopsis, Soybean, and other species. The MetNet API is a programming library that provides direct-access to our MetNetDB database. It allows software developers to interface with a central repository of pathway-related data. It offers flexible query and data-retrieval methods for Java- and R-based applications that import biological network knowledge.	biology,database		http://www.programmableweb.com/api/metnet	metabolic, pathway, transcriptomics, proteomics, metabolomics	visualize, explore analyse, model transcriptomics, proteomics, metabolomics	Microarrays
161	MiMI/Metabolomics API	The service provides a data repository supporting analysis in systems biology of genetic and metabolomic effects and molecular interactions. Available data include the MiMI database of protein interaction characteristics, which gathers input from multiple databases and sources within the field of biology. The repository also draws on daily updates of PubMed resources and reports from biomedical research. This single point of access allows queries and analysis against multiple interrelated data sources. API methods support search by free text or by a specific gene identifier. Returned data can include known compounds, interactions, and reactions. Methods can also return a list of interactions discussed in biological literature, discovered via natural language processing.	science,medical,biology,research		http://www.programmableweb.com/api/mimimetabolomics	genetic, metabolic, molecular interactions, protein, gene	genetic analysis, metabolic analysis, protein interactions, gene identifier, XML	Systems Biology

162	MitoMiner API	The service provides mitochondrial data for a range of organisms. It collects a number of interrelated datasets associated with genetic and protein structures, allowing single-source access to information about their expression in living organisms. It also maintains and provides ontologies for describing genes. API methods support flexible queries against the database for mathces to protein names, UniProt keywords, gene names, or species. Methods give access to datasets covering mitochondrial proteomics, gene ontologies, and metabolic pathways. The API also gives access to records of associations of human disease with mitochondrial profiles.	science,research,biology		http://www.programmableweb.com/api/mitominer	genetic structures, protein structures, genes, metabolic pathways, mitochondrial profiles	mitochondrial data, protein names matches, gene names, species, mitochondrial proteomics, gene ontologies, metabolic pathways, JSON	Systems Biology
163	MultAlin API	The service detects multiple alignments of elements within protein sequences submitted. Comparison of sequences detected in multiple sources can reveal similarities that indicate shared genetic heritage, allowing researchers to infer common background. API methods support submission of a set of protein sequences in Pearson-Fasta format. The API returns alignments detected in the sequences by applying the ClustalW algorithm, allowing researchers to draw conclusions about common genetic origins represented by the sequences.	science,biology,genetics		http://www.programmableweb.com/api/multalin	protein sequences	comparison of protein sequences, XML	Microarrays
164	NCBI Conserved Domain Database (CDD) API	The service provides access to a database of molecular protein building blocks that recur in different combinations as part of organisms' genetic makeup. The search function helps to uncover associations between chemical elements of genetic structures documented in disparate data sources. API methods support selection of databases to search and configuration of conserved domains, as defined by NCBI, to match. Methods allow selection of search modes from basic to advanced, with complex Boolean queries, and filtered results. Methods also allow specification of Entrez-based protein designations in search queries.	science,biology,research		http://www.programmableweb.com/api/ncbi-conserved-domain-database-cdd	molecular protein building blocks, genetic maekup	chemical elements, genetic structures, protein designations, Text	Microarrays

165	ncRNA fRNAdb API	The service provides search access to a database of functional RNA sequences. It is part of a web portal of bioinformatics tools supporting genetic research specific to functional RNA, it is a comprehensive listing of non-coding RNA sequences. International scientists maintain data for open access within the genetic research community. API methods support submission of a search query to retrieve matches in the database. Methods also support retrieving complete details of individual listings, with output available in several mapping and sequencing formats, in addition to XML.	science,biology,search,genetics		http://www.programmableweb.com/api/ncrna-frnadb	RNA sequences, genetic	genetic research, RNA sequences, XML	Microarrays
166	Pacific Biosciences SMRT Pipe API	The service provides a collaborative platform for molecule sequencing within the life sciences. It provides information about a proprietary technology for sequencing, SMRT, along with data and analytical software to help process it. Tools for accessing and converting files allow manipulation of data resources for processing in different environments. API methods support management of sequencing data output, secondary data analysis, control of analysis protocols, and validation of sample sheets. Separate APIs support conversion of data files between different formats.	science,chemistry,biology,research		http://www.programmableweb.com/api/pacific-biosciences-smrt-pipe	molecule sequence	sequencing, SMRT, data analysis, control, analysis protocols, JSON	Microarrays
167	Pathway Commons API	The API provides a convenient point of access to information about biological pathways involving a specified physical entity (e.g. protein or small molecule) collected from public pathway databases. It gives machine-readable access to the same information browsed and searched by biologists and downloaded by computational biologists in BioPAX format for global analysis. Users can also download and install the cPath software to create a local mirror. All data is freely available, under the license terms of each contributing database.	medical,research,biology		http://www.programmableweb.com/api/pathway-commons	biological pathways, protein, small molecule, pathway	protein, small molecule, biological pathways, XML	Systems Biology
168	PcProf predict physico-chemical profiles of proteins API	The service provides a profile of a submitted protein sequence, describing its characteristics in relation to a list of properties established for known structures. Characteristics described include hydrophilicity/hydropathy, flexibility, antigenicity according to two measures, accessibility, and transmembranous helices. The profiles generated predict the behavior o be expected of the sample genetic structure. API methods support submission of a protein structure detected in research results. The API returns measures of key physical and chemical properties known to be associated with such structures to form a profile of the sample.	science,biology,genetics		http://www.programmableweb.com/api/pcprof-predict-physico-chemical-profiles-of-proteins	protein sequence, hydrophilicity, hydropathy, antigenicity	genetic structure, protein structure, chemical properties, XML	Systems Biology

169	PREDATOR protein secondary structure prediction API	The service predicts secondary protein structures by analyzing amino acid sequences submitted and detecting hydrogen-bonded residues within those structures. Potential for hydrogen bonds is associated with resulting secondary structures, providing a partial indicator that may be used in combination with outputs of other services. API methods support submission of amino acid sequences to generate a job request and begin processing. The API returns indicators of hydrogen-bonded residues detected within the input data for use in secondary structure prediction. Methods also allow for control of the service, including status monitoring and cancellation of current processing jobs.	science,biology,genetics		http://www.programmableweb.com/api/predator-protein-secondary-structure-prediction	protein structures, amino acid sequences, hydrogen-bonded residues	protein structures, amino acid sequences, structure prediction, XML	Systems Biology
170	Pseudoviewer API	The service provides tools for creating visual representations of genetic structures involving RNA. It converts experimental data to depict secondary structures and pseudoknot structures. By providing a consistent, flexible toolset, it helps researchers overcome problems with variations in data structures and software incompatibilities. API methods support conversion of large data sets to visualize complex RNA secondary structures with pseudoknots. Methods accept data uploads for RNA sequences in bracketed or paired format along with specifications for the output image desired. The service then generates the requested image representing relationships between RNA components implied by the data.	science,biology,genetics		http://www.programmableweb.com/api/pseudoviewer	genetic structures, RNA	depict structures, pseudoknot structures, visualize RNA structures, RNA sequences, RNA relationships, XML	Microarrays
171	RCSB Protein Data Bank API	The Protein Data Bank (PDB) archive is the single worldwide repository of information about the 3D structures of large biological molecules, including proteins and nucleic acids. It is managed by the Research Collaboratory for Structural Bioinformatics (RCSB). The RCSB PDB allows users to access data through one of two methods: Search services: to return a list of IDs (i.e. PDB IDs, chain IDs, ligand IDs), Fetch services: to return data given a ID. The API uses RESTful calls and responses are formatted in XML.	database,Protein,science,biology,pdb		http://www.programmableweb.com/api/rcsb-protein-data-bank	protein structures, biological molecules, proteins, nucleid acid	protein structures, XML	Systems Biology

172	Reputer API	The Reputer API helps users discover and visualize repeats in whole genomes or chromosomes. Repeat types that can be searched for include forward(direct) matches, reverse matches, complement matches, and palindromic matches. Reputer's functions are freely offered in a limited capacity through its web console and SOAP API. Downloading the software requires an additional licensing agreement.	genetics,science,biology		http://www.programmableweb.com/api/reputer	genome, chromosome	discover, visualize repeats in genomes, visualize chromosomes, matches of chromosomes, matches of genomes, XML	Systems Biology
173	Retrotector API	The service from the University of Uppsala in Sweden provides analysis of DNA sequence data to generate a viewable representation of genetic links represented. A Java-based viewer program is available to interpret analytical output and depict genetic structures for deeper investigation. API methods support submission of a DNA sequence data in FASTA format or as raw data (possibly including position numbers). Sequence size may range from 5 to 10,000 KB in length. Methods support queue management to remove or update analysis jobs along with status monitoring for either an entire queue or a single job. The API also provides for accessing analytical output files.	science,biology,genetics		http://www.programmableweb.com/api/retrotector	DNA sequence, FASTA format	genetic representation, genetic structures, XML	Microarrays
174	RNAfold API	RNAfold is an interface for RNA folding and sequence design. It can be used to calculate secondary structures of RNA sequences. RNAfold is available as a web console or as a SOAP API.	biology,science,genetics		http://www.programmableweb.com/api/rnafold	RNA sequences	RNA folding, sequence design, RNA sequences, XML	Microarrays
175	RNAforester API	RNAforester is a tool for comparing RNA secondary structures. It supports the computation of pairwise structures and the multiple alignment of structures. RNAforester is available as a web console or a SOAP API.	biology,genetics,science		http://www.programmableweb.com/api/rnaforester	RNA structures	RNA structure alignment, compare RNA structures, XML	Microarrays

176	RNAshapes API	Given an RNA sequence, RNAshapes can help determine its probable shape. RNAshapes computes a small set of representative structures of different shapes as well as accumulated shape probabilities. It also offers comparative predictions of consensus structures. RNAshapes is available as a web console, a downloadable program, and a SOAP API.	science,genetics,biology		http://www.programmableweb.com/api/rnashapes	RNA sequence	RNA shape, predict consensus structures, XML	Microarrays
177	SignalP/SignalP4 API	The service provides predictions of genetic structures based on neural network algorithms and Markov statistical models. It analyzes data about the frequency of amino acid sequences for various organisms and generates likely existence and locations of signal peptide cleavage sites to be expected in their genetic structures. Organism groups covered include gram-positive and gram-negative prokaryotes as well as eukaryotes. The same provider maintains an alternative service, as well. API methods support submission of the amino acid sequence to be analyzed along with an organism type, preferred prediction method, and desired statistical prediction certainty threshold. Methods also support monitoring the service processing queue and retrieving the completed analytical result.	science,biology,genetics		http://www.programmableweb.com/api/signalpsignalp4	genetic structures, amino acid	genetic structures, amino acid sequences, XML	Microarrays
178	SIMPA96 API	The service provides predictions of secondary protein and nucleic acid sequences implied by the presence of known primary sequences. Its analysis is based on the nearest neighbor methodology, which tests for homologous chromosomes and infers secondary structures associated with them. API methods support submission of a primary gene sequence and retrieval of predictive data describing associated secondary sequences. Methods also allow status checks to monitor processing under the API and cancellation of current analysis operations.	science,biology,genetics		http://www.programmableweb.com/api/simpa96	protein sequences, nucleic acid sequences, gene sequence	protein sequences, nucleic acid sequences, homologous chromosomes, XML	Microarrays

179	SSearch protein similarity search API	The service compares a submitted protein sequence against a reference database with analysis of similarities to known sequences. The output helps to identify a sequence and establish its genetic relationships to established structures while also noting variations from known patterns. This analysis puts a sample in context of prior research and links it to existing genetic structures. API methods support submission of a protein sequence identified in research to initiate the comparison. The API identifies similar sequences from its reference database, which allows the researcher to understand links to established genetic structures represented in the data.	science,biology,genetics		http://www.programmableweb.com/api/ssearch-protein-similarity-search	protein sequence, genetic structures	identify sequence, genetic relationships, variations from known patterns, genetic structures, XML	Microarrays
180	VisANT API	The service provides visualization tools to represent networks and pathways within biological research. Available as a web service or installed software, it ingests data about organisms and species and creates images depicting links and relationships between them. Interactions within an active community drive development of the graphical tools to create effective depictions for researchers. API methods support defining characteristics of the nodes of a visualization, including the links between them that determine the graphic representation. Methods support lookup in species and organism records to help define node descriptions.	science,biology,visualization,graphics		http://www.programmableweb.com/api/visant	biological pathways	visualization pathways, creates images, CSV,XML	Systems Biology
181	WebScipio API	WebScipioTrack this APIThe service defines the gene structure implied by a protein sequence within a particular genome. It aides in research projects to define gene sequencing by analyzing a protein sequence and identifying the genes defined, with accommodation for sequencing errors and errors and ambiguously defined genes, even where complete genomes are not fully described. The service provides locations within the genome where the submitted protein sequence could appear, which indicates the genes corresponding to the sequence. API methods support submission of a protein sequence with data about its source within a particular genome. Methods return comprehensive identification of genes defined by the sequence and identifies available genetic profiles for species characterized by the submitted profile and genes.	science,research,biology,genetics		http://www.programmableweb.com/api/webscipio	protein sequence, genome, gene	gene structure, gene sequencing, identify genes, genome location,genetic profiles, JSON	Microarrays

182	Aetna CarePass API	CarePass by Aetna is an online portal that allows users to access health data, provider data, medicine information, and more health information. The CarePass API allows developers to access and integrate the functionality and data from CarePass with other applications and to create new applications. Some example API methods include retrieving claim information, searching and retrieving FDA and drug information, and accessing medicine pricing.	medical,health,Insurance,drugs		http://www.programmableweb.com/api/aetna-carepass		medicine information, health information, FDAm drug information, JSON	Medical
183	AIDSinfo API	AIDSinfo offers access to the latest, federally approved HIV/AIDS medical practice guidelines, information on HIV/AIDS treatment, clinical trials, and other HIV/AIDS-related research information for health care providers, researchers, people affected by HIV/AIDS, and the general public. The AIDSinfo API provides access to the AIDSinfo drug database fact sheets in XML format. The API returns information from both the professional and patient versions of the AIDSinfo drug database fact sheets based on input query strings. It uses RESTful calls and responses are formatted in XML.	government, medical,health,reference		http://www.programmableweb.com/api/aidsinfo	HIV, AIDS, drug, XML	HIV, AIDS treatment information, drug info, XML	Medical
184	Allen Brain Atlas API	The Allen Brain Atlas is a collection of public online resources integrating extensive gene expression and neuroanatomical data. It is especially rich in resources relating to human and mouse neuroscience, but also includes some resources on non-human primates. The Allen Brain Atlas API consists of a set of URLs that make most of the data behind the project available for download.	medical,science		http://www.programmableweb.com/api/allen-brain-atlas	gene expression, neuroanatomical data	gene expression, neroanatomical data, XML	Medical
185	Beddit API	The service provides monitoring of a user's sleep patterns. A bedside device detects physical conditions and activities, uploading data to the service for later access by the sleeper, health care providers, or personal trainers. Data tracked include raw ballistocardiography (BCG) signals, heart rate measurements and variability (HRV), minute-by-minute actigraphy, and classification of the subject as asleep or awake at the moment of data collection. hods support authorization of access to a user account, followed by retrieval of information about the user and the timeline data collected by the bedside monitor. Requests can specify particular date and time ranges to gather data recorded during those spans. In addition to BCG and HRV, returned data report on conditions in the room such as light and sound levels.		medical,health,monitoring,sleep	http://www.programmableweb.com/api/beddit	physical conditions, activities	sleep patterns, ballistrocardiography signals, heart rate measurements, variability, minute-by-minute actigraphy, JSON	Medical

186	Bililite API	The service provides pediatric charting functions that convert data submitted to images for web display and other purposes. Data can be entered via interactive forms or submitted as URL parameters, and the service completes processing to generate the requested chart displaying the data. Available graphs include height, weight, body mass index, peak flows, and bilirubin levels.API methods accept requests specifying the chart type (bilirubin, height and weight, BMI, blood pressure, head circumference, or peak flows) and data from a sample of patients or an individual patient. The API generates an image URL to be incorporated into another web page or other application.	vizualization, charts,health care,health, medical		http://www.programmableweb.com/api/bililite	pediatric functions	image, height graph, weight graph, body mass, peak flows, bilirubin levels, blood pressure, head circumference, PNG	Medical
187	bioNMF API	The bioNMF API uses Non-negative Matrix Factorization (NMF) to analyze biomedical data. Users can choose from three variations of the algorithm; standard, divergence, and non-smooth NMF. This API can be used to find cluster genes and highly related samples, to determine the most suitable number of sample clusters in a given dataset, or just to perform an NMF. This API is accessible via SOAP protocols.	science,bioinformatics,medical,analysis		http://www.programmableweb.com/api/bionmf	non-negative matrix factorization, NMF, biomedical data	cluster genes, NMF, XML	Medical
188	Biosemantics ACCCA API	The service provides annotations for patient records to consistently document concepts related to problems detected in clinical settings, treatments defined for medical problems, and tests for monitoring patient conditions. The service combines input from six separate annotation systems to generate aggregate results that may be recorded to patient charts or other records. A training mode allows users to acclimate to the service and generate reliable results. API methods support submission of plain text from clinical records and returns annotations identifying clinical concepts for additional analysis. Methods apply a simple voting algorithm to combine results of separate annotation schemes for the final aggregate annotations.	healthcare,health,medical		http://www.programmableweb.com/api/biosemantics-accca	patient records, clinical, patient conditions, clinical record	treatments, identify clinical concepts, analysis, XML	Medical

189	ChemBank API	ChemBank is a database that stores information on hundreds of thousands of small molecules and on hundreds of biomedically relevant assays. The ChemBank API allows users to download all the project, assay, plate, and well measurement data within ChemBank. Substructure and similarity search services are also provided.	chemistry,science,biology,medical,deadpool		http://www.programmableweb.com/api/chembank	small molecules, biomedically assays	molecular structure, molecular matching, XML	Medical
190	China Cancer Database API	The China Cancer Database is a repository for information about the incidence, mortality, prognosis, therapy, and prevention of cancer. It was established by the Chinese Ministry of Science and Technology to create a uniform and nationwide cancer database. Specific information can be retrieved using SOAP APIs. Documentation for the China Cancer Database is available in both Chinese and English.	china,cancer,medical		http://www.programmableweb.com/api/china-cancer-database	cancer	cancer prognosis, cancer therapy, prevention of cancer, XML	Medical
191	Cigna Health API	Cigna is a health services company that provides integrated health care and related plans as an insurance provider. The Cigna Health API allows the user to access real-time health data. The service allows users to access health data to better serve customers, create applications with personalized data, share test applications, and deploy applications to production. The API uses REST calls and returns XML or JSON.	medical,Data,health,merchant,realtime		http://www.programmableweb.com/api/cigna-health	health data	JSON,XML	Medical
192	cPath API	cPath is a database and software suite for storing, visualizing, and analyzing biological pathways.	database,bioinformatics,medical		http://www.programmableweb.com/api/cpath	biological pathways	store biological pathways, visualize biological pathways, analyse biological pathways, XML	Medical

193	DailyMed API	The U.S. Department of Health and Human Services (HHS), through the National Library of Medicine, provides users with access to current Structured Product Language (SPL) information about marketed drugs. The data provided by this service is the most recent provided to the FDA (such as FDA labels/package inserts), and also includes information about warnings and in-progress FDA product reviews. Users can query this RESTful service using a variety of parameters, including prescription or over the counter, human or animal drugs, drug name, drug imprint data, and National Drug Code (NDC). This free service returns data as XML or JSON based on user specificaiton.	medical,health,drugs		http://www.programmableweb.com/api/dailymed	drug	drug data, drug name, XML,JSON	Medical
194	DGIdb API	The Drug Gene Interaction Database (DGIdb) is a database of known and potential drug-gene interactions. The web interface allows users to search for drug-gene interactions by gene, search for genes by category, or browse categories of genes. The API allows users to search for interactions or to retrieve interaction types, drug types, interaction sources, source trust levels, gene categories, and related genes. Information on drug-gene interactions is collected from multiple resources, including DrugBank, TTD, and PharmGKB.	genetics,medical,science,database		http://www.programmableweb.com/api/dgidb	drug, gene	drug-gene iinteraction, drug types, gene categories, related genes, JSON	Medical
195	dkCOIN API	dkCOIN (NIDDK Consortium Interconnectivity Network) provides seamless public access to large pools of data relevant to the mission of NIDDK (National Institute of Diabetes and Digestive and Kidney Diseases). Available resources includes everything from raw genetic information to scholarly articles. The dkCOIN API allows users to search and access its collections and resources. With an account, users can upload and edit their own resources for public use.	science,medical		http://www.programmableweb.com/api/dkcoin	diabetes, digestive, kidney disease, genetic information	genetic information, XML	Medical
196	Dossia API	Dossia aggregates users' health information into one convenient and safe Web-based platform. From this platform, users can utilize their data and access personalized health tools. The Dossia API allows client applications to securely read and update a Dossia participant's health data. Developers can read and write medications, allergies, immunizations and other XML document types.	health		http://www.programmableweb.com/api/dossia	allergy, medications, immunization	allergy, medications, immunization, XML,JPG,PNG,PDF	Medical

197	Drugle API	The service provides a semantic search engine for authoritative information on pharmacology and medical drugs free of provider bias. It provides a general reference tool for health-care providers, patients and families, researchers, and medical students. Health information systems can implement the service as a component of decision support technology. API methods support submission of search queries and retrieval of results. Integration demos illustrate incorporation of the service with health information sites.	medical,search,drugs		http://www.programmableweb.com/api/drugle	medical drugs	XML	Medical
198	EBI API	The EBI Web services let you access nucleic acid, protein sequence, and macromolecular structure data. Choose from about 35 EBI Web Services. The European Bioinformatics Institute (EBI) is part of the European Molecular Biology Laboratory (EMBL). The Web services offer DNA and protein sequences, text mining, structure comparisons, various BLASTs, transmembrane topology predictions, microarray searching, and more.	medical,bioinformatics,science		http://www.programmableweb.com/api/ebi	nucleid acid, protein sequence, macromolecular structure	DNA sequence, protein sequences, text mining, structure comparison, BLAST, transmembrane topology predictions, microarray searching, XML	Medical
199	GeneCruiser API	GeneCruiser provides a single point of access to genomic information freely available from multiple public data sources. With GeneCruiser, users can find variations in genes or find the gene to which a variation is linked. Users can also convert genes to Affymetrix probe identifiers and use those identifiers to retrieve requested information. The GeneCruiser API allows users to access the GeneCruiser functionality from within their own applications.	science,medical		http://www.programmableweb.com/api/genecruiser	genomic information	gene variations, gene, convert genes to Affymetrix probe, XML	Medical

200	Healthnotes API	Helathnotes is a content source for consumer self-care wellness decisions. The Healthnote API consists of two services: the concept service and the element service. The concept service provides metadata on health and wellness concepts and exposes the relationships between concepts. The element service provides content in a contextual, highly-atomized form giving users a different perspective on the content. Ultimately, the API provides access to self-care decision support by condition, nutritional supplement, medicine, diet, and food.	reference,health,medical		http://www.programmableweb.com/api/healthnotes	self-care, nutricional supplement, medicine, diet, food	self-care, nutricional supplement, medicine, diet, food	Medical
201	HIPAASpace API	Helps medical and health-care applications to support ICD-10 codes in incoming EDI files via lookup services. Includes lists like National Provider Identifier, Healthcare Procedure Conding System Code, National Drug Code, International Classification of Diseases, and others	medical,HIPAA,reference		http://www.programmableweb.com/api/hipaaspace	medical care, drug, disease classification	XML,JSON	Medical
202	HIV Drug Research Center API	The HIV Drug Research Center provides a pair of APIs for predicting the susceptibility of mutated HIV to a variety of drugs. One API predicts the susceptibility of mutations in HIV protease to amprenavir, atazanavir, indinavir, lopinavir, nelfinavir, ritonavir, and saquinanvir. The other predicts the susceptibility of mutations in HIV reverse transcriptase to lamivudine, abacavir, zidovudine, stavudine, zalcitabine, didanosine, emtricitabine, and tenofovir. These APIs are available to users via REST and XMPP.	medical,aids		http://www.programmableweb.com/api/hiv-drug-research-center	HIV, AIDS, drug	predict HIV mutations, XML	Medical
203	How are you API	How are you? is a free service for NHS patients and healthcare professionals that provides a convenient way to host patient health records online. Patients can control the contents of these records as well as how much of that content is shared and with whom. How are you? also provides users with information about medical conditions in plain English with direct links to external reference sources. The How are you? API uses REST calls to enable users to retrieve, edit, and delete information on the medical conditions, medications, and allergies in a patient's record. The API also lets users add and retrieve measurements taken over time such as blood pressure, blood glucose level, caloric intake, height, weight, and physical exercise.	healthcare,health,medical,uk		http://www.programmableweb.com/api/how-are-you	NHS, medical conditions	medical conditions, allergies, blood pressure, blood glucise level, caloric intake, height, weight, physical exercise, JSON	Medical

204	Kegg API	The increasing amount of genome sequence data is the basis for understanding life as a molecular system and for developing medical, pharmaceutical, and other practical applications. Since 1995 we have been developing knowledge-based methods for uncovering higher-order systemic behaviors of the cell and the organism from genomic and molecular information.	bioinformatics,Data,medical		http://www.programmableweb.com/api/kegg	genome sequence, molecular, medical	cell behavior, genomic information, molecular information	Medical
205	Lexicomp API	Lexicomp is a provider of drug information and clinical content for the healthcare industry. Lexicomp offers an Internet based solution for providing clinicians this content. Information from the databases and modules in Lexicomp Online are made available via an XML API and can be used in third party applications. Full documentation is not publicly available.	reference,medical,drugs,health		http://www.programmableweb.com/api/lexicomp	drug, clinical	drug, clinical, XML	Medical
206	lifeIMAGE API	lifeIMAGE is a service for healthcare providers, doctors, patients, and hospitals to manage share diagnostic imaging records with each other. The lifeIMAGE API allows developers to access and integrate the functionality of lifeIMAGE with other applications and to create new applications. Some example API methods include accessing patient records, managing account information, and sharing patient records.	medical,health		http://www.programmableweb.com/api/lifeimage	patient records	diagnostic imaging records	Medical
207	Manage BGL Diabetes API	ManageBGL is an online diabetes management platform. ManageBGL allows users to upload blood sugar readings, insulin doses and carbohydrate intake from their meter, cell phone or web browser. Once uploaded to the cloud, the information is accessible over the internet and can be shared in real time with teachers, parents, healthcare providers and other care givers. The RESTful API allows users to pull information from ManageBGL directly into an excel spreadsheet, or view recent blood sugar highs and lows with a news reader. Default formats are XML, JSON, RSS and Atom.	diabetes,health,management,medical		http://www.programmableweb.com/api/managebgl-diabetes	diabetes, blood sugar readings, insulin doses, carbohydrate intake	blood sugar highs, blood sugar lows, XML,JSON,RSS,Atom	Medical
208	myHealthAccount API	myHealthAccount is a patient centric medical record service from the Swedish company Infogosoft. Clinics can use myHealthAccount modules to send medical records and images to patients accounts and patients can use myHealthAccount to gather their medical records, vaccinations, and drug lists all in one place. The myHealthAccount REST API allows developers to write applications that access myHealthAccount services. API methods exist for folders, vaccines, and documents. Most responses are in JSON format. An API key is required.	medical,health,records,sweden		http://www.programmableweb.com/api/myhealthaccount	patient record, medical record	medical records, images, vaccinations, drug list, JSON	Medical

209	National Cancer Institue caDSR API	The National Cancer Institute (NCI), a sub agency of the National Institute of Health (NIH), offers the Cancer Data Standards Registry and Repository (caDSR) API to provide users with programmatic access to a database of Common Data Elements (CDE's) created by the NCI to manage biomedical data. The caDSR contains Unified Markup Language (UML) models representing information domains developed by collaborating research partners, and submitted to the database. The semantics of the data elements in these models are extracted and transformed into administered components of the database, while the resulting collection of related CDE's are classified as part of the model and made visible as a collection. This free API operates over REST and returns data via XML and HTML.	medical,cancer,Data,standards		http://www.programmableweb.com/api/national-cancer-institue-cadsr	cancer, biomedical data	cancer, biomedical data, XML,HTML	Medical
210	National Cancer Institute SEER API	The Surveillance, Epidemiology and End Results (SEER) Program, a service of the National Cancer Institute, is a cancer statistics resource. Collected data includes information on the incidence, prevalence and survival from specific geographic areas within the U.S., as well as cancer mortality for the entire country. The SEER API is a RESTful service supporting various program datasets and algorithms, and is available to developers who wish to incorporate SEER resources into their own systems. Exposed resources include Collaborative Staging, the Hematopoietic and Lymphoid Neoplasm Database, NAACCR documentation, the Antineoplastic Drugs Database, and incidence site recode variables. The API returns JSON formatted responses, communicates over HTTPS, and requires a free account and API key.	medical,cancer,statistics		http://www.programmableweb.com/api/national-cancer-institute-seer	cancer, cancer mortality	antineoplastic drugs, hematopoietic, lymphoid neoplasm, JSON	Medical
211	National Drug File-Reference Terminology API	The National Drug File-Reference Terminology (NDF-RT), developed by the Veterans Health Administration, provides clinical information about medications, including therapeutic intent, mechanism of action, physiologic effect and drug-drug interactions. Developers can use the API to retrieve data from the NDF-RT. The API uses both REST and SOAP protocol and responses are formatted in XML and JSON.	government,reference,medical		http://www.programmableweb.com/api/national-drug-file-reference-terminology	medical, clinical	clinical information, medications, therapeutic intent, drug-drug interaction, XML,JSON	Medical

212	National Institute on Drug Abuse Drug Screening Tool API	In an effort to connect developers with the tools needed to host and share the Government’s drug abuse research and prevention information, the National Institute on Drug Abuse hosts an API through which they expose their Drug Use Screening Tool. The Drug Use Screening Tool is an interactive multiple-choice quize built to help clinicians identify risky substance use in their adult patients. The API supports GET calls to retrieve questions and results and POST calls to submit answers. The API will return JSONP formatted responses in plain text through HTTP.	screening,drugs,medical,quiz		http://www.programmableweb.com/api/national-institute-on-drug-abuse-drug-screening-tool	drug, substance	drug screening, identify risky substance, drug substance, JSON,JSONP	Medical
213	National Library of Medicine ChemSpell API	The National Library of Medicine (NLM) is the world's largest medical library. The Library collects materials and provides information and research services in all areas of biomedicine and health care. The ChemSpell Web Service API provides chemical name spell checking and chemical name synonym look-up. ChemSpell contains more than 1.3 million chemical names related to organic, inorganic, pharmaceutical, toxicological, and environmental health topics. Developers can use the API to write applications that connect remotely to the ChemSpell Web service. The API uses SOAP calls and responses are formatted in XML.	science,medical,reference		http://www.programmableweb.com/api/national-library-of-medicine-chemspell	medical, biomedicine, health care	chemical name checking, chemical name synonym look-up, XML	Medical
214	Open mHealth API	Open mHealth is an open architecture software service that enables sharing of mobile health data from various and multiple data sources. The Open mHealth API allows developers to access and integrate the data and functionality of Open mHealth with other applications and to create new applications. Some example API methods include retrieving data from specified streams, posting data, and searching data.	opendata,health,mhealth,medical,Data		http://www.programmableweb.com/api/open-mhealth		JSON	Medical
215	OpenTox API	OpenTox is a framework that can be used in the creation of predictive toxicology applications. It can be integrated with other APIs to provide users with access to a variety of distributed toxicological resources including data, computer models, validation, and reporting. OpenTox is working to meet the requirements of the REACH legislation for providing alternatives to animal experiments for toxicity testing.	medical,science,tools		http://www.programmableweb.com/api/opentox	medical	toxicology application, XML,RDF	Medical

216	Pathway Commons API	The API provides a convenient point of access to information about biological pathways involving a specified physical entity (e.g. protein or small molecule) collected from public pathway databases. It gives machine-readable access to the same information browsed and searched by biologists and downloaded by computational biologists in BioPAX format for global analysis. Users can also download and install the cPath software to create a local mirror. All data is freely available, under the license terms of each contributing database.	medical,research,biology		http://www.programmableweb.com/api/pathway-commons	biological pathways, protein, small molecule	biological pathways, protein, small molecu;e analysis, XML	Medical
217	PDBj API	Use the Protein Data Bank Japan (PDBj) API to get protein sequence and structure data, useful in biotech research and other research. Protein Data Bank Japan maintains a repository of macromolecular structures and offers tools, in collaboration with the RCSB in the US and the MSD-EBI in the EU. PDBj is supported by JST-BIRD. The head of PDBj is a professor at Osaka University.	japan,bioinformatics,medical		http://www.programmableweb.com/api/pdbj	protein sequence, protein structure, macromolecular structures	protein sequence, protein structure, macromolecular structures, Atom,OpenSearch	Medical
218	Pillbox API	Pillbox enables rapid identification of unknown solid-dosage medications (tablets/capsules) based on physical characteristics and high-resolution images. Pillbox's data and search engine are also accessible through an API. The API provides access to the identification system, data, and medication images. Medications can be identified by physical characteristics (imprint, size, shape, etc.), as well as drug name (brand or generic), inactive ingredients, drug label author, and DEA schedule and returns XML-formatted data. Access to documentation requires a password.	reference,medical		http://www.programmableweb.com/api/pillbox	drug, medication, tablet, capsules	medication images, drug name, inactive indredients, XML	Medical
219	Promedas API	Promedas provides a clinical expertise system to medical professionals. The Promedas API can be integrated into existing medical systems that contain a patient file database. Using this data, Promedus can provide a differential diagnosis based on the data contained in a patient's file. The API is currently in a developer beta. To access the API, contact Promedas.	health,medical,diagnosis,tools		http://www.programmableweb.com/api/promedas	medical, patient file, data, diagnosis		Medical

220	RCSB Protein Data Bank API	The Protein Data Bank (PDB) archive is the single worldwide repository of information about the 3D structures of large biological molecules, including proteins and nucleic acids. It is managed by the Research Collaboratory for Structural Bioinformatics (RCSB). The RCSB PDB allows users to access data through one of two methods: Search services: to return a list of IDs (i.e. PDB IDs, chain IDs, ligand IDs), Fetch services: to return data given a ID. The API uses RESTful calls and responses are formatted in XML.	database,Protein,science,biology,pdb		http://www.programmableweb.com/api/rcsb-protein-data-bank	protein, large biological molecules, nucleid acid	protein, nucleid acid, large biological molecules, XML	Medical
221	RxNorm API	RxNorm provides normalized names for clinical drugs and links its names to many of the drug vocabularies commonly used in pharmacy management and drug interaction software. There are two RxNorm APIs (SOAP/WSDL and RESTful) available to provide developers with functions for retrieving RxNorm data from the most current RxNorm data set. Data can be returned in XML or JSON.	science,reference,medical		http://www.programmableweb.com/api/rxnorm	clinical drug	clinical drugs, drig names, pharmacy, drug interaction, XML,JSON	Medical
222	RxNorm Prescribable API	The service provides listings for a subset of drugs available to be prescribed in the United States from the larger list of all prescription medications maintained by the provider's RxNorm service. Prescribable listings also include selected popular over-the-counter (OTC) medications that doctors frequently prescribe. The listing omits drugs available only in countries other than the U.S. and those usable only for veterinary purposes. API methods support retrieval of an updated list of drugs with correct names, ingredients, and system identifiers. Methods also support access to descriptive information, such as NDC and UNII codes, relationships to other drugs on the list, and rankings by prescription frequency.	health,prescription,medical		http://www.programmableweb.com/api/rxnorm-prescribable	drug, veterinary	XML	Medical
223	SciBite API	SciBite is a drug discovery search engine that continuously scans the internet for new drug-related information. It helps users find new drugs and drug data by tracking the latest news, clinical trials, grants, and blogs relating to pharmaceutical and biotechnology developments. The SciBite API provides programmatic access to its drug discovery data so that other entities can make use of it, and so that informaticians can find new connections within the data.	health,science,textmining,medical		http://www.programmableweb.com/api/scibite	drug	drug-related information, drug discovery, drug data, XML,JSON,JSONP,RSS,Text	Medical

224	SeqHound API	SeqHound: biological sequence and structure database as a platform for bioinformatics research. SeqHound has been developed as an integrated biological sequence, taxonomy, annotation and 3-D structure database system. It provides a high-performance server platform for bioinformatics research in a locally-hosted environment.	bioinformatics,medical,database		http://www.programmableweb.com/api/seqhound	biological sequence	biological sequence, biological structure, XML	Medical
225	Sierra Stanford HIV API	The service provides access to a database developed by Stanford's HIVdb project to detail resistance of human immunodeficiency virus to established drug treatment regimens. Data provide scores according to the Stanford HIV Drug Resistance Algorithm for 17 protease and RT inhibitor compounds approved by the U.S. Food and Drug Administration (FDA) as treatments for HIV. Data also provide comments to guide evaluations and applications to form judgments of likely levels of drug resistance. API methods support submission of nucleic acid sequences to retrieve algorithmic scores predicting resistance of the described virus strain to known HIV treatment drugs. An XSL transform template is available to render the XML web service results as human-readable HTML.	healthcare,health,medical		http://www.programmableweb.com/api/sierra-stanford-hiv	HIV drug, nucleid acid sequences	HIV drug treatment, XML	Medical
226	SLAP API	SLAP is a drug target prediction service provided by the Cheminformatics group at the School of Informatics and Computing at Indiana University. The RESTful SLAP APIs enable users to perform drug target predictions against hundreds of proteins, to find drug similarities based on polypharmacology profiles, and to find drugs similar to an input drug.	medical,science,prediction		http://www.programmableweb.com/api/slap	drug, protein	drug similarity	Medical
227	STEPStools API	The service provides information about prescription medication suitable for pediatric patients based on diagnosis, age, body size, and other relevant conditions. Its knowledge base covers a full range of illnesses and prescription medicines used to treat them, including rounding of dosages and adjustment of formulations appropriate for small children. API methods support search across the knowledge base by illness or health condition, medication, and patient characterstics. Methods return a ranked list of recommended formulations and doses for matching medications, with allowance for patient age and body weight. These recommendations are scored. Rankings reflect recommendation scores determined by the medication matching algorithm.	medical,health		http://www.programmableweb.com/api/stepstools	pediatric patient, diagnosis, medicines, illness	medication matching, patient characteristics, XML	Medical

228	The Cancer Genome Atlas API	The Cancer Genome Atlas (TCGA) is a project to improve the understanding of the molecular basis of cancer through the application of genome analysis technologies, including large-scale genome sequencing. TCGA offers a collection of APIs that provide REST-based, programmatic access to its information resources. Available information includes annotations, data matrices, data reports, project metadata, and biospecimen metadata.	medical,cancer		http://www.programmableweb.com/api/the-cancer-genome-atlas	cancer genome, genome analysis, genome sequence	data matrices, project metadata, biospecimen metadata, annotationsXML,JSON	Medical
229	Tracknburn FoodData API	The service provides a database of nutritional information for common food products. Information includes nutritional component values such as calories, fat percentage, and carbohydrate grams for a large selection of generic and brand name food products and popular restaurant offerings. More detailed nutritional content includes sugar, fiber, calcium, iron, many vitamins, and related specifications. API methods support retrieval of nutritional information, either simple or detailed, for a generic product type or a name brand product available in the market. Methods also can return nutritional components by serving size.	medical,health,fitness,food,diet		http://www.programmableweb.com/api/tracknburn-fooddata	nutricion, food, calories, fat ercentage, carbohydrate grams, generic food, sugar, fiber, calcium, iron, vitamins, specifications	nutritional information, generic product type, brand name, XML	Medical
230	UniProt API	UniProt is a protein sequence and annotation database for the scientific community. The UniProt API offers RESTful access to all its resources and services.	medical,bioinformatics		http://www.programmableweb.com/api/uniprot	protein sequence	HTML,TEXT, GFF,XML,RDF,RSS	Medical
231	VIOLIN API	The Vaccine Investigation and Online Information Network (VIOLIN) is a central online resource for vaccine literature, vaccine research data, and data analyses for vaccines and vaccine candidates. It focuses on vaccines developed against pathogens considered to be of high priority in public health and biological safety. It's database includes research from studies using laboratory animals as well as humans. VIOLIN also provides a new program for vaccine target prediction. VIOLIN provides a set of programming utilities for accessing its database. Users may employ either SOAP or REST calls to retrieve entries on a specified pathogen or vaccine. All data is returned in XML format.	medical,Vaccination		http://www.programmableweb.com/api/violin	vaccine, data analysis for vaccines, pathogens	vaccine prediction, pathogen, vaccineXML	Medical

232	Pathways and Gene annotations for QTL region	This workflow searches for genes which reside in a QTL (Quantitative Trait Loci) region in the mouse, Mus musculus. The workflow requires an input of: a chromosome name or number; a QTL start base pair position; QTL end base pair position. Data is then extracted from BioMart to annotate each of the genes found in this region. The Entrez and UniProt identifiers are then sent to KEGG to obtain KEGG gene identifiers. The KEGG gene identifiers are then used to searcg for pathways in the KEGG pathway database.		adasd,chromosome,data-driven,disease,ensembl,entrez,gene,genes,genotype,kegg,mouse,nbiconworkflows,pathway,pathway-driven,pathways,phenotype,qtl,shim,subworkflow,uniprot	http://www.myexperiment.org/workflows/16.html	chromosome name ,start position ,end position	merged pathways ,gene descriptions ,pathway descriptions ,kegg pathway release ,report ,kegg external gene reference ,pathway ids ,genes pathways	Microarrays, Systems Biology

233	HUMAN Microarray CEL file to candidate pathways	This workflow takes in a CEL file and a normalisation method then returns a series of images/graphs which represent the same output obtained using the MADAT software package (MicroArray Data Analysis Tool) [http://www.bioinf.manchester.ac.uk/MADAT/index.html]. Also returned by this workflow are a list of the top differentialy expressed genes (size dependant on the number specified as input - geneNumber), which are then used to find the candidate pathways which may be influencing the observed changes in the microarray data. By identifying the candidate pathways, more detailed insights into the gene expression data can be obtained. NOTE - You will also need to install R and Rserv on your machine and install the libaries required by the R script into you R library directory (see for basic info: http://www.cs.man.ac.uk/~fisherp/rlib.html) The example inputs for this workflow are as follows: Samples1 = one or more CEL files for cross-correlating with Samples2 CEL files (new line separated including the .CEL): Liver Day1 Mouse.CEL Liver Day2 Mouse.CEL Samples2 = one or more CEL files for cross-correlating with Samples1 CEL files (new line separated including the .CEL): Kideny Day1 Mouse.CEL Kidney Day2 Mouse.CEL geneNumber = the number of differentialy expressed gene to be returned above a given p-value, e.g. 20 arrayTypeAffy = the name of the Mouse AffyMetrix array used, e.g. mouse4302, hgu133a... path = the direct path to the CEL file location, e.g. C:/Microarray Data/CEL FILES/ - note the forward slashes NormalizationMethod = the type of normalisation to perfrom, e.g. rma, gcrma or mmgmos testMethod = e.g. limma, mmtest or pplr p-value = the p-value cut-off value for the array data, e.g. 0.05 foldChange = the fold change value for the microarray data, e.g. 1 (means greater than 1 or less than -1)		affymetrix,cel,file,genotype,kegg,microarray,nbiconworkflows,pathway-driven,pathways,phenotype,shim,	http://www.myexperiment.org/workflows/10.html	arrayTypeAffy ,path ,NormalizationMethod ,testMethod ,pValue ,foldChange ,geneNumber ,samples1 ,samples2	hist ,boxNorm ,pca ,geneID ,pathway description ,merged pathways ,pathway list ,gene descriptions ,ensembl database release ,KEGG pathway release ,BioMart report	Microarrays, Systems Biology
234	Entrez Gene to KEGG Pathway	This workflow takes in Entrez gene ids then adds the string ncbi-geneid: to the start of each gene id. These gene ids are then cross-referenced to KEGG gene ids. Each KEGG gene id is then sent to the KEGG pathway database and its relevant pathways returned.		entrez,genotype,kegg,ncbi,pathway,pathway-driven,pathways,phenotype,shim	http://www.myexperiment.org/workflows/15.html	gene	merged kegg pathways	Microarrays, Systems Biology

235	Workflow for Protein Sequence Analysis	This workflow performs a generic protein sequence analysis. In order to do that a novel protein sequence enters into the software along with a list of known protein identifiers chosen by the biologist to perform a homology search, followed by a multiple sequence alignment and finally a phylogenetic analysis.		analysis,BLAST,homology,multiplesequencealignment,phylogenetictree,protein,sequence,tree,wpsa,	http://www.myexperiment.org/workflows/124.html	Input Sequence,ListUser,N-J or UPGMA	Blast Report ,Protein Description ,Image Alignment ,Output Tree (N or UPGMA) ,Distance Outfile ,Rooted Tree ,Unrooted Tree ,Not Protein Sequence ,It is a DNA or RNA sequence	Microarrays
236	Mapping OligoNucleotides to an assembly	The former version of the workflow expected that results from BioMART only report transcripts when the query (the probe in our,case) are entirely encapsulated in an exon of that transcript. However, the BioMart service also returns transcripts when the query is not or only partially overlapping with an exon in the stretch on the assembly on which a transcript is defined. This resulted in too many oligos classified as having multiple transcripts or having multiple genes.		biomart,BLAST,blat,ensembl,microarray,r,rshell	http://www.myexperiment.org/workflows/603.html	DataBaseName,Sequences	BlastReport, BioMartReport,SequencesNotFound,BarPlot,Classes,Report	Microarrays

237	BioAID ProteinDiscovery filterOnHumanUniprot perDoc html	This workflow finds proteins relevant to the query string via the following steps:,1) A user query: a single gene/protein name. E.g.: (EZH2 OR Enhancer of Zeste).,2) Retrieve documents: finds 'maximumNumberOfHits' relevant documents (abstract+title) based on query (the AIDA service inside is based on Apache's Lucene),3) Discover proteins: extract proteins discovered in the set of relevant abstracts with a 'named entity recognizer' trained on genomic terms using a Bayesian approach; the AIDA service inside is based on LingPipe. This subworkflow also 'filters' false positives from the discovered protein by requiring a discovery has a valid UniProt ID. Martijn Schuemie's service to do that contains only human UniProt IDs, which is why this workflow only works for human proteins.		AIDA,BioAID,bioassist nl,demo,nbiconworkflows,protein,text mining,text mining network,VL-e	http://www.myexperiment.org/workflows/154.html	query protein	discovery html table ,discovery table url ,discovery html ref	TextMining
238	EBI ClustalW 2	Perform a ClustalW multiple sequence alignment using the EBI's WSClustalW2 service. The set of sequences to align are the input, the other parameters for the search (see Job params) are allowed to default.		alignment,bioinformatics,clustal,clustalw,ebi,multiplesequencealignment,sequencealignment	http://www.myexperiment.org/workflows/203.html	Sequences,Email address	ClustalW alignment,ClustalW guide tree,Job ID,ClustalW output	Microarrays
239	Mapping microarray data onto metabolic pathways	This workflow maps microarray data onto metabolic pathway diagrams represented as SBML models drawn using Cell Designer. To run this workflow requires libsbml to be installed into taverna		beanshell,maxd, microarray,pathway,sbml	http://www.myexperiment.org/workflows/79.html		sbml ,image	Microarrays
240	EBI InterProScan for Taverna 2	Perform an InterProScan analysis of a protein sequence using the EBI's WSInterProScan service. The input sequence to use and the user e-mail address are inputs, the other parameters for the analysis (see Job params) are allowed to default.,InterProScan searches a protein sequence against the protein family and domain signature databases integrated into InterPro. A set of matches to the signatures are returned, which are annotated with the corresponding InterPro and GO term assignments for these signature matches.,		interpro,interproscan,looping,sequence,term	http://www.myexperiment.org/workflows/820.html	Email address,Sequence or ID	InterProScan text result ,InterProScan XML result ,Job ID ,InterProScan GFF ,status	Microarrays

241	Protein search fetch align tree	An implmentation of the classical sequence analysis workflow:,1) Find homologues (sequence similarity search),2) Fetch homologues,3) Align homologues (multiple sequence alignment),4) Produce phylogenetic tree,In this implementation the EBI webservices are used:,1) WU-BLAST (WSWUBlast) blastp vs. UniProtKB,2) dbfetch (WSDbfetch),3) ClustalW (WSClustalW2),4) ClustalW (WSClustalW2),		alignment,bioinformatics,BLAST,clustal,clustalw,dbfetch,ebi,multiplesequencealignment,neighbor-joining,phylogenetictree,protein,sequencealignment,sequencesimilaritysearch,tree,wu-blast	http://www.myexperiment.org/workflows/210.html	Email,Sequence or ID	SSS text,SSS XML,SSS Hit IDs,SSS job id,Hit sequences,MSA alignment,MSA job id,Phylogenetic tree text,Phylogenetic tree job id	Microarrays
242	Simplify a BLAST text file	This workflow simplifies a BLAST text file into identifiers, descriptions and values (P, E-values). In order to extract the relevant ids etc. you need to pass the relevant string into the corresponding port, e.g. the default port being used is gi. This has been passed gi. For any other ports simply pass in the string the SAME as the port name, e.g. seq id, p, per etc.		bioinformatics,BLAST,geneidentifier,gi,matrix,sequence,similarity,simple,simplifier,substitution,summary	http://www.myexperiment.org/workflows/22.html	blast file	simplified output	Microarrays
243	Liliopsida Protein Alignment	This workflow retrieves Liliopsida chloroplast petb gene sequences from NCBI Nucleotide, removes duplicate sequences and saves the results at BioExtract Server. These results are then converted into GenBank format and fed into Fetch Translation, which removes the translation from the CDS coding region. Translations are then used to build a multiple alignment using ClustalW.		alignment,bioextract,multiplesequencealignment,nucleotide,sequencealignment,alignment,bioextract,multiplesequencealignment,nucleotide,sequencealignment	http://www.myexperiment.org/workflows/550.html			Microarrays

244	KEGG pathways common to both QTL and microarray based investigations	This workflow takes in two lists of KEGG pathway ids. These are designed to come from pathways found from genes in a QTL (Quantitative Trait Loci) region, and from pathways found from genes differentially expressed in a microarray study. By identifying the intersecting pathways from both studies, a more informative picture is obtained of the candidate processes involved in the expression of a phenotype.		common,data-driven,disease,genotype,kegg,microarray,pathway,pathway-driven,pathways,phenotype,similarity	http://www.myexperiment.org/workflows/13.html	qtl pathways ,microarray pathways	intersecting pathways	Microarrays, Systems Biology
245	Mouse Microarray Analysis	This workflow takes in probesets from and AffyMetrix micorarray experiment and returns: the genes ; gene start and end positions; chromosome where genes reside; ensembl trasncripts; SwissProt ids; affy probeset identifiers for chips Mouse430 2 and Mouse430a 2.		affmetrix,cel,data-drive,disease,expression,file,gene,genotype,kegg,microarray,mouse,nbiconworkflows,pathway-driven,phenotype,RNA,shim,uniprot,	http://www.myexperiment.org/workflows/19.html	probeset list	merged pathways ,gene descriptions ,pathway descriptions ,ensembl database release ,kegg pathway release ,pathway list ,report	Microarrays
246	BioAID ProteinDiscovery	The workflow extracts protein names from documents retrieved from MedLine based on a user Query (cf Apache Lucene syntax). The protein names are filtered by checking if there exists a valid UniProt ID for the given protein name.		AIDA,BioAID,biorange nl,lucene,protein,proteins,pubmed,search,text mining,text mining network,uniprot,VL-e,	http://www.myexperiment.org/workflows/74.html	Query,maxHits parameter	Validated Protein,UniProtID	TextMining

247	Translate Nucleotide sequence into Peptide sequence	Translate Nucleotide sequence into Peptide sequence using EBI Service.,1) Find nucleotide sequence from EBI wsdbfetch(using ref.seq Id NM 005700),2) Translation nucleotide sequence(from 1) into peptide sequence using EBI EMBOSS4 transeq.,		pluripotency,scaffold,vector,virus	http://www.myexperiment.org/workflows/45.html		result sequence ,nucle sequence ,debug trans report ,debug detail status ,debug file	Microarrays
248	Fetch PDB flatfile from RCSB server	Given an identifier such as '1crn' fetches the PDB format flatfile and returns the corresponding 3D image of the protein.		bioinformatics,example,mygrid,pdb,protein,protein structure,rcsb,taverna	http://www.myexperiment.org/workflows/167.html	pdbID	pdbFlatFile	Microarrays
249	Perform a search through NCBI eUtils eSearch	This workflow takes in a search term and a database (e.g. snp, gene, protein) in which to perfom the search over. The result is an xml file containing summary information about the search term.,Example input for this workflow are given below:,database: pubmed,terms: cancer AND diabetes		esearch,eutils,ncbi,pubmed	http://www.myexperiment.org/workflows/25.html	terms ,database	xml output ,IdList	Microarrays
250	Multiple Blastp	This is a workflow to automate multiple BLASTp jobs on a large list of protein sequences in FASTA format.		bioinformatics,BLAST,protein,sequence	http://www.myexperiment.org/workflows/90.html	Protein sequences	Blast reports	Microarrays
251	Image Mining with RapidMiner	This is an image mining process using the image mining Web service provided by NHRF within e-Lico. It first uploads a set of images found in a directory, then preprocesses the images and visualizes the result. Furthermore, references to the uploaded images are stored in the local RapidMiner repository so they can later be used for further processing without uploading images a second time.		datamining,e-lico,imagemining,kup,rapidminer	http://www.myexperiment.org/workflows/1229.html			Microarrays

252	From cDNA Microarray Raw Data to Pathways and Published Abstracts	This workflow takes in a CDNA raw file and a normalisation method then returns a series of images/graphs which represent the same output obtained using the R and bioconductor. Also retruned by this workflow are a list of the top differentialy expressed genes (size dependant on the number specified as input - geneNumber), which are then used to find the candidate pathways which may be influencing the observed changes in the microarray data. By identifying the candidate pathways, more detailed insights into the gene expression data can be obtained. These pathways are subsequently used to obtain a corpus of published abstracts (from the PubMed database) relating to each biological pathway identified. These pathways are subsequently used to obtain a corpus of published abstracts (from the PubMed database) relating to each biological pathway identified. Also it generates a pie chart which, indicates the number of genes in a dataset that are regulated by a known transcriptional regulator, or by combination of regulators, and can suggest previously unknown regulatory interactions. The information for each regulon comes from files that are created manually from the EcoCyc database.		e.coli,kegg,KeggPathways,pathways,pubmed,systemsbiology	http://www.myexperiment.org/workflows/187.html	path ,pValue ,foldChange ,geneNumber ,regulonDir	Kegg gene desc ,merged pathway desc ,genes pathways ,pathway abstracts ,image ,boxNorm ,geneID ,prontTip ,pieChartRegulon ,PieList ,GeneList ,sizeFnr ,sizeArcA ,sizeGene ,geneIdwithoutEco	Microarrays, Systems Biology
253	GBSeq test	This workflow retrieves nucleotide and protein sequences with the literature and references associated to them given a protein and a nucleotide id.		bioinformatics,example,mygrid,ncbi,nucleotide,protein,retrieval,shim,taverna	http://www.myexperiment.org/workflows/168.html	nucleotide sequences,protein sequences,literature	protein xml ,nuc xml ,nuc tiny ,nuc fasta ,prot fasta ,prot insd ,nuc insd ,prot tiny	Sequence Analysis
254	Identification of differential genes using t-tests by R	This workflow starts by retrieving the names of microarray datasets from the Maxd database. The user has to select sets of control and test data for analysis using t-tests by R. A list of significant differentially expressed genes is then analysed using the Go Term Finder tool which generates a list of GO terms associated with the genes. A CSV file containing the list of significant genes is also generated.		beanshell,maxd,microarray,r,systemsbiology,user interaction	http://www.myexperiment.org/workflows/181.html		pdf,csv,spurious genes	Microarrays

255	Lymphoma type prediction based on microarray data	Scientific value Using gene-expression patterns associated with DLBCL and FL to predict the lymphoma type of an unknown sample. Using SVM (Support Vector Machine) to classify data, and predicting the tumor types of unknown examples. Steps Querying training data from experiments stored in caArray. Preprocessing, or normalize the microarray data. Adding training and testing data into SVM service to get classification result.		cabig,cagrid,cancerresearch,classification,geneexpression,globus,gridservice,lymphoma,lymphomaworkflow,microarray,supportvectormachine	http://www.myexperiment.org/workflows/746.html	EXPID	Result CSV	Microarrays
256	Identification of differential genes using the LIMMA Bioconductor package within R	This workflow starts by retrieving the names of microarray datasets from the Maxd database. The user has to select sets of control and test data which are then analysed by the LIMMA Bioconductor package in an R script. This produces a list of significant differentially expressed genes which is then analysed using the Go Term Finder tool to generate a PDF report of the common GO terms associated with the genes. A CSV file containing the list of significant genes is also generated.		bioconductor,limma,maxd,microarray,r,user interaction	http://www.myexperiment.org/workflows/246.html		pdf,csv,spurious genes	Microarrays

257	Protein alignmen t transme mbrane	Transmembrane domain prediction using EMBOSS tmap with an input sequence alignment of homolouges:,1) Sequence similarity search (SSS) to find homologues,2) Fetch sequences of hits,3) Multiple sequence alignment (MSA) of hit sequences,4) EMBOSS tmap with alignment from 3.,		alignment,bioinfo rmatics,clustal,cl ustalw,dbfetch,e bi,emboss,embos stmap,FASTA,mul tiplesequencealig nment,protein,pr oteinannotation,s equencealignmen t,sequencesimilar itysearch,transm embrane,transme mbranepredictio n,	http://www.myexpe riment.org/workflo ws/213.html	Email,Sequence or ID	SSS job ID,MSA job ID,tmap prediction,SS S hit IDs,MSA alignment,t map plot	Microarrays
258	Fasta string to fasta list	Split a string containing a set of sequences in fasta format into a list for fasta formated sequences.		bioinformatics,fa staformat	http://www.myexpe riment.org/workflo ws/228.html	fasta string	fasta list	Microarrays
259	Retrieve Protein Sequenc e	Retrieves a protein sequence in Fasta format from GenBank, given a GenBank identifier.,Example input for this workflow is:,EDL10223.1		bioinformatics,ne uroscience,newca stle,protein,sequ ence	http://www.myexpe riment.org/workflo ws/368.html	ID	protein sequence ,blast report	Microarrays
260	Downloa d pathway s for external referenc es list	Takes a list of external references to genes/proteins/metabolites, finds all pathways on WikiPathways that contain one of the given genes/proteins/metabolites and downloads them in a given file format.		pathway,pathway-driven,pathway-image,pathways, wikipathways	http://www.myexpe riment.org/workflo ws/393.html	xreflist,output path,file type	written files	Systems Biology

261	BioAID Discover proteins from text plus synonyms	This workflow discovers proteins from plain text and adds synonyms using Martijn Schuemie's proteins synonym service. Proteins are discovered with the AIDA 'Named Entity Recognize' web service by Sophia Katrenko (service based on LingPipe), from which output it filters out proteins. The Named Recognizer services uses the pre-learned genomics model, named 'MedLine', to find genomics concepts in plain text.		AIDA,BioAID,biorange nl,protein,synonyms,text mining,text mining network	http://www.myexperiment.org/workflows/81.html			TextMining
262	Get pathways by external reference	Finds pathways on WikiPathways by an external gene/protein/metabolite reference		pathway,pathway-driven,pathway-image,pathways,wikipathways	http://www.myexperiment.org/workflows/391.html	identifier,system code	pathway names,pathway species,pathway ids	Systems Biology
263	tRNAscan	Search a nucleotide sequence for tRNA genes using the tRNAscan-SE tool		bioinformatics,nucleotidesequence,nucleotidesequenceannotation,tRNA,trnascan,trnascan-se	http://www.myexperiment.org/workflows/245.html	Sequence or ID or GI Sequence origin	tRNAscan output	Microarrays
264	microRNA to KEGG Pathways and Abstracts	Workflow takes in a text file of microRNAs from microCOSM (at the EBI) and outputs a list of KEGG pathway information, including genes in pathways and pathway abstracts from PubMed. The results can then be used in various text mining applications/workflows to rank the results against a given disease.		bconv,beanshell,elico,ensembl,esearch,eutils,geneidentifier,geneid,genes,homosapiens,kegg,KeggID,microrna,ncbi,pathway,pathway-driven,pathways,pubmed,swissprot,textmining,uniprot	http://www.myexperiment.org/workflows/1138.html			TextMining

265	getInchIfromMassBankPeaklist ChemSpider workflow withImage	uses InChI's retrieved from a MassBank peaklist query to get compound information about those compounds via querying ChemSpider for information and displaying those results with image		accuratemass,bioinformatics,chemspider,compoundinfo+image,massspectrometry,massbank,metabolomics,systemsbiology	http://www.myexperiment.org/workflows/223.html	PeakList,SecurityToken	CompoundInfo,CompoundImage	Microarrays
266	Download pathways for external references list (Taverna 2)	Takes a list of external references to genes/proteins/metabolites, finds all pathways on WikiPathways that contain one of the given genes/proteins/metabolites and downloads them in a given file format.v		pathway,pathway-driven,pathway-image,pathways,wikipathways	http://www.myexperiment.org/workflows/642.html	xreflist ,output path ,file type	written files	Microarrays
267	Arabidopsis thaliana Microarray Analysis	This workflow searches for genes which are found to be differentially expressed in a microarray study using Arabidopsis thaliana. The workflow requires an input of a list of differentially expressed AffyMetrix Probeset identifiers. Data is then extracted from BioMart to annotate each of the genes. The UniProt identifiers are then sent to KEGG to obtain KEGG gene identifiers. The KEGG gene identifiers are then used to searcg for pathways in the KEGG pathway database.		affmetrix,affymetrix,arabidopsis,bconv,data-driven,ensembl,gene,geneexpression,geneid,genes,genotype,kegg,KeggPathways,microarray,pathway,pathways,phenotype,thaliana,uniprot	http://www.myexperiment.org/workflows/832.html	differentially expressed probesets	merged pathways ,gene descriptions ,pathway descriptions ,kegg pathway release ,kegg external gene reference ,pathway ids	Microarrays

268	EBI CENSOR	The CENSOR tool identifies and masks simple and complex sequence repeats found in nucleotide and protein sequences. This workflow uses the EBI's WSCensor web service		bioinformatics,censor,ebi,nucleotidesequence,protein,repeatmasking	http://www.myexperiment.org/workflows/244.html	Email,Sequence or ID,Repeat library	CENSOR job ID,CENSOR masked sequence,CENSOR alignment,CENSOR table	Microarrays
269	EBI IntAct	Find protein binary interactions using the EBI's IntAct service.		binary,bioinformatics,ebi,intact,protein,proteinannotation,proteininteraction	http://www.myexperiment.org/workflows/251.html	Query	IntAct result	Microarrays
270	Write pathway to disk	Downloads and writes a pathway from WikiPathways to a local disk in the given file type.		pathway,pathway-driven,pathway-image,pathways,wikipathways	http://www.myexperiment.org/workflows/392.html	file type,revision,filename,pathway id	writtenFile	Systems Biology
271	Kegg DrugID	This workflow accepts looks up drug identifiers from KEGG given a pathway identifier. You can enter a pathway ID in the form path:map07026		drug,example,kegg,pathway	http://www.myexperiment.org/workflows/730.html	pathway Id	Drug Id	Systems Biology
272	Construction of skeleton SBML model using subsystem term	This workflow generates a skeleton SBML model consisting of the metabolic reactions for a given subsystem term.		graph,image,model,sbml,systems biology,yeast	http://www.myexperiment.org/workflows/1198.html	subsystem term	sbml model,sbml graph	Models
273	get elements by pathway	Retrieve all objects on specified pathway get elements by pathway,Input example: path:bsu00010		elements,pathway	http://www.myexperiment.org/workflows/523.html	pathway id	result	Systems Biology

274	get drugs by pathways	Retrieves all drugs on the specified pathway. input example: path:map07025 ; path:eco00020		drug,pathway	http://www.myexperiment.org/workflows/520.html	pathway id	drug ids	Systems Biology
275	Kegg pathway diagrams	Find pathways in which all the genes in the list are involved. For each pathway draw the pathway diagram.,Find pathways in which all the genes in the list are involved. For each pathway draw the pathway diagram. Colour all enzyme boxes with colours specified. This workflow still has one problem. The list of colours have to be specified. I would like ideally to only except one background and one foreground colour and expand that to a list with length equivalent to the number of enzymes found - just duplicating the specified colours. However with almost no Taverna documentation to speak of, none of my efforts wanted to work so far.for each protein draw a diagram of the Kegg pathway that its protein is involved in and where available visualise the structures		colored,coloured,enzymes,genes,pathways	http://www.myexperiment.org/workflows/1120.html	gene ids,Species,bg color,fg color	KeggPathwayIDs,keggimageurl,keggGeneNames,coloured images,enzyme list,genes list	Systems Biology
276	metabolic pathway.xml	A list of Kegg entires are supplied to the Kegg database which then retrieves the associated metabolic pathways for each entry supplied. e.g. Ids takes in a value of 351, whilst abbr takes in a value of hsa. Thus hsa:351 corresponds to neurodegenerative disorders and alzheimers disease pathways.		benchmarks	http://www.myexperiment.org/workflows/299.html	Ids ,abbr	KEGG pathways	Systems Biology
277	Retrieve Pathways and Compound information from KEGG	Given a KEGG compound identifier (e.g. cpd:C00905), this workflow queries KEGG DB for pathways and compound information for each of these compounds. As the KEGG pathway service tries to find pathways which contain all input compounds, the input list is split up to circumvent this behaviour and to search for only one compound in a pathway at a time. Compounds identified in pathways are marked as red in the resulting pathway image.		bget,bioinformatics,pathway	http://www.myexperiment.org/workflows/738.html	bget in	KEGG bget ,KEGG pathways	Systems Biology
278	Get Gene Ids for Human	This workflow gets a list of gene ids (number depending on Ret Max value variable) for Homo sapiens. The species may be changed according to that desired, by altering the term value string constant		database,esearch,gene,geneidentifier,homosapiens,human,ncbi,species	http://www.myexperiment.org/workflows/1037.html		eSearch 2 output Count ,IdList	Microarrays

279	blastp using the MRS system	This blastp workflow uses the blast service of MRS (http://mrs.cmbi.ru.nl). Inputs are a sequence (only amino acids, not a fasta sequence) and a database name. Valid database names that can be used are sprot, uniprot, trembl, pdb, refseq, ipi and gpcrdb. Output is returned in XML.		BLAST,protein,similaritysearch,xml	http://www.myexperiment.org/workflows/581.html	sequence ,db	hits	Microarrays
280	Retrieve Pathways and Compound information from KEGG	Given a KEGG compound identifier (e.g. cpd:C00905), this workflow queries KEGG DB for pathways and compound information for each of these compounds. As the KEGG pathway service tries to find pathways which contain all input compounds, the input list is split up to circumvent this behaviour and to search for only one compound in a pathway at a time. Compounds identified in pathways are marked as red in the resulting pathway image.		bget,bioinformatics,pathway	http://www.myexperiment.org/workflows/738.html	bget in	KEGG bget ,KEGG pathways	Systems Biology
281	Kegg pathway diagrams	Find pathways in which all the genes in the list are involved. For each pathway draw the pathway diagram.,for each protein draw a diagram of the Kegg pathway that its protein is involved in and where available visualise the structures		gene,pathway,visualise	http://www.myexperiment.org/workflows/1118.html	gene ids,Species	attachmentlist,KeggPathwayIDs,attachmentlist2,KeggImage,keggimageurl,keggGeneNames	Systems Biology
282	caArray data retrieving	Query all the gene expression data in a caArray experiment. Returns a evenly divided gene expression data set with corresponding class information. They ca be later used as training and test data set in many classification algorithms.,Query all the gene expression data in a caArray experiment. Returns a evenly divided gene expression data set with corresponding class information. They can be later used as training and test data set in many classification algorithms.		caarray,cabig,cagrid,functionalworkflow,gene,globus,gridservice,microarray	http://www.myexperiment.org/workflows/963.html	EXP ID	testClass ,trainingClass ,trainingData ,testData	Microarrays

283	caArray data retrieving	Query all the gene expression data in a caArray experiment. Returns a evenly divided gene expression data set with corresponding class information. They ca be later used as training and test data set in many classification algorithms.,Query all the gene expression data in a caArray experiment. Returns a evenly divided gene expression data set with corresponding class information. They can be later used as training and test data set in many classification algorithms.		caarray,cabig,cagrid,functionalworkflow,gene,globus,gridservice,microarray	http://www.myexperiment.org/workflows/963.html	EXP ID	testClass,trainingClass,trainingData,testData	Microarrays
284	Parse unique proteins from Blast file	The workflow parses uses the blast results to determine the unique proteins found in the target genome that have no similairty to the source genome. Using these unique protein ids, and the original target protein fasta file, a fasta file of unique proteins is created.		protein	http://www.myexperiment.org/workflows/1184.html	blastFile,tfasta,cfasta file path		Microarrays
285	Calculating frequencies of gene expression levels using microarray data in MaxD	This workflow retrieves data from the MaxD microarray database and calculates the frequencies of gene expression levels using an R script		beanshell,database,expression,gene,image,maxd,microarray,r,xpath	http://www.myexperiment.org/workflows/1125.html			Microarrays
286	NCBI Gi to Kegg Pathway Descriptions	This workflow accepts a list of NCBI gene identifiers and returns descriptions of gene functions and a list of all pathways each gene is involved in (plus pathway image) from the KEGG database. This workflow replaces the earlier SOAP version with the new KEGG REST services		bconv,genbank,gene,genes,gi,kegg,ncbi,pathway,pathways	http://www.myexperiment.org/workflows/2659.html	genes	kegg mouseID,gene description,pathDescRest,pathwayID,PathwayImage	Systems Biology

287	UnigeneID to KEGG Pathways	This workflow accepts a list of Unigene gene identifiers and returns descriptions of gene functions and a list of all pathways each gene is involved in (plus pathway image) from the KEGG database. This workflow replaces the earlier SOAP version with the new KEGG REST services		bconv,gene,genes,kegg,pathway,pathways	http://www.myexperiment.org/workflows/2673.html	genes	geneList ,kegg mouseID ,gene description ,pathDescRest ,pathwayID ,PathwayImage	Systems Biology
288	gene ontology diagram.xml	This workflow builds up a subgraph of the Gene Ontology (http://www.geneontology.org) to show the context for a supplied term or terms. It shows this context by colouring all ancestors of the term, all children and all siblings. By default, ancestors of the supplied term or terms are coloured orange, siblings purple and direct children teal. Other terms appear in the default wheat colour.		benchmarks	http://www.myexperiment.org/workflows/317.html	termID	graphical	Microarrays
289	Gene to Pubmed	This workflow takes in a list of gene names and searches the PubMed database for corresponding articles. Any matches to the genes are then retrieved (abstracts only). These abstracts are then returned to the user.		abstracts,efetch,esearch,gene,gene identifier,geneid,genes,literature,medline,pubmed,text,textmining	http://www.myexperiment.org/workflows/1375.html	gene names	gene abstracts	Microarrays
290	Get Pathway-Genes and gene description by Entrez gene id	Given a specific entrez gene id, returns the pathways that this gene participates in and for each of those pathways which genes (including their description) are associated with.		entrez,gene,gene description,id,kegg,pathway,pathways	http://www.myexperiment.org/workflows/2843.html	entrez gene id	Pathway ,geneList ,gene description	Systems Biology

291	Match concept profiles with predefin ed set	Purpose of workflow: The workflow can be used to match a set of concept profiles with another set of concept profiles. The result is a list of concepts ordered by their match to the query concept profiles.,The workflow matches two sets of concept profiles. At the time of writing the concepts are derived from human, rat, and mouse terminologies, ontologies, and database identifiers. The profiles are lists of concepts ranked by their association with the identifying concept, as determined by co-location statistics computed from MedLine (up until 2009 at the time of writing).		annotation,association,concept,database,discovery,gene,GO,ontologies,similarity,statistics	http://www.myexperiment.org/workflows/3396.html	Cutoff,Match concept set,Query concept ID	Match concept ID,Similarity score	Microarrays
292	retrieve protein sequence and do a BLAST with options from DDBJ Web services	retrieve protein sequence and do a BLAST with options from DDBJ Web services		bioinformatics,BLAST,protein,sequence,services	http://www.myexperiment.org/workflows/1452.html	bioinformatics programs ,sequence database ,bioinformatics parameter ,UniProt accession	blast report ddbj	Microarrays
293	Find pathways in which two genes co-occur (in WikiPathways)	This workflow finds all pathways in which two gene symbols co-occur.,,This workflow was created as an exercise for the Managing and Integrating Information in the Life Sciences course 2013 at the LUMC, which is organized by the Netherlands Bioinformatics Center (NBIC).,		pathways	http://www.myexperiment.org/workflows/3636.html	Gene 1,Gene 2	pathway titles,pathway uris	Systems Biology

294	DAS sequence retrieval	Retrieve Protein or Genome sequences using the Distributed Annotation System (DAS).		das,genome,protein,sequence	http://www.myexperiment.org/workflows/2157.html	ID,DasSource,start,stop	Sequence DAS format,Service status	Microarrays
295	retrieve nucleotide sequence and do a high speed BLAST and extract position from DDBJ Web services	retrieve nucleotide sequence and do a high speed BLAST and extract position from DDBJ Web services		bioinformatics,BLAST,nucleotide,sequence,services	http://www.myexperiment.org/workflows/1455.html	bioinformatics program ,sequence database ,bioinformatics parameter ,DDBJ accession	position blast report ddbj	Microarrays

296	ADR-S	The ADR-S pathway seeks to establish a connection between the clinical event and the drug through different paths: (i) through proteins in common among the proteins that are drug targets or metabolite targets and proteins associated to the clinical event (ii) through proteins that are drug targets or metabolite targets and proteins associated to the clinical event that participate in a common biological pathway. The workflow proceeds as follows:,First, it checks if there are proteins that are annotated both to the clinical event and to the drug (NESTED WORKFLOW ADR substantiation through proteins).,Second, it looks for connections between the drug and the clinical event through biological pathways. Information about tissue expression of the proteins is used to filter the results Input of the workflow The input of the workflow is a drug-event pair. For the clinical events, the following types are allowed: 1) UMLS CUI concept identifiers (single identifier or a list of identifiers 2) clinical events observed as adverse drug reactions according to the EU-ADR project For the drug, a ATC code (7 digit level) is required. Output of the workflow As result a list of connecting proteins as well as a list of pathways is provided. The results can be visualized as a network using Cytoscape. The network is a multi-partite graph, in which the nodes are the event, the drug and the proteins, and the edges the associations between these nodes. In addition, all the evidences supporting the associations can be explored in the graph representation. The results of the analiysis through biological pathways is summarized in an html file.		adr,adversedrugreaction,bioinformatics,drugsafety,signal	http://www.myexperiment.org/workflows/1988.html	atc event eventType cytoscape eventName	drugTargets Output drugEventLinkingPathways eventProteinsOutput drugEventLinkingProteins CytoscapeResultGraph eventProteins drugTargets	Systems Biology
297	gene subset extract	This workflow functions for matching a set of genes as a part of whole gene data set and aim to extract the subset as a separate list.		gene,spreadsheet	http://www.myexperiment.org/workflows/1994.html	file url ,Gene ID	excel output ,split ,subset split ,matchedset ,single list	Microarrays

298	RAWverna Image evaluation - Read Image (RAW)	Reads an image from the given path using dcraw for image comparison.		beanshell,dcraw,image,raw,rawverna	http://www.myexperiment.org/workflows/2753.html	path	imageEquals ,imageOthers ,runtime	Imaging
299	Associated Region to Gene List	Produces a gene list from the Associated Region XML file		associatedgenes, gene	http://www.myexperiment.org/workflows/2728.html	additionalParameters,associatedSNPs	errors ,geneNames	Microarrays
300	Import and convert gene list	This workflow extracts a column of RefSeq gene IDs from a CSV file and then converts them to Unigene identifiers		ensembl,gene	http://www.myexperiment.org/workflows/2665.html	genes	Gene List	Microarrays
301	Drug Re-Purposing Workflow	The drug repurposing workflow system screens at least 20 bacterial proteomes against this set of proteins that are already being treated against using established drugs. By screening the bacterial proteomes it will be possible to find proteins of highly similar structure to those that are existing drug protein targets and so this will infer that it is highly likely that the drugs can be used as antimicrobials against these proteins of highly similar structure. Proteomes that will be screened belong to Gram positive bacteria, with special focus on Staphylococcus aureus. We look at a variety of species and strains as the company advised. A mixture of screening targets could potentially identify targets for broad-spectrum antimicrobial development. The drug repurposing workflow shows where in the KEGG pathways the proteins are active and therefore where the action of the drug will be in the biochemical pathways of the bacteria. The workflow will update the Ondex database where new information on Gene Ontology annotations, functional annotation and literature references are available. All information about each unique protein is made available in an HTML document.		BLAST,FASTA,protein,proteins,proteome,similarity,uniqueness	http://www.myexperiment.org/workflows/1984.html	Drug Target Protein FASTA ,Target Proteomes GI ,root directory ,thresholdIdentity	protein ids ,NCBIGitoKeggPathways pathway descriptions ,GO output 2 ,NCBIGitoKeggPathways URL ,InterPro ,PubMed Results 2 ,out ,out 2	Systems Biology

302	Gene To Pathways (Vistrails)	This workflow retrieves the pathways that are associated with given Kegg gene identifiers.		geneidentifier,kegg,pathway	http://www.myexperiment.org/workflows/3064.html			Systems Biology
303	retrieve protein sequence and do a BLAST and extract position from DDBJ Web services	retrieve protein sequence and do a BLAST and extract position from DDBJ Web services		bioinformatics,BLAST,ddbj,protein,services,xml	http://www.myexperiment.org/workflows/1450.html	sequence database ,bioinformatics program ,UniProt accession	position blast report ddbj	Microarrays
304	RAWverna Image Evaluator - Evaluate relative	Takes two images as byte array and compares them using the RAWverna Image Evaluator.		comparison,image,javaimageevaluator,raw,rawverna	http://www.myexperiment.org/workflows/2757.html	image	runtime,evaluator object image similarity	Imaging
305	Retrieve a protein from the GPCRDB	This small workflow illustrates how to use the web service access provided by the GPCRDB in Taverna.,The proteinId input field is case sensitive and by default the identifiers in the GPCRDB are lowercase. You can try this mini-workflow with e.a. 'adrb2 human'.		gpcrdb,protein	http://www.myexperiment.org/workflows/1484.html	proteinId	results	Microarrays

306	Extract unique proteins from blast results	The workflow parses uses the tab-delimited BLAST results to determine the unique proteins found in the target genome that have no similarity to the source genome.The workflow parses uses the blast results to determine the unique proteins found in the target genome that have no similairty to the source genome. Using these unique protein ids, and the original target protein fasta file, a fasta file of unique proteins is created.This workflow allows you to configure a BioMart query to fetch sequences you want from Ensembl. These sequences are retrieved and a blast database of them is created (by default, in the directory you ran taverna from). Warning: This workflow assumes that you have blastall and formatdb installed on the machine, and that by default, these are both found or linked in /usr/local/bin. It also assumes that you have write permission to the directory you have run taverna from. The beanshells create blastall cmdArgs and create formatdb cmdArgs are what you need to edit if the default locations are not appropriate for you. Shortcomings: The names of all the files created and used is hard coded in this workflow. This means that if you run this workflow more than once without editing anything, you will overwrite files you have previously created. All files created in the working directory are not yet coded to be deleted via the workflow. Ideally there would be an option that a user could choose that would set the files to be kept or deleted after use.Workflow outputs a list of proteins encoded by the target genomes that do not have sequences similarity to those encoded by the source genome		BLAST,FASTA,protein,proteins,proteome,similarity,tab,uniqueness	http://www.myexperiment.org/workflows/1981.html	blastFile,tfasta	unique identifiers,blasted identifiers	Microarrays

307	GeneTalk	GeneTalk, a web-based platform, that can filter, reduce and prioritize human sequence variants from NGS data and assist in the time consuming and costly interpretation of personal variants in clinical context. It serves as an expert exchange platform for clinicians and scientists who are searching for information about specific sequence variants and connects them to share and exchange expertise on variants that are potentially disease-relevant.		Genetic variation annotation,Sequence variation analysis,Variant Calling,Structural variation discovery,Filtering,Annotation,Database,Exome analysis,Sequence analysis,Variant Classification,Viewer	http://seqanswers.com/wiki/GeneTalk	VCF	VCF,XLS,XLSX	Sequence Analysis
308	MethMarker	MethMarker facilitates the design of DNA methylation assays for COBRA, bisulfite SNuPE, bisulfite pyrosequencing, MethyLight and MSP. It also implements a systematic workflow for design, optimization and (computational) validation of DNA methylation biomarkers. This workflow starts from a preselected differentially methylated region (DMR) and results in an optimized DNA methylation assay that is ready to be tested in a large-scale clinical trial.	Epigenomics, DNA methylation		http://seqanswers.com/wiki/MethMarker			Sequence Analysis
309	Omixon Variant Toolkit	Omixon Target Standard, Target HLA and Target Pro are designed to help clinical, diagnostic and research labs to efficiently get the maximum accuracy and precision from their targeted NGS data.	Comparative genomics,Mapping,Sequence analysis,Read alignment,InDel discovery,SNP discovery	Alignment,Assembly,Mapping,Colorspace,Basespace	http://seqanswers.com/wiki/Omixon_Variant_Toolkit	FASTQ,CSFASTA,SAM,most others	SAM,VCF,GFF	Sequence Analysis

310	Syapse	Syapse is a platform and application suite for bringing together omics and clinical data.	[illegible] transcription, DNA methylation, DNA-Seq,Exome and Whole genome variant detection,Exome and whole genome variant detection,InDel discovery,New gene discovery,SNP discovery,Structural variation discovery,RNA-Seq,Small RNA,Small RNA transcriptome,Cancer biology,ChIP-Seq,Comparative genomics,Comparative	Biological Contextualization,Differentially expressed gene identification,Exome analysis,LIMS,Integrated Solution,Sample Barcoding,Sequence analysis,Variant Classification,Variant Prioritization,Visualization,Workflow,Filtering,Gene ontology,Gene ontology analysis,Genetic variation annotation	http://seqanswers.com/wiki/Syapse	VCF		clinical, omics	Sequence Analysis
311	BING	biomedical informatics pipeline (BING) for the analysis of NGS data that offers several novel computational approaches to 1. image alignment, 2. signal correlation, compensation, separation, and pixel-based cluster registration, 3. signal measurement and base calling, 4. quality control and accuracy measurement.		Basecaller,Sequencing Quality Control	http://seqanswers.com/wiki/BING				Sequence Analysis

312	SeqGSEA	Gene Set Enrichment Analysis (GSEA) of RNA-Seq Data: integrating differential expression and splicing	Biomedical Sciences,Genomics,RNA-Seq	Statistics,Functional analysis,Gene set enrichment analysis	http://seqanswers.com/wiki/SeqGSEA	BAM,SAM	Tab separated	Sequence Analysis
313	SequenceVariant Analyzer	DNA sequence information underpins genetic research, enabling discoversies of important biological or medical benefit. Compared with previous discovery strategies, a whole-genome sequencing study is no longer constrained by differing patterns of linkage disequilibrium, thus, in theory, is more possible to directly identify the gentic variants contributing to biological traits or medical outcomes.,The rapidly evolving high-throughput DNA sequencing technologies have now allowed the fast generation of large amount of sequence data for the purpose of performing such whole-genome sequencing studies, at a reasonable cost. SequenceVariantAnalyzer, or SVA, is a software tool that we have been developing to analyze the genetic variants identified from such studies.,URL: http://www.svaproject.org/	Personal genomics,Genomics,Sequence analysis	Annotation,Genetic variation annotation,Genome browser	http://seqanswers.com/wiki/SequenceVariantAnalyzer	SAMtools,HMMCNV	sva	Sequence Analysis

314	Geneious	Search, organize and analyze genomic and protein information of any size via desktop program that provides publication ready images to enhance the impact of your research.	Phylogenetics,Sequence analysis,De-novo assembly,Whole Genome Resequencing,Alignment,Systems biology,Comparative genomics,SNP discovery,InDel discovery,Transcription Factor analysis,Genomics,Population genetics,Homology,Metagenomics,Read alignment,Structural variation,RNA-Seq,Motif analysis	Alignment,Assembly,Assembly validation,Annotation,Multiple sequence alignment viewer,Motif analysis,Genetic variation annotation,Basecaller,Genome browser,Sample Barcoding,Database,Mapping,Visualization	http://seqanswers.com/wiki/Geneious	FASTA,FASTQ,GenBank,SAM,Illumina,ELAND,CSFASTA/CSQUAL (ABI SOLiD)		Sequence Analysis,Systems Biology
315	ABMapper	Maps RNA-Seq reads to target genome considering possible multiple mapping locations and splice junctions	Genomics,Transcriptomics	Mapping,Alignment	http://seqanswers.com/wiki/ABMapper	SAM	SAM,BED	Sequence Analysis
316	ABBA	Assembly Boosted By Amino acid sequence is a comparative gene assembler, which uses amino acid sequences from predicted proteins to help build a better assembly	Genomic Assembly	Assembly,Scaffolding	http://seqanswers.com/wiki/ABBA			Sequence Analysis

317	ALEXA-Seq	Alternative Expression Analysis by massively parallel RNA sequencing	RNA-Seq Quantitation, Alternative Splicing		http://seqanswers.com/wiki/ALEXA-Seq			Sequence Analysis
318	ANNOVAR	ANNOVAR: Functional annotation of genetic variants from high-throughput sequencing data	Genomics,Genetics	Annotation,Variant Prioritization	http://seqanswers.com/wiki/ANNOVAR			Sequence Analysis
319	Arf	arf is a genetic analysis program for sequencing data.			http://seqanswers.com/wiki/Arf			Sequence Analysis
320	Array Suite (Array Studio/Server)	Array Studio is a complete analysis and visualization package for NextGen sequencing data, as well as other -OMIC data types. Array Server is a backend enterprise server for storage and analysis of -OMIC and NextGen sequencing data.	Genomics,SNP discovery,InDel discovery	Mapping,Expression profiling	http://seqanswers.com/wiki/Array_Suite_%28Array_Studio/Server%29			Sequence Analysis
321	ArrayExpressHTS	R-based pipeline for RNA-Seq data analysis.	RNA-Seq,RNA-Seq Quantitation		http://seqanswers.com/wiki/ArrayExpressHTS	FASTQ		Sequence Analysis
322	ArrayStar	ArrayStar is an easy-to-use gene expression analysis software package that offers powerful visualization and statistical tools to help you analyze your microarray data.	Gene Expression Analysis	Differentially expressed gene identification,Gene ontology analysis,Sequence variation analysis,Statistics	http://seqanswers.com/wiki/ArrayStar			Sequence Analysis,Microarrays
323	ASC	Empirical Bayes method to detect differential expression.	RNA-Seq Quantitation	Empirical Bayes	http://seqanswers.com/wiki/ASC			Sequence Analysis
324	ATAC	ATAC is a computational process for comparative mapping between two genome assemblies, or between two different genomes.		Assembly validation,Alignment	http://seqanswers.com/wiki/ATAC	FASTA	Custom	Sequence Analysis

325	Avadis NGS	Avadis NGS is a desktop software platform for alignment, analysis, visualization, and management of data generated by next-generation sequencing (NGS) platforms. It supports workflows for RNA-Seq, DNA-Seq, and ChIP-Seq analysis and is designed with the biologist in mind.	ChIP-Seq,DNA-Seq,RNA-Seq,Small RNA,Pathway analysis	Alignment,Quality Control,Sequence analysis,Visualization,Biological Contextualization	http://seqanswers.com/wiki/Avadis_NGS	SAM,BAM,BED,ELAND,FASTA,FASTQ		Sequence Analysis
326	Batman	Bayesian tool for methylation analysis (Batman) €"for analyzing methylated DNA immunoprecipitation (MeDIP) profiles	DNA methylation		http://seqanswers.com/wiki/Batman	GFF		Sequence Analysis
327	BaySeq	Identify differential expressed genes	RNA-Seq Quantitation	Differentially expressed gene identification	http://seqanswers.com/wiki/BaySeq			Sequence Analysis
328	BBSeq	Tool for analyzing RNA-Seq data to analyze gene expression	RNA-Seq Quantitation		http://seqanswers.com/wiki/BBSeq			Sequence Analysis
329	CNANorm	A normalization method for Copy Number Aberration in cancer samples.	Cancer biology,Copy number estimation,Genomics	Mixture model,Peak detection,Normalization	http://seqanswers.com/wiki/CNANorm	Delimited Text,SAM,BAM	Delimited Text	Sequence Analysis
330	BEDTools	BEDTools is an extensive suite of utilities for comparing genomic features in BED format.	Genomics	Mapping	http://seqanswers.com/wiki/BEDTools	BED,BAM,GFF,VCF	BED,BAM,GFF,VCF	Sequence Analysis
331	BFCounter	BFCounter is a program for counting k-mers in DNA sequence data.		K-mer analysis	http://seqanswers.com/wiki/BFCounter	FASTQ,(Compressed) FASTQ		Sequence Analysis
332	Biopieces	The Biopieces are a collection of bioinformatics tools that can be pieced together in a very easy and flexible manner to perform both simple and complex tasks. The Biopieces work on a data stream in such a way that the data stream can be passed through several different Biopieces, each performing one specific task: modifying or adding records to the data stream, creating plots, or uploading data to databases and web services.	Genomics	Alignment,Quality Control,Sequence analysis,Visualization	http://seqanswers.com/wiki/Biopieces			Sequence Analysis

333	BiQ Analyzer	BiQ Analyzer is a software tool for easy visualization and quality control of DNA methylation data. With more than 2,000 downloads so far, BiQ Analyzer has become a standard tool for processing DNA methylation data from bisulfite sequencing.	Epigenomics, DNA methylation		http://seqanswers.com/wiki/BiQ_Analyzer			Sequence Analysis
334	Bismark	Bismark is a tool to map bisulfite treated sequencing reads and perform methylation calling in a quick and easy-to-use fashion.	Epigenomics, Genomics,DNA methylation	Bisulfite mapping,Mapping,Methylation Calling	http://seqanswers.com/wiki/Bismark	FASTQ,FASTA	SAM (or custom)	Sequence Analysis
335	BRCA-diagnostic	Computational screening test for BRCA1/2 mutants in human genomic DNA	Personal genomics		http://seqanswers.com/wiki/BRCA-diagnostic	FASTQ		Sequence Analysis
336	Breakway	Breakway is a suite of programs that take aligned genomic data and report structural variation breakpoints.	Whole Genome Resequencing,Genomics,Structural variation,InDel discovery	Sequence analysis,Genetic variation annotation	http://seqanswers.com/wiki/Breakway	BAM	TXT	Sequence Analysis
337	CABOG	Celera Assembler is scientific software for DNA research.	De-novo assembly	Assembly	http://seqanswers.com/wiki/CABOG	FASTA,QUAL,SFF,AFG,Trace Archive		Sequence Analysis
338	CGA Tools	Tools for viewing, manipulating and converting data from Complete Genomics		Conversion	http://seqanswers.com/wiki/CGA_Tools		SAM	Sequence Analysis

339	Chipster	User-friendly NGS data analysis software with built-in genome browser and workflow functionality. Chipster includes tools for ChIP-seq, RNA-seq, miRNA-seq and MeDIP-seq analysis, and functionality for exome-seq and CGH-seq will soon be added.	ChIP-Seq,RNA-Seq,MiRNA-Seq,MeDIP-Seq	QC,Filtering,Trimming,Mapping,Peak calling,Motif detection,Differential expression,Pathway analysis,Methylation analysis,Genomic region matching,Genome browser	http://seqanswers.com/wiki/Chipster	FASTQ,SAM,BAM,BED,GTF	FASTQ,SAM, BAM,BED,GTF	Sequence Analysis
340	CLCbio Genomics Workbench	De novo and reference assembly SNP and small indel detection and annotation.	Genomics,Whole Genome Resequencing,De-novo assembly,SNP discovery,InDel discovery,ChIP-Seq,RNA-Seq,MiRNA,Transcriptomics	Mapping,Assembly,Alignment,Colorspace,BLAST,Ab-inito gene prediction,Adapter Removal (software),Annotation,Assembly QC,Basespace,Bisulfite SNP calling,De Bruijn graph,Heatmaps	http://seqanswers.com/wiki/CLCbio_Genomics_Workbench	FASTA,FASTQ,GenBank,SAM,BAM,Illumina Bustard,ELAND,CSFASTA/CSQUAL (ABI SOLiD)	FASTA,FASTQ,GFF,GenBank,SAM,BAM,ACE,Nexus,CSV,PDF,XLS	Sequence Analysis
341	CleaveLand	A pipeline for using degradome data to find cleaved small RNA targets.	MiRNA		http://seqanswers.com/wiki/CleaveLand	BED,FASTA		Sequence Analysis
342	CNANorm	A normalization method for Copy Number Aberration in cancer samples.	Cancer biology,Copy number estimation,Genomics	Mixture model,Peak detection,Normalization	http://seqanswers.com/wiki/CNANorm	Delimited Text,SAM,BAM	Delimited Text	Sequence Analysis

343	ConDeTri	ConDeTri is a content dependent read trimming software for Illumina/Solexa sequencing data	RNA-Seq,DNA-Seq,Genomics	Trimming	http://seqanswers.com/wiki/ConDeTri	FASTQ	FASTQ	Sequence Analysis
344	CPTRA	Integrated transcriptome analysis from Sanger, 454, Solexa, SOLiD, etc reads	RNA-Seq Alignment,RNA-Seq Quantitation		http://seqanswers.com/wiki/CPTRA			Sequence Analysis
345	CRAC	CRAC is a mapping software specialized for RNA-Seq data. It detects mutations, indels, splice or fusion junctions in each single read.	Mapping,RNA Seq analysis,RNA-Seq Alignment,Alternative Splicing,Fusion genes,Fusion transcripts,SNP discovery,InDel discovery	Mapping,Read mapping,Burrows-Wheeler,FM-Index	http://seqanswers.com/wiki/CRAC	(C)FAST(A/Q)	SAM (or custom)	Sequence Analysis
346	Cufflinks	Cufflinks assembles transcripts and estimates their abundances in RNA-Seq samples. It accepts aligned RNA-Seq reads and assembles the alignments into a parsimonious set of transcripts. Cufflinks then estimates the relative abundances of these transcripts based on how many reads support each one.	RNA-Seq Alignment,RNA-Seq Quantitation,Differential Expression,Alternative Splicing,Transcriptome assembly,RNA-Seq	Transcript assembly,Mapping,Differentially expressed gene identification,Differential expression	http://seqanswers.com/wiki/Cufflinks	SAM	GTF	Sequence Analysis

347	CummeRbund	Allows for persistent storage, access, exploration, and manipulation of Cufflinks high-throughput sequencing data. In addition, provides numerous plotting functions for commonly used visualizations.	RNA-Seq Quantitation	Visualization	http://seqanswers.com/wiki/CummeRbund			Sequence Analysis
348	DeFuse	deFuse is a software package for gene fusion discovery using RNA-Seq data. The software uses clusters of discordant paired end alignments to inform a split read alignment analysis for finding fusion boundaries. The software also employs a number of heuristic filters in an attempt to reduce the number of false positives and produces a fully annotated output for each predicted fusion	Fusion genes,RNA-Seq,Fusion transcripts		http://seqanswers.com/wiki/DeFuse	FASTQ		Sequence Analysis
349	DEGseq	an R package to identify differentially expressed genes or isoforms for RNA-seq data from different samples	RNA-Seq Quantitation	Differentially expressed gene identification	http://seqanswers.com/wiki/DEGseq			Sequence Analysis
350	DESeq	DESeq is an R package to analyse count data from high-throughput sequencing assays such as RNA-Seq and test for differential expression.	RNA-Seq Quantitation, ChIP-Seq	Statistical testing,Sequencing Quality Control	http://seqanswers.com/wiki/DESeq	table with count data	table	Sequence Analysis
351	DIAL	A computational pipeline for identifying single-base substitutions between two closely related genomes without the help of a reference genome.	SNP discovery,Comparative genomics		http://seqanswers.com/wiki/DIAL			Sequence Analysis
352	DNA Chromatogram Explorer	DNA Chromatogram Explorer is a Windows Explorer clone dedicated to DNA sequence analysis and manipulation.		Chromatogram management,Chromatogram viewer,Conversion	http://seqanswers.com/wiki/DNA_Chromatogram_Explorer	DNA sequence, FASTA,SCF,ABI,GenBank,SEQ,TXT	DNA sequence analysis, FASTA,SCF,SEQ,TXT	Sequence Analysis
353	DNAA	DNAA (DNA Analysis) software for analysis of Next-Generation Sequencing data.	Structural variation,SNP discovery,DNA methylation	Statistics,Sequencing Quality Control,Simulation	http://seqanswers.com/wiki/DNAA	SAM,BAM	DNA analysis	Sequence Analysis
354	DSAP	Automated multiple-task web service designed to provide a total solution to analyzing deep-sequencing small RNA datasets generated by next-generation sequencing technology	Small RNA transcriptome,MiRNA		http://seqanswers.com/wiki/DSAP	sequence tag file (read count,sequence)	small RNA sequencing	Sequence Analysis

355	DSGseq	This program aims to identify differentially spliced genes from two groups of RNA-seq samples.	RNA-Seq,Differential Expression,Alternative Splicing	RNA-Seq analysis,Differential expression,Alternative Splicing,Statistical testing	http://seqanswers.com/wiki/DSGseq	SAM/BAM,Table with count data	Delimited Text	Sequence Analysis
356	E-miR	Perl tools for processing miRNA sequencing data	Small RNA transcriptome,MiRNA		http://seqanswers.com/wiki/E-miR	small RNA, miRNA	miRNA sequencing	Sequence Analysis
357	EdgeR	edgeR is an R/Bioconductor software package for statistical analysis of replicated count data. Methods are designed for assessing differential expression in comparative RNA-Seq experiments, but are generally applicable to count data from other genome-scale platforms (ChIP-Seq, MeDIP-Seq, Tag-Seq, SAGE-Seq etc).	RNA-Seq,RNA-Seq Quantitation, ChIP-Seq,Gene Expression Analysis,DNA methylation	Statistical testing	http://seqanswers.com/wiki/EdgeR	RNA-Seq, genome, Table with count data	Table	Sequence Analysis
358	Epigenome	A bioinformatic pipeline that scores epigenetic alterations according to strength and significance and links them to potentially affected genes.	Epigenomics	Bisulfite mapping	http://seqanswers.com/wiki/Epigenome	genes	epigenetic alterations	Sequence Analysis
359	EpiGRAPH	EpiGRAPH enables biologists to analyze genome and epigenome datasets with powerful statistical and machine learning methods. In a typical workflow, the user uploads a set of genomic regions of interest (e.g. experimentally mapped enhancers, hotspots of epigenetic regulation or sites exhibiting disease-specific alterations), and EpiGRAPH searches a large database of (epi-) genomic attributes for significant overlap and correlation with the regions in the input dataset. Furthermore, EpiGRAPH can predict the status of genomic regions that were not included in the input dataset.	Epigenomics	Statistics,Machine Learning	http://seqanswers.com/wiki/EpiGRAPH	genome, set of genomic regions, disease specific alterations	genome analysis, epigenome datasets	Sequence Analysis

360	ERANGE	ERANGE is a Python package for doing RNA-seq and ChIP-seq.	RNA-Seq Alignment,RNA-Seq Quantitation, ChIP-Seq,Allele-specific transcription		http://seqanswers.com/wiki/ERANGE	RNA-Seq, ChIP-seq	RNA-Seq, ChIP-seq	Sequence Analysis	

361	ERGO Genome Analysis and Discovery System	ERGO provides a systems-biology informatics toolkit centered on comparative genomics to capture, query and visualize sequenced genomes. Building upon the most comprehensive genomic database available anywhere integrated with the largest collection of microbial metabolic and non-metabolic pathways and using Igenbio's proprietary algorithms, ERGO assigns functions to genes, integrates genes into pathways, and identifies previously unknown or mischaracterized genes, cryptic pathways and gene products.	[illegible] reconstruction,Phylogenetics,Comparative genomics,SNP Annotation,SNP discovery,Alignment,Exome analysis,Metagenomics,Pathway analysis,Comparative transcriptomics,Functional Genomics,Gene Expression Analysis,Genome Wide Association Studies,Fusion finding,Fusion genes,Fusion transcripts,Sequence annotation,Sequence		http://seqanswers.com/wiki/ERGO_Genome_Analysis_and_Discovery_System	genomics, genomes, genome sequence	visualize sequenced genomes, collection of microbial metabolic pathways, collection of non-metabolic pathways, identify mischaracterized genes, cryptic pathways, gene products	Sequence Analysis
362	FDM	Detects differential transcription in RNA-Seq data	RNA-Seq Quantitation		http://seqanswers.com/wiki/FDM	RNA-Seq, SAM	differential transcription	Sequence Analysis

363	Filter	Produces a filtered version of an sRNA dataset, controlled by several user-defined criteria, including sequence length, abundance, complexity, transfer and ribosomal RNA removal.	General bioinformatics (pipeline)	Filtering	http://seqanswers.com/wiki/Filter	FASTA,FASTQ	FASTA	Sequence Analysis
364	Flux	FluxCapacitor s a computer program to predict splice form abundancies from reads of an RNA-seq experiment. FluxSimulator can generate simulated data for testing RNA-seq pipelines	RNA-Seq	Simulation	http://seqanswers.com/wiki/Flux	RNA-Seq	RNA-Seq	Sequence Analysis
365	FragGeneScan	Application for finding (fragmented) genes in short reads	Metagenomics		http://seqanswers.com/wiki/FragGeneScan	FASTA	FASTA,tab separated	Sequence Analysis
366	FreClu	a frequency-based, de novo short-read clustering method that organizes erroneous short sequences originating in a single abundant sequence into a tree structure; in this structure, each “child” sequence is considered to be stochastically derived from its more abundant “parent” sequence with one mutation through sequencing errors. The root node is the most frequently observed sequence that represents all erroneous reads in the entire tree, allowing the alignment of the reliable representative read to the genome without the risk of mapping erroneous reads to false-positive positions.	RNA-Seq Alignment	Mapping	http://seqanswers.com/wiki/FreClu	RNA-Seq	RNA-Seq alignment	Sequence Analysis
367	Freebayes	Bayesian genetic variant detector (SNPs, indels, MNPs)	Genomics		http://seqanswers.com/wiki/Freebayes	BAM	VCF	Sequence Analysis
368	FusionCatcher	FusionCatcher searches for novel/known fusion genes, translocations, and chimeras in RNA-seq data (paired-end reads from Illumina NGS platforms like Solexa and HiSeq).	RNA-Seq,Fusion finding	Alignment	http://seqanswers.com/wiki/FusionCatcher	FASTQ,(Compressed) FASTQ,SRA	text	Sequence Analysis
369	FusionMap	Detects fusion events in both single- and paired-end datasets from either RNA-Seq or gDNA-Seq studies and characterize fusion junctions at base-pair resolution.	Fusion genes,Fusion transcripts	Split-read	http://seqanswers.com/wiki/FusionMap	FASTA,FASTQ	SAM	Sequence Analysis
370	FusionSeq	Identifies fusion transcripts from paired end RNA-Seq data.	Fusion transcripts,RNA-Seq,Fusion genes	Alignment Analysis	http://seqanswers.com/wiki/FusionSeq	MRF	GFR,BP	Sequence Analysis
371	G-Mo.R-Seq	G-Mo.R-Se is a method aimed at using RNA-Seq short reads to build de novo gene models.	RNA-Seq Alignment		http://seqanswers.com/wiki/G-Mo.R-Seq			Sequence Analysis

372	GATK	The Genome Analysis Toolkit (GATK) is a structured programming framework designed to enable rapid development of efficient and robust analysis tools for next-generation DNA sequencers. The GATK solves the data management challenge by separating data access patterns from analysis algorithms, using the functional programming philosophy of Map/Reduce	SNP discovery	MapReduce,Programming Library,Localized reassembly/realignment	http://seqanswers.com/wiki/GATK	genome, DNA sequences, SAM	genome analysis, VCF	Sequence Analysis
373	Gbrowse	Genome Viewer		Visualization	http://seqanswers.com/wiki/Gbrowse	genome	genome view	Sequence Analysis
374	GENE-Counter	GENE-counter is a computational pipeline for analyzing RNA-Sequencing (RNA-Seq) data for differential gene expression	RNA-Seq		http://seqanswers.com/wiki/GENE-Counter	gene,FASTA	RNA-Sequencing analysis, gene expression	Sequence Analysis
375	GeneProf	GeneProf is a web-based, graphical software suite and database resource for high-throughput-sequencing experiments (RNA-seq and ChIP-seq).	Functional Genomics,RNA-Seq,ChIP-Seq	Workflow,Quality Control,Alignment,Visualization,Peak finding,Differentially expressed gene identification	http://seqanswers.com/wiki/GeneProf	FASTA,(Compressed) FASTA,(Compressed) FASTQ,Tab-delimited,Table with count data,Others	Images,XLS,TXT,FASTA,FASTQ,BED,WIG,Tab-delimited,HTML	Sequence Analysis
376	Genomatix Mining Station (GMS)	The Genomatix Mining Station (GMS) offers mapping of NGS reads onto genomes, transcriptomes and splice-junction libraries. It is a client-server based solution and can be controlled through an intuitive GUI or via command-line. It covers different tasks such as, as genomic positioning, SNP detection, splice analyses and genomic enrichments.	RNA-Seq,SNP discovery,ChIP-Seq	Assembly,Mapping,SNP calling,Genomic correlations	http://seqanswers.com/wiki/Genomatix_Mining_Station_%28GMS%29	solexa,Solexa with Probability,FASTA,FASTQ,EMBL,GenBank	FASTA,BED,SAM,BAM,GAF	Sequence Analysis

377	Genome Browse	A free genome browser for exploring sequencing pile-up and coverage data with numerous annotation tracks hosted on the cloud.	Sequence analysis,DNA-Seq,Alignment,De novo sequencing,Exome analysis,Exome and whole genome variant detection,Genetics,Whole Genome Resequencing,Next Generation Sequencing,Genomics	Alignment viewer,Assembly visualization,Visualization	http://seqanswers.com/wiki/GenomeBrowse	genome, BAM	genome sequencing	Sequence Analysis
378	Genome Jack	GenomeJack is a genome browser specialized in next-generation sequencing data. Advantages are intuitive interface and smooth drag'n drop response.	Genomics,Personal genomics	Visualization	http://seqanswers.com/wiki/GenomeJack	genome, BAM,GTF,GFF,BED,BEDGraph,WIG,VCF,TSV,FASTA	genome sequencing	Sequence Analysis
379	Genometa	Genometa is a Java based local bioinformatics program which allows rapid analysis of metagenomic short read datasets. Millions of short reads can be accurately analysed within minutes and visualised in the browser component. A large database of diverse bacteria and archaea has been constructed as a reference sequence.	Metagenomics,Genomics	Mapping,Visualization	http://seqanswers.com/wiki/Genometa	FASTA,FASTQ	SAM/BAM,Delimited Text	Sequence Analysis
380	Genome Tools	The GenomeTools genome analysis system is a free collection of bioinformatics tools for genome informatics.1.3.6	Genomics		http://seqanswers.com/wiki/GenomeTools	genome	genome analysis	Sequence Analysis

381	Genome View	GenomeView is a next-generation stand-alone genome browser and editor initiated in the BSB group at VIB and currently developed at Broad Institute. It provides interactive visualization of sequences, annotation, multiple alignments, syntenic mappings, short read alignments and more. Many standard file formats are supported and new functionality can be added using a plugin system.	Genomics,Comparative genomics,Comparative transcriptomics,Transcriptomics,Gene annotation retrieval,Quality Control,Sequencing,Sequence analysis	Visualization,Alignment viewer,Multiple sequence alignment viewer,Viewer,Genome browser	http://seqanswers.com/wiki/GenomeView	genome, (Compressed) FASTA,BAM,BED,BEDGraph,Blast output,Clustalw,EMBL,FASTA,GFF,GTF,Multi-fasta,WIG	genome view, sequence visualization, multiple alignments, syntenic mappings, gff3,embl	Sequence Analysis
382	GenVision	GenVision is a genomic visualization software package that is fully integrated with Lasergene and is designed to support easy generation of publication quality graphics and maps.	Genomics	Visualization	http://seqanswers.com/wiki/GenVision	genomic	genomic visualization	Sequence Analysis
383	GMAP	GMAP (Genomic Mapping and Alignment Program) for mRNA and EST Sequences.		Alignment,Mapping	http://seqanswers.com/wiki/GMAP	(Compressed) FASTQ	SAM (or custom)	Sequence Analysis
384	Golden Helix	Golden Helix is a bioinformatic software provider and analytic service provider. The core of its business is about empowering scientists to discover more, discover it easier, and to come away with valid and reproducible bioinformatics results. The software, SNP & Variation Suite, is a stable platform for clever data manipulations, robust quality assurance, advanced statistical modeling, and compelling visual results in a genome browser environment of DNA Seq, Copy Number variation, SNP Chip, and RNA Seq data.	Epigenomics, Genomics,DNA-Seq,SNP discovery,Whole Genome Resequencing Analysis,Copy number estimation,Quality Control	Quality Control,Statistics, Statistical testing,Genome browser,Annotation,Filtering,Collapsing Methods,Variant Classification,Variant Mapping	http://seqanswers.com/wiki/Golden_Helix	epigenomics, genomics, DNA-Seq, genome	DNA-Seq, SNP discovery, genome resequencing analysis	Sequence Analysis

385	Goseq	An R package to detect Gene Ontology (GO) categories and other categories of genes (such as KEGG pathways) that are over/under represented in an RNA-seq data.	RNA-Seq Quantitation	Gene Set Testing	http://seqanswers.com/wiki/Goseq	genes	RNA-Seq	Sequence Analysis
386	Gowinda	Gowinda: unbiased analysis of gene set enrichment for Genome Wide Association Studies	Genomics,Genome Wide Association Studies,Population genetics,Population Genomics,High-throughput sequencing	Gene set enrichment,Gene ontology,Genome wide association studys	http://seqanswers.com/wiki/Gowinda	GTF,VCF,Pileup	gene analysis	Sequence Analysis
387	GPSeq	Analyze RNA-seq data to estimate gene and exon expression, identify differentially expressed genes, and differentially spliced exons	RNA-Seq Quantitation		http://seqanswers.com/wiki/GPSeq		RNA-Seq analysis, identify differentialy expressed genes, differentially spliced exons	Sequence Analysis
388	Hairpin Annotation	Generates a secondary structure from an RNA sequence and highlights regions of interest using RNAplot	General bioinformatics (pipeline)		http://seqanswers.com/wiki/Hairpin_Annotation	Delimited Text	Various image file formats	Sequence Analysis
389	IGV	The Integrative Genomics Viewer (IGV) is a high-performance visualization tool for interactive exploration of large, integrated datasets. It supports a wide variety of data types and format, including short-read alignments in the SAM/BAM format. Data can be viewed from local files or over the web via http.	Genomics	Visualization	http://seqanswers.com/wiki/IGV	BAM,SAM,GOBY,BED,GFF,GTF,PSL,CN,GCT,FASTA	genomic view	Sequence Analysis

390	Ingenuity Variant Analysis	Ingenuity Variant Analysis is a web application that helps researchers studying human disease to identify causal variants from human resequencing data in just minutes. Ingenuity Variant Analysis combines analytical tools and integrated content to help you rapidly identify and prioritize variants by drilling down to a small, targeted subset of compelling variants based both upon published biological evidence and your own knowledge of disease biology. With Variant Analysis, you can interrogate your variants from multiple biological perspectives, explore different biological hypotheses, and identify the most promising variants for follow-up.	Genomics,Genetics,Next Generation Sequencing,Exome and Whole genome variant detection,Whole Genome Resequencing Analysis,Exome analysis,Causal Variant Detection,Targeted Sequencing	Biological Interpretation and Analysis of DNA Sequence Data	http://seqanswers.com/wiki/Ingenuity_Variant_Analysis	genomics, genetics, sequencing, exome, whole genome resequencing	genome resequencing analysis, exome analysis, DNA sequence analysis	Sequence Analysis
391	Integrated Genome Browser	Visualization software for next-generation genomics	Genomics	Visualization	http://seqanswers.com/wiki/Integrated_Genome_Browser	BAM,BED,PSL,GFF	various image file formats	Sequence Analysis

392	IOmics	iOmics is a cloud based workflow analysis framework for managing, analyzing and visualizing NGS data	Genomics,Transcriptomics,Epigenomics,RNA-Seq,Exome and Whole genome variant detection	Genome Alignment,Assembly,Ab-inito gene prediction,Genetic variation annotation,Exome analysis,ChIP seq,MiRNA analysis (Ref and Ab-initio)	http://seqanswers.com/wiki/Iomics	genomics, epigenomics, RNA-Seq	NGS data visualization	Sequence Analysis
393	Kissnp	kisSnp compares two sets of NGS raw reads, detecting Single Nucleotide Polymorphism occurring between the two sets. The two sets typically come from the sequencing of two individuals from the same species or from closely related species.	Comparative genomics,Comparative transcriptomics,Gene annotation retrieval,SNP discovery,InDel discovery	Micro assembly,De Bruijn graph	http://seqanswers.com/wiki/Kissnp	FASTA	FASTA	Sequence Analysis
394	LASTZ	A tool for (1) aligning two DNA sequences, and (2) inferring appropriate scoring parameters automatically	Genomics	Mapping,Alignment	http://seqanswers.com/wiki/LASTZ	FASTA,HSX,others	LAV,AXT,MAF,SAM,CIGAR,others	Sequence Analysis
395	MapDamage	Identifies and quantifies DNA damage patterns in ancient DNA	Ancient DNA,DNA-Seq	Quality Control,Statistical Modelling	http://seqanswers.com/wiki/MapDamage	SAM,BAM		Sequence Analysis

396	MeV	Visualization of genomic data, Differential Gene Expression based on DEGseq, DESeq and edgeR	RNA-Seq	Clustering,Visualization,Classification,Differentially expressed gene identification	http://seqanswers.com/wiki/MeV		genomic data visualization, differential gene expression	Sequence Analysis
397	MiRanalyzer	Web-server for identifying and analyzing miRNA in next-gen sequencing experiments	MiRNA		http://seqanswers.com/wiki/MiRanalyzer		miRNA analysis	Sequence Analysis
398	MiRCat	Predicts mature miRNAs and their precursors from an sRNA dataset and a genome.	General bioinformatics (pipeline)	MiRNA Prediction	http://seqanswers.com/wiki/MiRCat	FASTA,FASTQ	csv,txt,FASTA,Various image file formats	Sequence Analysis
399	MiRDeep	Discovering known and novel miRNAs from deep sequencing data	MiRNA		http://seqanswers.com/wiki/MiRDeep		miRNA sequencing data	Sequence Analysis
400	MiRNAkey	A software pipeline for the analysis of microRNA Deep Sequencing data	MiRNA		http://seqanswers.com/wiki/MiRNAkey	FASTQ,FASTA	TXT	Sequence Analysis
401	MiRProf	Determines normalised expression levels of sRNAs matching known miRNAs in miRBase.	General bioinformatics (pipeline)	MiRNA profiling	http://seqanswers.com/wiki/MiRProf	FASTA,FASTQ	CSV	Sequence Analysis
402	MirTools	Web server for microRNA profiling and discovery based on high-throughput sequencing	Small RNA transcriptome,MiRNA		http://seqanswers.com/wiki/MirTools	Short read tag with count	Detailed annotation	Sequence Analysis
403	MMSEQ	Pipeline and methodology for simultaneously estimating isoform expression and allelic imbalance in diploid organisms using RNA-seq data.	Allele-specific transcription		http://seqanswers.com/wiki/MMSEQ	RNA-Seq data, FASTQ	allele transcription	Sequence Analysis
404	MochiView	Hybrid genome browser and motif visualization/analysis/management desktop software.	Genomics,ChIP-Seq,ChIP-on-chip,RNA-Seq,Motif analysis	Genome browser,Motif analysis	http://seqanswers.com/wiki/MochiView	Custom,WIG,BED,GFF,FASTA,MEME,Bioprospector,custom Motif format	Varied	Sequence Analysis

405	MUMmer	MUMmer is a modular system for the rapid whole genome alignment of finished or draft sequence. Basically it is a ultra-fast alignment of large-scale DNA and protein sequences	Genomics,Transcriptomics	Alignment,Mapping	http://seqanswers.com/wiki/MUMmer	FASTA	delta	Sequence Analysis
406	Mutascope	Mutascope is a software suite designed to analyze data from high throughput sequencing of PCR amplicons, with an emphasis on normal-tumor comparison for the accurate and sensitive identification of low prevalence mutations.	Cancer biology	Somatic variant calling,Analysis Pipeline	http://seqanswers.com/wiki/Mutascope	FASTQ,SAM/BAM	.bam,.vcf,other	Sequence Analysis
407	MuTect	MuTect is a method developed at the Broad Institute for the reliable and accurate identification of somatic point mutations in next generation sequencing data of cancer genomes.		SNP calling	http://seqanswers.com/wiki/MuTect	cancer genomes	cancer genomes mutations	Sequence Analysis
408	Myrna	Myrna is a cloud computing tool for calculating differential gene expression in large RNA-seq datasets.	RNA-Seq Quantitation, RNA-Seq Alignment	Hadoop,MapReduce	http://seqanswers.com/wiki/Myrna	RNA-Seq dataset	differential gene expression	Sequence Analysis
409	Olego	OLego is a program specifically designed for de novo spliced mapping of mRNA-seq reads. OLego adopts a seeding and extension scheme, and does not rely on a separate external mapper. It achieves high sensitivity of junction detection by using very small seeds (12-14 nt), efficiently mapped using Burrows-Wheeler transform (BWT) and FM-index. This also makes it particularly sensitive for discovering small exons. It is implemented in C++ with full support of multiple threading, to allow fast processing of large-scale data.	Genomics,RNA-Seq,RNA-Seq Alignment	Mapping,Alignment	http://seqanswers.com/wiki/Olego	FASTA,FASTQ	SAM,BED	Sequence Analysis

410	PALMA	We present a novel approach based on large margin learning that combines accurate splice site predictions with common sequence alignment techniques. By solving a convex optimization problem, our algorithm -- called PALMA -- tunes the parameters of the model such that true alignments score higher than other alignments. We study the accuracy of alignments of mRNAs containing artificially generated micro-exons to genomic DNA. In a carefully designed experiment, we show that our algorithm accurately identifies the intron boundaries as well as boundaries of the optimal local alignment. It outperforms all other methods: for 5702 artificially shortened EST sequences from C. elegans and human it correctly identifies the intron boundaries in all except two cases. The best other method is a recently proposed method called exalin which misaligns 37 of the sequences. Our method also demonstrates robustness to mutations, insertions and deletions, retaining accuracy even at high noise levels.	RNA-Seq Alignment	Alignment	http://seqanswers.com/wiki/PALMA		RNA alignment	Sequence Analysis
411	Partek Genomics Suite	Easy to use software providing A to Z analysis for all Next Generation Sequencing and Microarray data.	Allele-specific transcription, RNA-Seq Quantitation, Epigenomics, Functional Genomics,ChIP-Seq,Alternative Splicing,SNP discovery,Small RNA transcriptome		http://seqanswers.com/wiki/Partek_Genomics_Suite	sequencing	microarray data	Sequence Analysis,Microarrays
412	PatMaN	Patman searches for short patterns in large DNA databases, allowing for approximate matches. It is optimized for searching for many small pattern at the same time, for example microarray probes.		Mapping	http://seqanswers.com/wiki/PatMaN	DNA data	DNA matching, mapping	Sequence Analysis,Microarrays

413	Pipeline Pilot	Analysis and workflow development of Next Generation Sequencing and gene expression.	Next Generation Sequencing,Gene expression,Sequence analysis,SNP discovery	General bioinformatics,Mapping,De-novo assembly,Sequence analysis,Variant detection,Gene expression analysis,RNA-Seq analysis,ChIP-Seq analysis,Genomics,Comparative genomics,Whole genome resequencing,Sequence alignment	http://seqanswers.com/wiki/Pipeline_Pilot	Bowtie,BreakDancerMax,BWA,CisGenome,CNV-seq,Cufflinks,FASTQC,GATK,Integrative Genomics Viewer,MACS,Mapreads,MIRA3,MUMmer,SAMtools,snpEff,Tablet,TopHat,Velvet,tabix,Artemis,BioJava,BioPerl,BLAST,ClustalW,EMBOSS,HMMER,Jalview,MUSCLE,primer3,PROSITE,sim4	Circos,GBrowse2	Sequence Analysis
414	Qseq	QSeq is DNASTAR's Next-Gen application for RNA-Seq,ChIP-Seq, and miRNA alignment and analysis.	ChIP-Seq,RNA-Seq,MiRNA	Integrated Solution,Alignment,Visualization,Protein Binding Peak Detection	http://seqanswers.com/wiki/Qseq	RNA-Seq, ChIP-seq, miRNA alignment	alignment, analysis	Sequence Analysis
415	Rdiff	rDiff is an open source tool for accurate detection of differential RNA processing from RNA-Seq data. It implements two statistical tests to detect changes of the RNA processing between two samples. rDiff.parametric is a powerful test, which can be applied for well annotated organisms to detect changes in the relative abundance of isoforms. rDiff.nonparametric is an alternative when the annotation is incomplete or missing.	RNA-Seq Alignment,Differential RNA processing regulation,Alternative Splicing,RNA-Seq,Transcriptomics	Statistical testing	http://seqanswers.com/wiki/Rdiff	SAM/BAM,GFF3	Tab-delimited file	Sequence Analysis

416	Contra	Copy number analysis for exome-sequencing / targeted-resequencing. Two methods of analysis available: Case vs Control, or Case vs Baseline. Function available for creating a baseline from multiple samples.	Next Generation Sequencing,Cancer biology,Genomics,Copy number estimation		http://seqanswers.com/wiki/Contra	BAM,BED	VCF,Tab-delimited	Sequence Analysis
417	Bis-SNP	BisSNP is a package based on the Genome Analysis Toolkit (GATK) map-reduce framework for genotyping in bisulfite treated massively parallel sequencing (Bisulfite-seq, NOMe-seq and RRBS) on Illumina platform. It uses bayesian inference with either manually specified or automatically estimated methylation probabilities of different cytosine context(not only CpG, CHH, CHG in Bisulfite-seq, but also GCH et.al. in other bisulfite treated sequencing) to determine genotypes and methylation levels simultaneously.	SNP discovery,Genotyping,DNA methylation, Bisulfite Sequencing	Bisulfite SNP calling,Methylation Calling,MapReduce	http://seqanswers.com/wiki/Bis-SNP	BAM	VCF,BED,BEDGRAPH,WIG	Sequence Analysis
418	Bison	Bison allows users with access to a computer cluster to rapidly align whole-genome bisulfite sequencing or RRBS reads. It can align both directional and non-directional libraries and uses bowtie2.	Epigenomics, Bisulfite Sequencing,DNA methylation	Bisulfite mapping,Mapping,Methylation Calling	http://seqanswers.com/wiki/Bison	(Compressed) FASTQ,FASTQ	BAM,BEDGRAPH	Sequence Analysis
419	BRAT	accurate and efficient tool for mapping short reads obtained from the Illumina Genome Analyzer following sodium bisulfite conversion. Both single and paired ends are supported.,We present a new, accurate and efficient tool for mapping short reads obtained from the Illumina Genome Analyzer following sodium bisulfite conversion. Our tool, BRAT, supports single and paired-end reads and handles input files containing reads and mates of different lengths. BRAT is faster, maps more unique paired-end reads and has higher accuracy than existing programs. The software package includes tools to end-trim low quality bases of the reads and to report nucleotide counts for mapped reads on the reference genome.	Epigenomics, DNA methylation	Bisulfite mapping,Mapping	http://seqanswers.com/wiki/BRAT	genome, sodium bisulfite conversion	genome analysis	Sequence Analysis

420	BSMAP	short reads mapping software for bisulfite sequencing,Bisulfite sequencing is a powerful technique to study DNA cytosine methylation. Bisulfite treatment followed by PCR amplification specifically converts unmethylated cytosines to thymine. Coupled with next generation sequencing technology, it is able to detect the methylation status of every cytosine in the genome. However, mapping high-throughput bisulfite reads to the reference genome remains a great challenge due to the increased searching space, reduced complexity of bisulfite sequence, asymmetric cytosine to thymine alignments, and multiple CpG heterogeneous methylation. We developed an efficient bisulfite reads mapping algorithm BSMAP to address the above issues. BSMAP combines genome hashing and bitwise masking to achieve fast and accurate bisulfite mapping. Compared with existing bisulfite mapping approaches, BSMAP is faster, more sensitive and more flexible. BSMAP is the first general-purpose bisulfite mapping software. It is able to map high-throughput bisulfite reads at whole genome level with feasible memory and CPU usage.	DNA methylation	Mapping,Bisulfite mapping	http://seqanswers.com/wiki/BSMAP	FASTA,FASTQ	SOAP mapping format	Sequence Analysis
421	BSSim	BSSim: Bisulfite sequencing simulator for next-generation sequencing.	DNA methylation, Bisulfite Sequencing	Simulation	http://seqanswers.com/wiki/BSSim	FASTA	FASTQ,SAM, Ref	Sequence Analysis
422	BiQ Analyzer HT	BiQ Analyzer HT is an enhanced version of BiQ Analyzer that provides extensive support for high-throughput bisulfite sequencing. BiQ Analyzer HT facilitates the processing, quality control and initial analysis of single-basepair resolution DNA methylation data. It was developed for deep bisulfite sequencing of one or more loci using the Roche 454 platform, but it easily extends to other sequencing platforms. BiQ Analyzer HT features a biologist-friendly graphical user interface, a fast alignment algorithm and a variety of ways to visualize DNA methylation data.	Epigenomics, DNA methylation, Bisulfite Sequencing			DNA methylation	bisulfite sequencing	Sequence Analysis

423	GSNAP	GSNAP can align both single-end and paired-end reads as short as 14 nt and of arbitrarily long length. It can detect short- and long-distance splicing, including interchromosomal splicing, in individual reads using probabilistic models or a database of known splice sites. Our program also permits SNP-tolerant alignment to a reference space of all possible combinations of major and minor alleles, and can align reads from bisulfite treated DNA for the study of methylation state.	RNA-Seq Alignment,DNA methylation	Mapping,Bisulfite mapping	http://seqanswers.com/wiki/GSNAP	RNA-Seq Alignment,DNA methylation	Mapping,Bisulfite mapping	Sequence Analysis
424	Kismeth	Web-based tool for bisulfite sequencing analysis	DNA methylation, Epigenomics	Bisulfite mapping	http://seqanswers.com/wiki/Kismeth	DNA methylation,Epigenomics	Bisulfite mapping	Sequence Analysis
425	Methpipe	The MethPipe software package is a computational pipeline for analyzing bisulfite sequencing data (BS-seq, WGBS and RRBS). MethPipe provides tools for mapping bisulfite sequencing read and estimating methylation levels at individual cytosine sites. Additionally, MethPipe includes tools for identifying higher-level methylation features, such as hypo-methylated regions (HMR), partially methylated domains (PMD), hyper-methylated regions (HyperMR), and allele-specific methylated regions (AMR).	Epigenomics, DNA methylation, Bisulfite Sequencing,High-level methylation feature,epigenetic domains	Bisulfite mapping,Hidden Markov Model,Workflow	http://seqanswers.com/wiki/Methpipe	bisulfite sequence, FASTQ,SAM/BAM	Bisulfite mapping	Sequence Analysis
426	MethylCoder	Pipeline for fast, simple processing of BiSulfite-treated reads into methylation data. Includes scripts for analysis and visualization. In addition to a binary output, the direct output of methylcoder is a text file that indicates per-nucleotide methylation context (CG/CHG/CHH) and methylation levels (both coverage and C-T conversions),Pipeline for fast, simple processing of BiSulfite-treated reads into methylation data. Includes scripts for analysis and visualization. In addition to a binary output, the direct output of methylcoder is a text file that indicates per-nucleotide methylation context (CG/CHG/CHH) and methylation levels (both coverage and C-T conversions). The direct output of methylcoder is a text file that looks like	Genomics,Sequencing,DNA methylation, Epigenomics	Mapping,Bisulfite mapping	http://seqanswers.com/wiki/MethylCoder	FASTQ,FASTA	binary,TXT,SAM	Sequence Analysis
427	MetMap	Produces corrected site-specific methylation states from MethylSeq experiments and annotates unmethylated islands across the genome.	DNA methylation		http://seqanswers.com/wiki/MetMap	genome	DNA methylation	Sequence Analysis

428	PASH	Pash 3.0 performs sequence comparison and read mapping and can be employed as a module within diverse configurable analysis pipelines, including ChIP-Seq and methylome mapping by whole-genome bisulfite sequencing	Epigenomics, DNA methylation	Alignment,Bisulfite mapping	http://seqanswers.com/wiki/PASH	Epigenomics,DNA methylation	Alignment,Bisulfite mapping	Sequence Analysis
429	RMAP	Assembles 20 - 64 bp Solexa reads to a FASTA reference genome. By Andrew D. Smith and Zhenyu Xuan at CSHL. (published in BMC Bioinformatics). POSIX OS required.	DNA methylation	Mapping,Bisulfite mapping	http://seqanswers.com/wiki/RMAP	genome	Mapping,Bisulfite mapping	Sequence Analysis
430	Sherman	bisulfite-treated Read FastQ Simulator	Genomics,Bisulfite Sequencing,DNA methylation	Simulation	http://seqanswers.com/wiki/Sherman	Genomics,Bisulfite Sequencing,DNA methylation	FASTQ,CSFASTA/CSQUAL (ABI SOLiD)	Sequence Analysis
431	SOCS	SOLiD reference based, un-gapped alignment with bisulfite capability	RNA-Seq Alignment,DNA methylation, SNP discovery	Mapping,Bisulfite mapping	http://seqanswers.com/wiki/SOCS	CSFASTA/CSQUAL (ABI SOLiD)	Delimited Text	Sequence Analysis
432	Mapsembler	Mapsembler is a targeted assembly software. It takes as input a set of NGS raw reads and a set of input sequences (starters). It first determines if each starter is read-coherent, e.g. whether reads confirm the presence of each starter in the original sequence. Then for each read-coherent starter, Mapsembler outputs its sequence neighborhood as a linear sequence or as a graph, depending on the user choice.	Metagenomics,Transcriptomics,DNA-Seq,RNA-Seq Quantitation, Targeted assembly	Assembly,micro assembly,Mapping	http://seqanswers.com/wiki/Mapsembler	FASTA	FASTA,XGMML (Cytoscape), graphml (Gephi)	Sequence Analysis

433	Spiral Genetics	Spiral Genetics provides alignment to reference, variant detection, variant filtering and annotation for any sized next generation sequencing dataset. Using cloud computing, the Spiral Platform can produce results at ultra high speeds through a web browser interface. No computing infrastructure required.	Alignment,Comparative genomics,DNA-Seq,Exome and Whole genome variant detection,Genomic Assembly,Integrated solution,Mapping,Quality Control,Read alignment,Reference assembly,Resequencing,SNP Annotation,SNP discovery,Sequence analysis,Sequencing,Targeted assembly,Targeted resequencing,Whole Genome Resequencing	Alignment,Annotation,Assembly,Burrows-Wheeler,T-Map,Data compression,Genome Alignment,Hadoop,MapReduce,Mapping,Trimming	http://seqanswers.com/wiki/Spiral_Genetics	Fastq,Fastq.gz,Fastq.bz,qseq,qseq.gz,qseq.bz	Tab-delimited file,VCF,SAM	Sequence Analysis
434	ZINBA	Identifies genomic regions enriched in a variety of ChIP-seq and related next-generation sequencing experiments	ChIP-Seq,DNA-Seq		http://seqanswers.com/wiki/ZINBA	genomic, BED	ChIP-Seq, sequencing	Sequence Analysis
435	BS Seeker	Mapping tool for bisulfite treated reads	Epigenomics	Bisulfite mapping	http://seqanswers.com/wiki/BS_Seeker	Epigenomics	Bisulfite mapping	Sequence Analysis

436	BS-Seq	The source code and data for the Shotgun Bisulphite Sequencing of the Arabidopsis Genome Reveals DNA Methylation Patterning Nature paper by Cokus et al. (Steve Jacobsen's lab at UCLA). POSIX.	Epigenomics	Bisulfite mapping	http://seqanswers.com/wiki/BS-Seq	genome, DNA methylation	Bisulfite mapping	Sequence Analysis
437	ChromHMM	ChromHMM is software for learning and characterizing chromatin states.	Epigenomics	Hidden Markov Model,Segmentation	http://seqanswers.com/wiki/ChromHMM		chromatin states	Sequence Analysis
438	Gimme Motifs	GimmeMotifs is a de novo motif prediction pipeline, especially suited for ChIP-seq datasets. It incorporates several existing motif prediction algorithms in an ensemble method to predict motifs and clusters these motifs using the WIC similarity scoring metric.	Transcription regulation,ChIP-Seq,Epigenomics	Motif analysis	http://seqanswers.com/wiki/GimmeMotifs	BED,FASTA	PSSM,HTML	Sequence Analysis
439	Hicup	A mapping pipeline for HiC interaction data. Performs independent mapping on each end of the interaction pair and removes commonly found artefacts.	Epigenomics	Mapping	http://seqanswers.com/wiki/Hicup	FASTQ,(Compressed) FASTQ	SAM/BAM	Sequence Analysis
440	NPS	Identify nucleosome positions given histone-modification ChIP-seq or nucleosome sequencing at the nucleosome level.	Epigenomics, ChIP-Seq		http://seqanswers.com/wiki/NPS	ChIP-seq, nuclosome sequencing	nuclosome positions	Sequence Analysis
441	NucleR	nucleR is a R/Bioconductor package for working with tiling arrays and next generation sequencing. It uses a novel aproach in this field which comprises a deep profile cleaning using Fourier Transform and peak scoring for a quick and flexible nucleosome calling	ChIP-on-chip,ChIP-Seq,Nucleosome Positioning,Epigenomics	Annotation,Peak calling	http://seqanswers.com/wiki/NucleR	ShortRead,BioConductor	WIG,BED,BioConductor	Sequence Analysis
442	RSAT peak-motifs	A workflow combining a series of time- and memory-efficient motif analysis tools to extract motifs from full-size collections of peaks as generated by ChIP-seq, ChIP-chip or other ChIP-X technologies.	ChIP-Seq,regulatory genomics,epigenomics	motif discovery,motif scanning,motif comparison	http://seqanswers.com/wiki/RSAT_peak-motifs	Fasta	HTML,text,graphics (png)	Sequence Analysis

443	SeqMonk	A tool to visualise and analyse high throughput mapped sequence data SeqMonk is designed to be an easy to use application for the analysis of large scale mapped sequence datasets. It allows you to visualise your mapped datasets against an annotated genome with very flexible visualisation options.,In addition you can also quantitate your data in a number of different ways, and then filter these results to pull out regions of interest. Finally you can annotate regions of interest and export reports and figures for further analysis.,SeqMonk can be used to analyse a wide range of data types, ChIP-Seq, Genome Reseqencing, 3/4C, MeDIP, Bisulphite Seq etc.	Genomics,Epigenomics	Visualization,Assembly visualization,statistical testing,Alignment viewer	http://seqanswers.com/wiki/SeqMonk	Eland,GFF,BED,Maq,Bowtie,SAM,BAM,Delimited Text,Bismark	PNG,SVG,Delimited Text	Sequence Analysis
444	SICER	A clustering approach for identification of enriched domains from histone modification ChIP-Seq data.	ChIP-Seq,Epigenomics	Filtering	http://seqanswers.com/wiki/SICER	BED	WIG	Sequence Analysis
445	ExomeCopy	CNV detection from exome sequencing read depth	Exome and Whole genome variant detection,Copy number estimation,Exome analysis	Hidden Markov Model	http://seqanswers.com/wiki/ExomeCopy	BAM		Sequence Analysis
446	RUbioSeq	RUbioSeq has been developed to facilitate the primary and secondary analysis of resequencing projects by providing an integrated software suite of parallelized pipelines to detect exome variants (SNVs and CNVs) and to perform Bisulfite-seq analyses automatically. RUbioSeq's variant analysis results have been already validated and published.	Exome analysis,Copy number estimation,Bisulfite Sequencing	Somatic variant calling	http://seqanswers.com/wiki/RUbioSeq	FASTQ	VCF	Sequence Analysis

447	Breakpointer	Breakpointer is a fast tool for locating sequence breakpoints from the alignment of single end reads (SE) produced by next generation sequencing (NGS). It adopts a heuristic method in searching for local mapping signatures created by insertion/deletions (indels) or more complex structural variants(SVs). With current NGS single-end sequencing data, the output regions by Breakpoint mainly contain the approximate breakpoints of indels and a limited number of large SVs.	Exome and Whole genome variant detection,InDel discovery	Statistical testing	http://seqanswers.com/wiki/Breakpointer	BAM	GFF,GFF3	Sequence Analysis
448	RTG Investigator	Comprehensive analysis pipelines powered with unique mapping speed and sensitivity deliver deep genomic analysis in variant detection and metagenomic applications with Illumina, Ion Torrent, Complete Genomics and Roche 454 data sets.	Exome and whole genome variant detection,metagenomics,SNP discovery,InDel discovery	Mapping,Alignment,translated nucleotide search,k-mer analysis,species frequency estimation,contaminant filtering,Read depth analysis	http://seqanswers.com/wiki/RTG_Investigator	FASTA,FASTQ,SAM,BAM,Complete Genomics	SAM,BAM,BED,VCF	Sequence Analysis
449	Variation toolkit	A set of C++ tools for the interpretation of VCF data.	Genomics,Exome and whole genome variant detection,SNP Annotation		http://seqanswers.com/wiki/Variation_toolkit	VCF,Delimited Text	VCF,Delimited Text,Postscript,FASTA	Sequence Analysis
450	PeakAnalyzer	PeakAnalyzer is a set of applications for processing ChIP signal peaks.	Functional Genomics	ChIP-Seq analysis	http://seqanswers.com/wiki/PeakAnalyzer	ChIP signal	ChIP-Seq analysis	Sequence Analysis

451	SeqSite	SeqSite is an efficient and easy-to-use software tool implementing a novel method for identifying and pinpointing transcription factor binding sites. It first detects transcription factor binding regions by clustering tags and statistical hypothesis testing, and locates every binding site in detected binding regions by modeling the tag profiles. It can pinpoint closely spaced adjacent binding sites from ChIP-seq data. This software is coded in C/C++, and supports major computer platforms.	ChIP-Seq,Functional Genomics,Regulatory element annotation	ChIP-Seq,Peak calling,Statistical Modelling,Statistical testing	http://seqanswers.com/wiki/SeqSite	BED	BED,Bar	Sequence Analysis
452	FusionMap	Detects fusion events in both single- and paired-end datasets from either RNA-Seq or gDNA-Seq studies and characterize fusion junctions at base-pair resolution.	Fusion genes,Fusion transcripts	Split-read	http://seqanswers.com/wiki/FusionMap	FASTA,FASTQ	SAM	Sequence Analysis
453	FusionSeq	Identifies fusion transcripts from paired end RNA-Seq data.	Fusion transcripts,RNA-Seq,Fusion genes,Fusion transcripts	Alignment Analysis	http://seqanswers.com/wiki/FusionSeq	MRF	GFR,BP	Sequence Analysis
454	Trans-ABySS	Trans-ABySS is a software package that is designed to analyze ABySS-assembled whole-genome shotgun transcriptome data.	RNA-Seq,SNP discovery,Fusion genes,InDel discovery,Fusion transcripts		http://seqanswers.com/wiki/Trans-ABySS	FASTQ,ABySS assemblies		Sequence Analysis

455	ViralFusionSeq	Accurately discover viral integration events and fusion transcripts by the use of soft-clipping information, read-pair analysis, and targeted de novo assembly	Genomics,Fusion genes,Fusion transcripts,Viral genomics	Read Alignment,Read mapping,Read pre-processing,Split-read,de-novo assembly,targeted de novo assembly,Alignment,Assembly	http://seqanswers.com/wiki/ViralFusionSeq	Single-end and Paired end,FASTQ,(Compressed) FASTQ	Custom,.fasta,FASTA	Sequence Analysis
456	ChimeraScan	Identifies chimaeric transcripts in RNA-Seq data	Fusion transcripts		http://seqanswers.com/wiki/ChimeraScan			Sequence Analysis
457	FusionHunter	Identifies gene fusions in RNA-Seq data	RNA-Seq,Fusion transcripts		http://seqanswers.com/wiki/FusionHunter	FASTQ		Sequence Analysis
458	FusionMap	Detects fusion events in both single- and paired-end datasets from either RNA-Seq or gDNA-Seq studies and characterize fusion junctions at base-pair resolution.	Fusion genes,Fusion transcripts	Split-read	http://seqanswers.com/wiki/FusionMap	FASTA,FASTQ	SAM	Sequence Analysis
459	FusionSeq	Identifies fusion transcripts from paired end RNA-Seq data.	Fusion transcripts,RNA-Seq,Fusion genes,Fusion transcripts	Alignment Analysis	http://seqanswers.com/wiki/FusionSeq	MRF	GFR,BP	Sequence Analysis
460	ShortFuse	Method for using paired-end reads to find fusion transcripts without requiring unique mappings or additional single read sequencing	Fusion transcripts		http://seqanswers.com/wiki/ShortFuse		Fusion transcripts	Sequence Analysis
461	TopHat-Fusion	Detection of fusion genes in RNA-Seq data	Fusion transcripts		http://seqanswers.com/wiki/TopHat-Fusion	RNA-seq	genes	Sequence Analysis

462	ViralFusionSeq	Accurately discover viral integration events and fusion transcripts by the use of soft-clipping information, read-pair analysis, and targeted de novo assembly	Genomics,Fusion genes,Fusion transcripts,Viral genomics	Read Alignment,Read mapping,Read pre-processing,Split-read,de-novo assembly,targeted de novo assembly,Alignment,Assembly	http://seqanswers.com/wiki/ViralFusionSeq	Single-end and Paired end,FASTQ,(Compressed) FASTQ	Custom,.fasta,FASTA	Sequence Analysis
463	CisGenome	An integrated tool for tiling array, ChIP-seq, genome and cis-regulatory element analysis	ChIP-Seq,ChIP-on-chip,Motif analysis,Gene annotation retrieval,Motif analysis	Gibbs motif sample	http://seqanswers.com/wiki/CisGenome		ChIP-seq analysis, genome analysis	Sequence Analysis
464	Knime4Bio	custom nodes for the interpretation of Next Generation Sequencing data with KNIME.	Genomics,Gene annotation retrieval,mutations and regulatory sites		http://seqanswers.com/wiki/Knime4Bio	VCF,Delimited Text	Various	Sequence Analysis
465	Adapter Removal (software)	Removes adaptor fragments from raw short read sequence data and outputs data to FASTA format.	General bioinformatics (pipeline)	Adapter Removal	http://seqanswers.com/wiki/Adapter_Removal_%28software%29	FASTA,FASTQ	FASTA	Sequence Analysis

466	Bcbio-nextgen	Python scripts and modules for automated next gen sequencing analysis. These provide a fully automated pipeline for taking sequencing results from an Illumina sequencer, converting them to standard Fastq format, aligning to a reference genome, doing SNP calling, and producing a summary PDF of results.	General bioinformatics (pipeline)	QC,filtering,Trimming,Mapping,Peak calling,Motif detection,Differential expression,genomic region matching,Alignment,Genotyping	http://seqanswers.com/wiki/Bcbio-nextgen	FASTQ	FASTQ,SAM,BAM,BED,VCF,PDF	Sequence Analysis
467	SiLoCo	Compares sRNA expression levels in multiple samples by grouping sRNAs into loci based on genomic location	General bioinformatics (pipeline)	Expression profiling	http://seqanswers.com/wiki/SiLoCo	FASTA,FASTQ	CSV,Various image file formats	Sequence Analysis
468	Ta-si prediction	ta-siRNA (trans-acting short interfering RNA): prediction of phased ta-siRNAs in plant sRNA datasets.	General bioinformatics (pipeline)	phase pattern prediction	http://seqanswers.com/wiki/Ta-si_prediction	FASTA,FASTQ	csv,Various image file formats	Sequence Analysis
469	Tripal	Tripal is a collection of open-source, freely-available Drupal modules that serves as a web interface for a GMOD Chado database. It is designed to allow anyone with genomic data to quickly create an online genomic database using community supported tools. Tripal is part of the open-source tool collection available through the Generic Model Organism Database (GMOD) project.	Genomics,Genetics	Visualization,Database interface	http://seqanswers.com/wiki/Tripal	FASTA,GFF3,Chado DB		Sequence Analysis
470	ZORRO	ZORRO is an hybrid sequencing technology assembler. It takes to sets of pre-assembled contigs and merge them into a more contiguous and consistent assembly. The main caracteristic of Zorro is the treatment before and after assembly to avoid errors.	Genomic Assembly,Genomics	Assembly,Hybrid assembly	http://seqanswers.com/wiki/ZORRO	FASTA	FASTA	Sequence Analysis
471	BAMseek	BAMseek is a large file viewer for BAM and SAM alignment files.	Genomics,Transcriptomics	Alignment viewer	http://seqanswers.com/wiki/BAMseek	SAM,BAM,VCF	alignment view	Sequence Analysis
472	BreakDancer	BreakDancer is an application for detecting structural rearrangements and indels in short read sequencing data	Genomics,Structural variation,InDel discovery		http://seqanswers.com/wiki/BreakDancer	genomic structure	structural rearrangments	Sequence Analysis

473	CLEVER	CLEVER is a tool to discover structural variations such as (larger) insertions and deletions in genomes from paired-end sequencing reads.	Genomics,Structural variation,Copy number estimation	structural variation discovery	http://seqanswers.com/wiki/CLEVER	BAM	Tab separated	Sequence Analysis
474	Cortex	Cortex is an efficient and low-memory software framework for analysis of genomes using sequence data. Cortex allows de novo assembly of variants without having to do a consensus assembly first. Also allows comparison of genomes without using consensus, and alignment of sequence data to a de Bruijn graph	Genomics	Assembly,Variant Calling	http://seqanswers.com/wiki/Cortex	FASTQ,FASTA	FASTA-like,VCF	Sequence Analysis
475	DeconSeq	DeconSeq can be used to automatically detect and efficiently remove any type of sequence contamination from metagenomic datasets, including human or other host sequences. The tool uses a modified version of the BWA-SW aligner and can be applied to longer-read datasets (150+bp read length). DeconSeq is available as both standalone and web-based versions.	Metagenomics,Metatranscriptomics,Genomics	Contaminant filtering	http://seqanswers.com/wiki/DeconSeq	FASTA,FASTQ,(Compressed) FASTA,(Compressed) FASTQ	FASTA,FASTQ	Sequence Analysis
476	ERNE	Extended Randomized Numerical alignEr for accurate alignment of NGS reads. It can map bisulfite-treated reads.	Genomics,Alignment,Bisulfite Sequencing	Mapping,Bisulfite mapping	http://seqanswers.com/wiki/ERNE	FASTQ,(Compressed) FASTQ	SAM/BAM	Sequence Analysis
477	Est2assembly	Processes raw sequence data from Sanger or 454 sequencing into a hybrid de-novo assembly, annotates it and produces GMOD compatible output, including a SeqFeature database suitable for GBrowse.	RNA-Seq Alignment,Genomics	RNA-Seq Alignment,Genomics	http://seqanswers.com/wiki/Est2assembly	sequence data	RNA-Seq Alignment,Genomics	Sequence Analysis
478	FastQ Screen	FastQ Screen provides a simple way to screen a library of short reads against a set of reference libraries. Its most common use is as part of a QC pipeline to confirm that a library comes from the expected source, and to help identify any sources of contamination.	Genomics,Transcriptomics	Mapping,Sequencing Quality Control	http://seqanswers.com/wiki/FastQ_Screen	FASTQ	Delimited Text,PNG	Sequence Analysis

479	FindPeaks 4.0 (Vancouver Short Read Package)	The Vancouver Short Read Analysis Package (VSRAP) contains the FindPeaks application for Chip-Seq and RNA-Seq analysis, as well as utilities for SNP finding, working with aligned sequence files and a nascent database for storing SNPs across multiple libraries.,This package includes a growing suite of open source tools for working with Aligned reads of a wide variety of formats. The original version began with the FindPeaks ChIP-Seq tool, but has since grown into a wide variety of tools covering features from ChIP-Seq to RNA-Seq analysis and SNP discovery.,Contributions are welcome and encouraged, and new developers are welcome to join the project.	Genomics,SNP discovery	Peak calling,Database, Format conversion,Alignment Analysis	http://seqanswers.com/wiki/FindPeaks_4.0_%28Vancouver_Short_Read_Package%29	BED,Maq,SAM,Bowtie,Eland,FASTA,Mapview	ACE,BED,GFF,WIG,Peaks, Regions,PNG	Sequence Analysis
480	Flexbar	flexible barcode and adapter processing for next-generation sequencing platforms	Next Generation Sequencing,Sequence Quality Control,Genomics	Read pre-processing,Sample Barcoding,Adapter Removal,Trimming	http://seqanswers.com/wiki/Flexbar	Fasta/q,Csfasta/q	Fasta/q,Csfasta/q	Sequence Analysis
481	Genomic Tools	GenomicTools is a flexible computational platform for the analysis and manipulation of high-throughput sequencing data such as RNA-seq and ChIP-seq. A variety of mathematical operations between sets of genomic regions is implemented thereby enabling the prototyping of computational pipelines that can address a wide spectrum of tasks from preprocessing and quality control to meta-analyses. More specifically, the user can easily create average read profiles across transcriptional start sites or enhancer sites, quickly prototype customized peak discovery methods for ChIP-seq experiments, perform genome-wide statistical tests such as enrichment analyses, design controls via appropriate randomization schemes, among other applications.	Genomics,ChIP-Seq,RNA-Seq	genomic overlaps,peak detection,profiles,heatmaps	http://seqanswers.com/wiki/GenomicTools	BED,SAM/BAM,GFF	BED,SAM,GFF	Sequence Analysis
482	Gk arrays	Gk-arrays are a data structure to index the k-mers in a collection of reads.	Genomics,transcriptomics,Metagenomics	Assembly,Error correction,Mapping	http://seqanswers.com/wiki/Gk_arrays	FASTA,Fastq,Multi-FastA		Sequence Analysis

483	GPS	GPS is a high spatial resolution peak detection algorithm for ChIP-Seq data.	Genomics,ChIP-Seq,Transcription Factor Binding Site identification,regulatory genomics epigenomics	Protein Binding Peak Detection	http://seqanswers.com/wiki/GPS	BED,SAM/BAM,Bowtie,ELAND		Sequence Analysis
484	HeliSphere	Open-source LINUX software package intended for use in analyzing data produced by the HeliScope Single Molecule Sequencer.	Genomics,Whole Genome Resequencing,RNA-Seq,SNP discovery	Mapping	http://seqanswers.com/wiki/HeliSphere	SMS	FASTA,FASTQ,SAM,BAM,BED,WIG	Sequence Analysis
485	HiPipe	HiPipe is to make NGS data analysis quick and easy with high performance pipelines and intuitive web GUI.	Genomics	Mapping,Variant detection,Analysis Pipeline	http://seqanswers.com/wiki/HiPipe	Fastq.gz	VCF BAM	Sequence Analysis
486	Illuminate	Analytics toolkit in Python for Illumina HiSeq and MiSeq metrics,Illuminate parses the metrics binaries that result from Illumina sequencer runs, and provides usable data in the form of python dictionaries and dataframes. Intended to emulate the output of Illumina SAV, illuminate allows you to print sequencing run metrics to the command line as well as work with the data programmatically.,For the latest up-to-date README and status of Illuminate, along with how-tos and sample data, go to one of the following links.	Genomics	Sequencing Quality Control	http://seqanswers.com/wiki/Illuminate	.bin and .xml files from rundata directory	python data structures,some textual output	Sequence Analysis
487	Lab7	Data workflow management platform to streamline NGS analyses 200px|right	Genomics	Workflow,Pipeline Management,Sample Tracking,Protocol Management				Sequence Analysis

488	Lasergene	Lasergene is a comprehensive DNA and protein sequence analysis software suite comprised of seven applications which include functions ranging from sequence assembly and SNP detection, to automated virtual cloning and primer design.	Alignment,De novo sequencing,De-novo assembly,Genomics,InDel discovery,Integrated solution,Mapping,Phylogenetics,Protein structure analysis,Read alignment,SNP discovery,Sequence analysis,Transcription Factor Binding Site identification	Alignment,Alignment Analysis,Annotation,Assembly,Chromatogram viewer,Colorspace,Sequence analysis,Integrated Solution,Mapping,PCR Primer Design,Paired End,Scaffolding	http://seqanswers.com/wiki/Lasergene	DNA, protein sequence	protein sequence analysis, DNA analysis	Sequence Analysis
489	UGENE	UGENE is a free cross-platform genome analysis suite that combines popular bioinformatics tools within a single user friendly interface.	Phylogenetics,Genomics,Sequence analysis,Protein structure analysis	Sequence parsing,command line tool wrappers,Programming Library	http://seqanswers.com/wiki/UGENE	FASTA,GenBank,EMBL,FASTQ,AB1,Stockholm,clustalw,fasta.gz,phylip,MSF,GFF,newick,ACE,SAM	FASTA,GenBank,Stockholm,clustalw,fasta.gz,newick,ACE,SAM	Sequence Analysis

490	Novocraft	Novoalign is a program for mapping short reads from the Illumina/SOLiD sequencing platform(s) to a reference genome.,Novoalign is a fast and accurate short read alignment tool for mapping large amounts of sequencing reads to a reference genome. The software is used for mapping reads from the Illumina and SOLiD sequencing platforms.,NovoalignMPI is our cluster-aware solution that supports parallel execution of alignment jobs across a network of servers using the MPICH2 interface.	Genomics,Whole Genome Resequencing,RNA-Seq Alignment,ChIP-Seq,miRNA	Mapping	http://seqanswers.com/wiki/Novocraft	FASTA,FASTQ,fasta.gz,CSFASTA/CSQUAL (ABI SOLiD)	SAM,Delimited Text,TXT	Sequence Analysis
491	SeqBuster	SeqBuster, a web-based bioinformatic tool offering a custom analysis of deep sequencing data at different levels, with special emphasis on the analysis of miRNA variants or isomiRs and the discovering of new small RNAs.	Small RNA transcriptome,miRNA	Mapping,Annotation	http://seqanswers.com/wiki/SeqBuster	FASTA,Tab-delimited	Tab-delimited	Sequence Analysis
492	SeqSolve	Simple analysis of Next Generation Sequencing data.	RNA-Seq,ChIP-Seq,Transcriptomics,miRNA,ncRNAs,sRNA,Differential Expression,Alternative Splicing,New gene discovery,Differential Expression,Quality Control	SAMtools,Cufflinks,IGV,MACS,Tibco Spotfire	http://seqanswers.com/wiki/SeqSolve	SAM,BAM,BED,GTF,GFF,TXT	JPG,PPT,PDF,BED,XLS,TXT	Sequence Analysis
493	Vicuna	De novo assembly of viral populations	De novo assembly,viral genomics,Population Genomics		http://seqanswers.com/wiki/Vicuna	FASTQ	FASTA	Sequence Analysis

494	ViralFusionSeq	Accurately discover viral integration events and fusion transcripts by the use of soft-clipping information, read-pair analysis, and targeted de novo assembly	Genomics,Fusion genes,Fusion transcripts,Viral genomics	Read Alignment,Read mapping,Read pre-processing,Split-read,de-novo assembly,targeted de novo assembly,Alignment,Assembly	http://seqanswers.com/wiki/ViralFusionSeq	Single-end and Paired end,FASTQ,(Compressed) FASTQ	Custom,.fasta,FASTA	Sequence Analysis
495	VirusSeq	We developed a new algorithmic method, VirusSeq, for detecting known viruses and their integration sites in the human genome using next-generation sequencing data. We evaluated VirusSeq on RNA-Seq data of 256 TCGA human cancer samples. Using these data, we showed that VirusSeq accurately detects the known viruses and their integration sites with high sensitivity and specificity. VirusSeq can also perform this function using whole genome sequencing data of human tissue.	Viral genomics	Mapping,Read mapping,Read Alignment	http://seqanswers.com/wiki/VirusSeq	FASTQ	viral genomics	Sequence Analysis
496	BFAST	Blat-like Fast Accurate Search Tool.	Whole Genome Resequencing	Mapping,Alignment,Genome Indexing,Colorspace	http://seqanswers.com/wiki/BFAST	FASTQ,QSEQ,CSFASTA/CSQUAL (ABI SOLiD),compressed/uncompressed	SAM,BAF	Sequence Analysis

497	SeqMan Ngen	Sequence assembly software using traditional, next-gen, and third-gen techonologies. Subsequent analysis of the assembly, including SNP discovery, coverage evaluation and consensus annotation is provided through full integration with Lasergene.	Genomics,De-novo assembly,De novo transcriptome assembly,Whole Genome Resequencing,SNP discovery,InDel discovery,ChIP-Seq,RNA-Seq Alignment	Mapping,Assembly,Alignment,Paired End	http://seqanswers.com/wiki/SeqMan_Ngen	FASTA,FASTQ,Scarf,SFF,SQD,ACE,PHD,ABI,AB1,GFF,CSFASTA/CSQUAL (ABI SOLiD),SCF,TXT,GenBank,SEQ	BAM,SAM,SQD,ACE,FASTA	Sequence Analysis
498	TAPyR	Efficient BWT-based read aligner supporting multiple sequencing platforms	Whole Genome Resequencing	Read mapping	http://seqanswers.com/wiki/TAPyR	FASTQ,SFF	SAM,BAM	Sequence Analysis
499	Ngs-pipeline	Complete solution for human re-sequencing projects	Personal genomics,regulatory genomics epigenomics,SNP discovery,structural variation discovery,regulatory element annotation,InDel discovery	mapping	http://seqanswers.com/wiki/Ngs-pipeline	BAM,(Compressed) FASTQ,FASTQ,CSFASTA/CSQUAL (ABI SOLiD)	BAM,VCF	Sequence Analysis

500	SeqSite	SeqSite is an efficient and easy-to-use software tool implementing a novel method for identifying and pinpointing transcription factor binding sites. It first detects transcription factor binding regions by clustering tags and statistical hypothesis testing, and locates every binding site in detected binding regions by modeling the tag profiles. It can pinpoint closely spaced adjacent binding sites from ChIP-seq data. This software is coded in C/C++, and supports major computer platforms.	ChIP-Seq,Functional Genomics,Regulatory element annotation	ChIP-Seq,Peak calling,Statistical Modelling,Statistical testing	http://seqanswers.com/wiki/SeqSite	BED	BED,Bar	Sequence Analysis
501	MG-RAST	MG-RAST is a fully-automated service for annotating metagenome samples.	Metagenomics,Phylogenetics,Metabolic reconstruction	Annotation	http://seqanswers.com/wiki/MG-RAST	metagenome	metagenome samples annotation	Sequence Analysis
502	EUADR - Literature analysis	The aim of the "Literature Analysis" workflow is to automate the search of publications related to ADRs corresponding to a given drug/adverse event association. To do so, we defined an approach based on the MeSH thesaurus, using the subheadings «chemically induced» and «adverse effects» with the “Pharmacological Action” knowledge.		literature analysis, ADR, adverse event, adverse effects	http://www.myexperiment.org/workflows/2280.html	Event Code, ATC code of the drug	List of MedLine publications found formatted according the defined EU-ADR project format. List of adverse events	
